# Suppression of transcription-replication conflicts by sequence-coordinated actions of TRDMT1 and MutLα

**DOI:** 10.1038/s41467-026-76137-8

**Published:** 2026-08-10

**Authors:** Arijit Ghosh, Xiaojuan Ran, Fengqi Zhang, Boya Gao, Yu Xu, Lai-Yee Phoon, Melissa Long, Mingchao Wang, Varsha Ananthapadmanabhan, Ajinkya Kawale, Yug Shah, Brenda Manzano-Winkler, Christopher J. Ott, Lee Zou, Li Lan

**Affiliations:** 1 Department of Pharmacology and Cancer Biology, Duke University School of Medicine, Durham, NC USA; 2 Department of Molecular Genetics and Microbiology, Duke University School of Medicine, Durham, NC USA; 3 Krantz Family Center for Cancer Research, Mass General Brigham, Harvard Medical School, Charlestown, MA USA

**Keywords:** Stalled forks, Targeted therapies, DNA damage and repair

## Abstract

TRDMT1 is an RNA methyltransferase that catalyzes 5-methylcytosine (m5C) formation in R-loops to promote transcription-coupled homologous recombination (TC-HR). Although TRDMT1 inhibition selectively kills BRCA1-deficient cancer cells, broader cancer dependencies on TRDMT1 remain unclear. Here, a TRDMT1 inhibitor (TRDMT1i) sensitivity screen across a large panel of cancer cell lines identifies loss of MLH1 or PMS2, two components of the MutLα mismatch repair (MMR) complex frequently inactivated in tumors, as key determinants of TRDMT1 dependency. In contrast, MutLβ and MutSα/β are dispensable for TRDMT1i resistance, revealing a unique MMR-independent function of MutLα. Mechanistically, TRDMT1 and MutLα independently recognize DNA–RNA hybrids and cooperatively suppress co-transcriptional R-loops genome-wide in undamaged cells, with m5C directing pathway choice. Furthermore, MutLα suppresses R-loops through its ATPase and endonuclease activities and through recruitment of EXO1. Combined loss of TRDMT1 and MLH1 causes extensive R-loop accumulation and transcription replication conflicts (TRCs), impairing replication fork progression, inducing DNA damage, and driving apoptosis-mediated synthetic lethality. Importantly, TRDMT1i suppresses growth of MLH1-deficient tumors by inducing TRCs in vivo, suggesting a potential therapeutic strategy for targeting MutLα-deficient tumors. These studies not only expand our understanding of cancer dependency on TRDMT1, but also identify a promising strategy to exploit TRCs in cancer therapy.

## Abstract (web summary)

DNA strand break repair can be assisted by RNA dependent mechanism. Here the authors show that the RNA methyltransferase TRDMT1 and the mismatch repair complex MutLα independently recognize DNA-RNA hybrids and cooperatively suppress co-transcriptional R-loops, with m5C directing pathway choice.

## Introduction

DNA strand breaks in the genome, especially in actively transcribed genes, impose a severe threat to genomic instability and cell survival^1^. Studies by others and us reveal that DNA break repair in actively transcribed regions is facilitated by transcription- and RNA-dependent mechanisms^2–11^. By forming transient DNA–RNA hybrids or R-loops, which are three-stranded polynucleotide structures consisting of DNA–RNA hybrids and displaced single-stranded DNA (ssDNA), RNA transcripts promote the recruitment of DNA repair proteins and formation of repair intermediates, thereby enhancing the repair in transcribed regions^12^. Some of these transcription- and RNA-dependent repair mechanisms involve the RAD51 recombinase and its regulators, leading to the concept of TC-HR ^2–4,7–9,11^. Investigations on the functions, regulation, and physiological/pathological impact of TC-HR constitute an active area of research in the field of DNA repair and genome maintenance.

Our recent studies revealed that RNA 5-methylcytosine (m5C) is induced at sites of DNA damage and is required for TC-HR^13^. Furthermore, we identified TRDMT1 as the RNA methyltransferase that generates m5C at DNA damage sites. We showed that TRDMT1 is a unique RNA methyltransferase that specifically modifies RNA in DNA–RNA hybrids or R-loops, explaining how it functions at DNA damage-induced R-loops. The m5C generated by TRDMT1 in DNA damage-induced R-loops presents an RNA-based ‘DNA damage mark’ to enhance the recruitment of certain DNA repair proteins and regulators of R-loop metabolism^13–16^, thereby stimulating the process of TC-HR. Depletion of TRDMT1 in some cancer cell lines reduces their viability and increases their sensitivity to DNA damage^13,16^, suggesting that certain cancer cells are increasingly dependent on TC-HR for coping with DNA damage and survival. Interestingly, a cancer patient who had an exceptional response to cisplatin, a cancer drug inflicting DNA damage, was found to carry a mutation in TRDMT1 that renders the protein unstable due to elevated ubiquitination^17^. These findings suggest that TC-HR, and TRDMT1 in particular, may be a good therapeutic target in cancers relying on this specific repair pathway, prompting us to develop inhibitors of TRDMT1 and test their effects in cancer cells.

Through a chemical screen and subsequent structure-directed optimization, we have developed the first specific inhibitor of TRDMT1 (YW1842)^17^. This TRDMT1 inhibitor (TRDMT1i) effectively blocks TRDMT1-mediated m5C formation in DNA–RNA hybrids in vitro and reduces the viability of several cancer cell lines. Notably, we found that BRCA1-deficient cancer cells are highly sensitive to TRDMT1i^16^, suggesting that homologous recombination deficiency (HRD) is a cause of TRDMT1 dependency in cancer and the consequent TRDMT1i sensitivity. This observation is consistent with the role of TRDMT1 in TC-HR, which can occur independently of BRCA1 and act in parallel with the canonical HR pathway to promote RAD51 localization to DNA damage sites^8^. Although our results have linked DNA damage and HRD to TRDMT1i sensitivity, a comprehensive understanding of the TRDMT1 dependency of cancers is still lacking. To understand the causes of TRDMT1 dependency and TRDMT1i sensitivity in cancers, we used a large panel of cancer cell lines to perform a TRDMT1i sensitivity screen in this study.

From this screen, we identify a subset of mismatch repair (MMR) genes whose deficiency confers TRDMT1i sensitivity. Specifically, cancer cell lines deficient for MLH1 and PMS2, two components of the MutLα complex^18,19^, are more sensitive to TRDMT1i than those proficient for the two proteins. In contrast, loss of other MMR complexes is not associated with increased TRDMT1i sensitivity, suggesting a unique and MMR-independent function of MutLα, whose loss confers TRDMT1 dependency. Unlike the R-loops induced by DNA damage, which promote TC-HR, the aberrant accumulation of transcription-coupled R-loops interferes with DNA replication and induces DNA damage^12,20,21^. Simultaneous loss of TRDMT1 and MLH1 results in extensive R-loop accumulation, high levels of transcription-replication conflicts (TRCs), and a severe slowdown of replication forks. These effects are robustly induced by TRDMT1i in MutLα-deficient cancer cells, leading to apoptosis-mediated synthetic lethality. By analyzing R-loops genome-wide, we find that TRDMT1 and MLH1 suppress distinct but overlapping subsets of R-loops, with m5C making the choice between the two pathways. The function of MutLα in R-loop suppression is dependent on its ATPase and nuclease activities, as well as EXO1, which is recruited to R-loops by MutLα. Furthermore, using an MLH1-deficient tumor model, we demonstrate that TRDMT1i increases TRCs in MLH1-deficient tumors in vivo and inhibits their growth. Together, these results provide an extended view of the TRDMT1 dependency of cancers, uncover sequence-directed coordination of distinct TRC suppressors, and bring about a mechanism-based opportunity for applying TRDMT1i to biomarker-guided, targeted cancer therapy.

## Results

### A TRDMT1i sensitivity screen in cell lines identifies TRDMT1 dependency in cancers

**Fig. 1:**
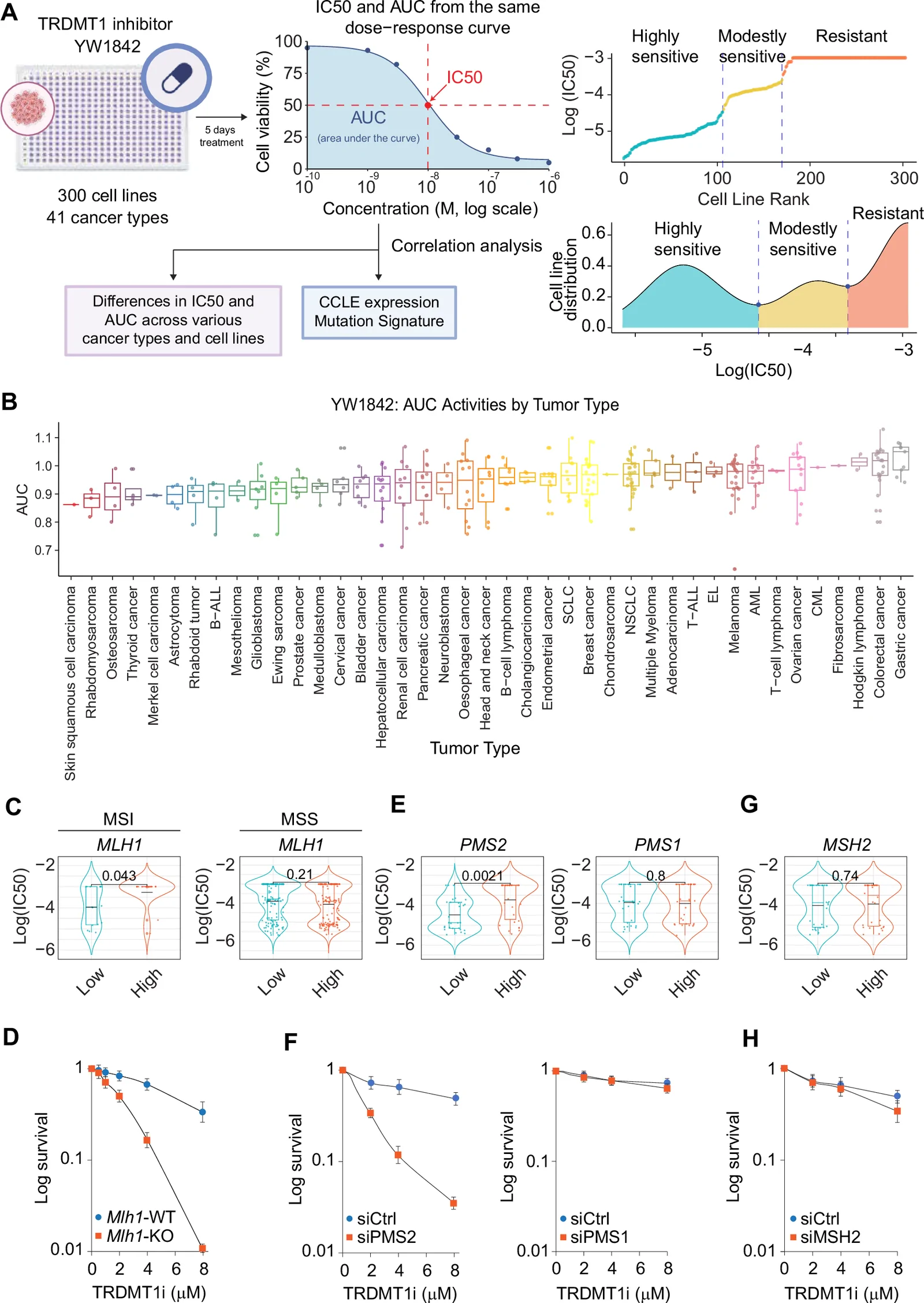
TRDMT1i sensitivity screen identifies MLH1 and PMS2 as suppressors of TRDMT1i sensitivity. **A** Schematic workflow of the TRDMT1i sensitivity screen. Cell lines were classified into three groups based on TRDMT1i LogIC50 values: highly sensitive (LogIC50 ≤ –4.43), moderately sensitive (–4.43 <LogIC50 ≤ –3.59), and resistant (LogIC50 > –3.59). The screen was performed in two biological replicates (*n* = 2). **B** Box plot showing the distribution of TRDMT1i sensitivity across 41 tumor types, ranked by mean AUC for each tumor type. Each dot represents one cancer cell line. Box plots show the median (centre line), the first and third quartiles (box limits), and whiskers extending to the most extreme data points within 1.5 × the interquartile range (IQR). Dots outside the whiskers are individual outliers. **C** Violin plots comparing TRDMT1i sensitivity between MLH1-low and MLH1-high cell lines, classified using the median (50%) cutoff in MSI or MSS cell line groups. Each dot represents one cell line. The violin width represents the kernel density distribution of the data. Box plots show the median (centre line), the first and third quartiles (box limits), and whiskers extending to the most extreme observations within 1.5 × the IQR. The black horizontal line indicates the mean. Statistical significance was determined using a two-sided Wilcoxon rank-sum test. Exact *P* values are shown. **D** Colony formation assay of CT26 *Mlh1-WT* and *Mlh1-KO* cells treated with the indicated doses of TRDMT1i. *n* = 3 biological replicates. Data are presented as mean values ± SEM. **E** Violin plots comparing TRDMT1i sensitivity between cell lines with low or high *PMS2* and *PMS1* expression. Cell lines harboring mutations and within the bottom 10% of expression were defined as the low-expression group, whereas cell lines within the top 10% were defined as the high-expression group. Each dot represents one cell line. The violin width represents the kernel density distribution of the data. Box plots show the median (centre line), the first and third quartiles (box limits), and whiskers extending to the most extreme observations within 1.5× the IQR. The black horizontal line indicates the mean. Statistical significance was determined using a two-sided Wilcoxon rank-sum test. Exact *P* values are shown. **F** Colony formation assay of CT26 cells transfected with control, PMS2, or PMS1 siRNA and treated with the indicated doses of TRDMT1i. *n* = 3 biological replicates. Data are presented as mean values ± SEM. **G** Violin plots comparing TRDMT1i sensitivity between cell lines with low or high *MSH2* expression. Cell lines harboring mutations and within the bottom 10% of expression were defined as the low-expression group, whereas cell lines within the top 10% were defined as the high-expression group. Each dot represents one cell line. The violin width represents the kernel density distribution of the data. Box plots show the median (centre line), the first and third quartiles (box limits), and whiskers extending to the most extreme observations within 1.5× the IQR. The black horizontal line indicates the mean. Statistical significance was determined using a two-sided Wilcoxon rank-sum test. Exact *P* values are shown. **H** Colony formation assay of CT26 cells transfected with control or MSH2 siRNA and treated with the indicated doses of TRDMT1i. *n* = 3 biological replicates. Data are presented as mean values ± SEM. **A** was created with BioRender.com. Publication license: (https://BioRender.com/m97joso).

To investigate whether TRDMT1 inhibition selectively kills cancer cells associated with specific vulnerabilities, we performed a TRDMT1i sensitivity screen using a large panel of cancer cell lines (Fig. 1A). IC50 and AUC values of TRDMT1i (YW1842) were determined across 300 cell lines of 41 tumor types. Our analysis reveals a wide range of TRDMT1i sensitivity across cell lines (Fig. 1A, B). Several tumor types associated with high genomic instability, such as osteosarcoma and endometrial squamous cell carcinoma^22,23^, are among the most TRDMT1i sensitive. Cell lines of other tumor types associated with high genomic instability, such as ovarian adenocarcinoma and breast carcinoma^24,25^, display a range of TRDMT1i sensitivity. These results show that the cytotoxicity of TRDMT1i is selective to certain subsets of cancers. Furthermore, both tumor-type-specific and sub-tumor-type vulnerabilities may contribute to TRDMT1i sensitivity.

To understand the relationship between TRDMT1 expression and TRDMT1i sensitivity, we analyzed the CCLE database and compared groups of TRDMT1-low and -high cell lines stratified according to *TRDMT1* mRNA levels (Supplementary Fig. 1A). As a group, cell lines with high TRDMT1 expression are significantly more sensitive to TRDMT1i compared to those with low TRDMT1 expression, suggesting that TRDMT1i preferentially kills TRDMT1-high cancer cells (Supplementary Fig. 1A). This observation is consistent with the notion that high expression of *TRDMT1* in cancer cells is generally associated with TRDMT1 dependency. Moreover, this result suggests that the effects of TRDMT1i in cancer cell lines are associated with its target TRDMT1.

We next extended our analysis to the status of HR in cancer cell lines, as we previously showed that loss of BRCA1 confers TRDMT1i sensitivity^16^. Because the number of *BRCA1* mutant cell lines in CCLE is small, we tested whether *BRCA1* expression affects TRDMT1i sensitivity. When cell lines are grouped according to levels of *BRCA1* expression, BRCA1-low cell lines are significantly more sensitive to TRDMT1i than BRCA1-high lines (Supplementary Fig. 1B). When cell lines are grouped according to TRDMT1i sensitivity (Fig. 1A), the highly TRDMT1i-sensitive cell lines express significantly lower levels of BRCA1 than those resistant to TRDMT1i (Supplementary Fig. 1C). Furthermore, when cell lines are grouped according to the levels of the mutational signature SBS3, which is associated with HRD^26^, SBS3-high cell lines are more sensitive to TRDMT1i than those with low SBS3 (Supplementary Fig. 1D). Consistent with the idea that TRDMT1i preferentially kills HR-deficient cancer cells, the TRDMT1i sensitivity of cancer cell lines is positively correlated with the sensitivity to two PARP inhibitors (PARPis), Rucaparib and Talazoparib (Supplementary Fig. 1E, F). When cell lines are grouped according to TRDMT1i sensitivity, cell lines with high TRDMT1i sensitivity are also more sensitive to PARPis (Supplementary Fig. 1G, H). Thus, in a large panel of cancer cell lines, TRDMT1i sensitivity is generally associated with HRD, supporting the idea that HRD is a cause of TRDMT1 dependency in cancer cells. Nonetheless, many TRDMT1i-sensitive cell lines are not apparently HRD, raising the possibility that other DNA repair defects or sources of genomic instability also confer TRDMT1i sensitivity.

To investigate whether TRDMT1i sensitivity is generally associated with high chromosomal instability (CIN), we compared the CIN and aneuploidy scores of cell line groups that are highly sensitive or resistant to TRDMT1i. The cell lines highly sensitive to TRDMT1i display higher CIN and aneuploidy scores than those resistant (Supplementary Fig. 1I, J), suggesting that cancer cells with high CIN are generally more sensitive to TRDMT1i.

### Loss of MLH1 and PMS2 confers TRDMT1i sensitivity

To extend our analysis to other cancer-associated DNA repair defects, we examined the relationship between TRDMT1i sensitivity and MMR deficiency (MMR-d). Because MMR-d gives rise to microsatellite instability (MSI) in cancers^27^, we first tested whether MSI was associated with TRDMT1i sensitivity. When cancer cell lines are divided into MSI and microsatellite stable (MSS) groups, MSI is not associated with high TRDMT1i sensitivity (Supplementary Fig. 2A). To investigate the possible contributions of specific MMR defects to TRDMT1i sensitivity, we divided MSI cancer cell lines into MLH1-low and MLH1-high groups. Among the MSI cell lines, low expression of *MLH1* is associated with low TRDMT1i IC50 values (Fig. 1C), suggesting that MLH1 deficiency increases TRDMT1i sensitivity. In contrast, low *MLH1* expression in MSS cell lines, which is insufficient to cause MSI, is not associated with high TRDMT1i sensitivity (Fig. 1C). These results suggest that the MSI caused by MLH1 deficiency, but not general MSI, is associated with high TRDMT1i sensitivity. To directly test whether loss of MLH1 confers TRDMT1i sensitivity, we used CRISPR/Cas9 to knock out *Mlh1* in the mouse colon cancer cell line CT26 (Supplementary Fig. 2B)^28^. *Mlh1-KO* cells are significantly more sensitive to TRDMT1i than *Mlh1* wild-type (*Mlh1-WT*) cells (Fig. 1D). Similarly, knockdown of MLH1 in human osteosarcoma cell line U2OS with siRNA also increases TRDMT1i sensitivity (Supplementary Fig. 2C, D). Together, these results show that loss of MLH1 is indeed a cause of TRDMT1i sensitivity. However, MSI is not generally associated with TRDMT1i sensitivity, raising the possibility that some other MMR defects do not contribute to the TRDMT1 dependency.

To investigate which MMR proteins suppress TRDMT1i sensitivity, we extended our analysis to PMS2 and PMS1, two partners of MLH1 in MutLα and MutLβ complexes^29^. Cancer cell lines harboring *PMS2* mutations or displaying low *PMS2* expression are substantially more sensitive to TRDMT1i than those without *PMS2* mutations and displaying high PMS2 expression (Fig. 1E). In contrast, *PMS1* mutations and low expression do not associate with high TRDMT1i sensitivity (Fig. 1E). Indeed, when PMS2 and PMS1 were knocked down in CT26 and U2OS cells, only the depletion of PMS2 but not that of PMS1 increases TRDMT1i sensitivity (Fig. 1F and Supplementary Fig. 2E–J). These results suggest that MutLα, but not MutLβ, has a specific role in suppressing TRDMT1i sensitivity.

During MMR, MutLα is recruited to DNA mismatches and loops by MutSα/β complexes^30,31^. To test whether MutSα/β complexes are involved in the suppression of TRDMT1i sensitivity, we analyzed the potential effects of deficiency of MSH2, a common component of MutSα/β complexes, in cancer cell lines. *MSH2* mutations and low expression do not associate with high TRDMT1i sensitivity in the cell line panel (Fig. 1G). Furthermore, knockdown of MSH2 in CT26 and U2OS cells does not increase TRDMT1i sensitivity (Fig. 1H, and Supplementary Fig. 2K–M). Together, these results show that MutLα suppresses TRDMT1i sensitivity independently of MutSα/β, suggesting an MMR-independent function of MutLα whose loss renders cancer cells increasingly dependent on TRDMT1 for survival.

### TRDMT1 and MLH1 act in concert to counter transcription-replication conflicts

The selective killing of MutLα-deficient cells by TRDMT1i suggests that TRDMT1i induces synthetic lethality in cells lacking MutLα. The specificity of this TRDMT1i effect towards MutLα deficiency, but not MutLβ and MutS loss, suggests that the cause of synthetic lethality is not general MMR-d. To understand the molecular basis of the synthetic-lethal relationship between TRDMT1 and MutLα, we used DNA fiber assay to examine the impact of their loss on DNA replication, a process that intersects with multiple DNA repair pathways.

**Fig. 2:**
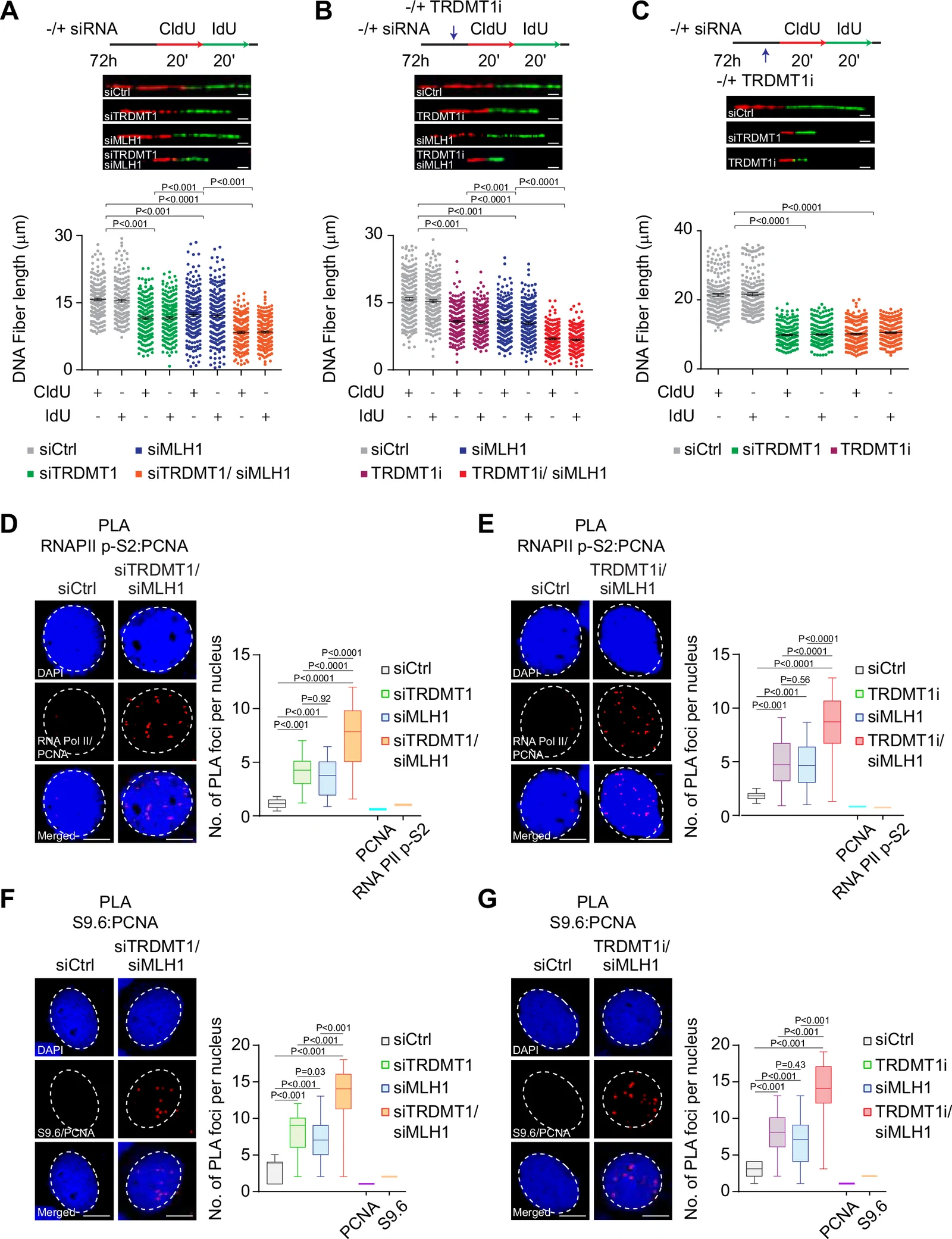
TRDMT1 and MLH1 deficiencies induce replication stress and TRCs. **A**,** B** Experimental scheme, representative images, and quantification of DNA fiber length in U2OS cells transfected with control, TRDMT1, or MLH1 siRNAs, or treated with TRDMT1i as indicated. Lengths of individual CldU- and IdU-labeled replication tracts are shown in scatterplots (*n* = 3 biological replicates, 60–70 fibers per replicate). Data are presented as mean values ± SEM. Scale bar, 10 μm. **C** Experimental scheme, representative images, and quantification of DNA fiber length in HCT116 cells transfected with control or TRDMT1 siRNA, or treated with TRDMT1i as indicated (*n* = 3 biological replicates, 60–70 fibers per replicate). Data are presented as mean values ± SEM. Scale bar, 10 μm. **D**,** E** Representative images and quantification of PLA foci in U2OS cells using anti-RNAPII p-S2 and anti-PCNA antibodies, alone or in combination. Cells were transfected with the indicated siRNAs or treated with TRDMT1i, and nuclear PLA foci are quantified in box plots (*n* = 3 biological replicates, 150–160 cells per replicate). Scale bar, 5 μm. **F**,** G** Representative images and quantification of PLA foci in U2OS cells using anti-S9.6 and anti-PCNA antibodies, alone or in combination. Cells were transfected with the indicated siRNAs or treated with TRDMT1i as indicated, and nuclear PLA foci are quantified in box plots (*n* = 3 biological replicates, 150–160 cells per replicate). Scale bar, 5 μm. For all box plots, the center line represents the median, the lower and upper bounds of the boxes represent the 25th and 75th percentiles, respectively, and the whiskers represent the minimum and maximum values. Statistical significance was determined by two-tailed unpaired Student’s *t* tests to generate *P* values. All numeric values are available in the Source Data.

In DNA fiber assay, nascent DNA was sequentially labeled with thymidine analogs 5-chloro-2′-deoxyuridine (CldU) and 5-iodo-2′-deoxyuridine (IdU), and the progression of DNA replication forks was measured by the lengths of CldU- or IdU-labeled DNA in double-positive replication tracts generated by progressing replication forks. Knockdown of either TRDMT1 or MLH1 slows down fork progression (Fig. 2A, Supplementary Figs. 2C, and S3A), revealing their roles in maintaining replication fork speed. TRDMT1i also reduces fork speed (Fig. 2B), suggesting that the catalytic activity of TRDMT1 is required. Notably, replication fork speed is further reduced when TRDMT1 and MLH1 were co-depleted (Fig. 2A), when MLH1 knockdown cells were treated with TRDMT1i (Fig. 2B), or when MLH1-deficient HCT116 cells were treated with TRDMT1 siRNA or TRDMT1i (Fig. 2C and Supplementary Fig. 3B)^32^, suggesting that TRDMT1 and MLH1 have non-redundant functions in maintaining replication fork speed. Thus, loss of both TRDMT1 and MLH1 results in a severe replication defect.

To understand why replication forks slowdown in the absence of TRDMT1 and MLH1, we analyzed the levels of transcription-replication conflicts (TRCs) using a proximity ligation assay (PLA). In this PLA, the collisions between transcription and replication complexes are measured by the colocalization of elongating RNA polymerase II (RNAPII p-Ser2) and PCNA, a component of the replisome. Nuclear PLA foci are detected only when both RNAPII p-Ser2 and PCNA antibodies are used together, but not when either antibody is used alone (Fig. 2D, E), confirming the specificity of the assay. Knockdown of either TRDMT1 or MLH1 increases PLA foci (Fig. 2D and Supplementary Fig. 3E), suggesting functions of TRDMT1 and MLH1 in suppressing TRCs. Simultaneous knockdown of TRDMT1 and MLH1 further increases PLA foci (Fig. 2D and Supplementary Fig. 3E), showing that their functions in TRC suppression are non-redundant. Consistently, PLA foci are increased by TRDMT1i treatment alone and further increased in MLH1 knockdown cells after TRDMT1i treatment (Fig. 2E and Supplementary Fig. 3F). A brief treatment of cells with Triptolide, an inhibitor of RNAPII, substantially reduces PLA foci (Supplementary Fig. 3G), confirming the transcription dependency of TRCs. Consistent with the presence of TRCs in cells, PCNA is detected on the chromatin fragments captured by S9.6 immunoprecipitation (Supplementary Fig. 3H). While the PCNA on R-loop-containing chromatin is increased by knockdown of TRDMT1 and MLH1, it is eliminated by RNaseH1 overexpression (Supplementary Fig. 3H), supporting the notion that TRDMT1 and MLH1 suppress TRCs. Thus, TRDMT1 and MLH1 function in concert to suppress TRCs and maintain replication fork progression.

Aberrant accumulation of R-loops exacerbates TRCs^33–35^. To monitor the collisions between replication forks and R-loops, we performed PLA using S9.6 and PCNA antibodies (Fig. 2F, G). The ability of S9.6 to recognize DNA–RNA hybrids allows this PLA to detect the replisomes encountering R-loops. Knockdown of TRDMT1 or MLH1 alone increases PLA foci in U2OS cells, and co-depletion of both proteins further elevates PLA focus levels (Fig. 2F and Supplementary Fig. 3I). Similarly, PLA foci are increased by TRDMT1i treatment alone and further increased in MLH1 knockdown cells after TRDMT1i treatment (Fig. 2G and Supplementary Fig. S3J). Together, these results suggest that TRDMT1 and MLH1 have non-redundant functions in suppressing R-loop-mediated TRCs.

To investigate how cells die after loss of TRDMT1 and MLH1, we performed immunofluorescence analysis of γH2AX in cells treated with TRDMT1i and/or MLH1 siRNA (Supplementary Fig. 4A). TRDMT1 inhibition or MLH1 knockdown alone elevates γH2AX levels, and the combination increases γH2AX further. Moreover, γH2AX levels in TRDMT1i-treated MLH1 knockdown cells are reduced by RNaseH1 overexpression (Supplementary Fig. 4A), showing that R-loop-induced DNA damage is increased upon loss of both TRDMT1 and MLH1. The treatment of MLH1 knockdown cells with TRDMT1i also induces cleavage of PARP and Caspase 3 (Supplementary Fig. 4B), indicating the activation of apoptosis. Consistently, the fraction of cells double-positive for Annexin V and Propidium Iodide (PI) staining is increased in the cell population upon loss of TRDMT1 and MLH1 (Supplementary Fig. 4C, D). Thus, concurrent loss of TRDMT1 and MLH1 leads to a surge of R-loop-induced DNA damage and cell death through apoptosis.

### TRDMT1 and MLH1 function jointly to suppress R-loops

**Fig. 3:**
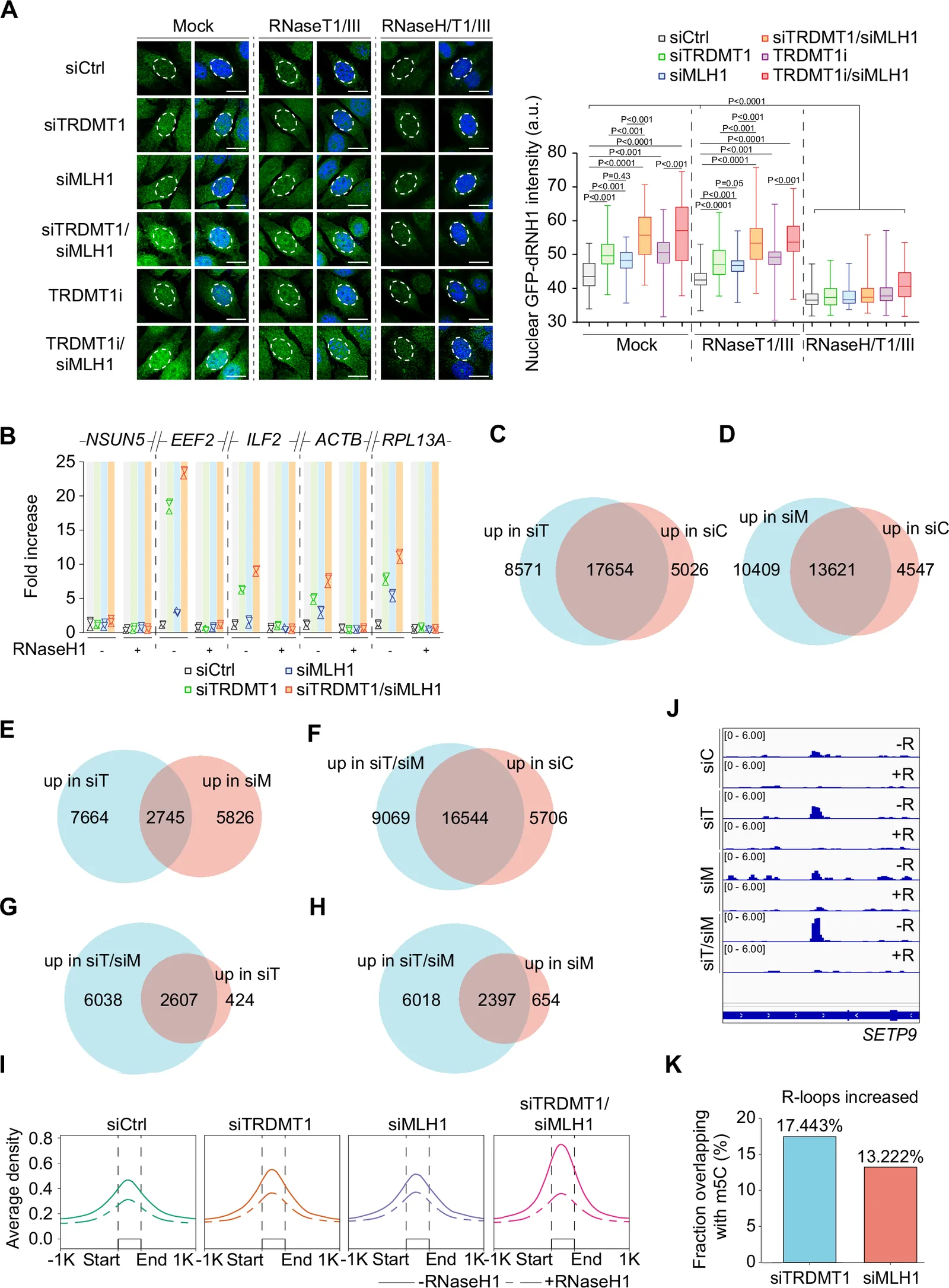
TRDMT1 and MLH1 cooperate to suppress R-loops. **A** Representative images of HeLa cells analyzed for R-loops by GFP-dRNH1 staining (top). Cells were transfected with the indicated siRNAs or treated with TRDMT1i. Fixed cells were treated with RNases T1, III, and H to remove single-stranded RNA, double-stranded RNA, and DNA–RNA hybrids, respectively. Quantification of mean nuclear GFP-dRNH1 intensity is shown in box plots (*n* = 3 biological replicates, 20 cells per replicate). For the box plot, the center line represents the median, the lower and upper bounds of the boxes represent the 25th and 75th percentiles, respectively, and the whiskers represent the minimum and maximum values. Statistical significance was determined by two-tailed unpaired Student’s *t* tests to generate *P* values. Scale bar, 5 μm. a.u. arbitrary unit. **B** DRIP-qPCR analysis of R-loops at the *NSUN5, EEF2, ILF2, ACTB*, and *RPL13A* loci in HeLa cells transfected with the indicated siRNAs and left uninduced or induced for RNaseH1 expression. *n* = 2 biological replicates (3 independent sets of technical replicates). Datapoints from different biological replicates are shown as symbols of different shapes. **C**–**H** Venn diagrams showing numbers of R-loop peaks unique to or shared among cell populations transfected with the indicated siRNAs or treated with TRDMT1i. **I** Aggregated profiles of R-loop peaks in the indicated cell populations with (dotted lines) or without (solid lines) RNaseH1 induction. **J** IGV browser tracks showing representative R-loop peaks upregulated in cells transfected with siRNAs targeting TRDMT1, MLH1, or both. **K** Bar graph showing the fraction of R-loops overlapping with m5C sites in cells transfected with siRNAs targeting TRDMT1 or MLH1.

To directly test whether TRDMT1 and MLH1 suppress R-loops, we analyzed R-loops by cell staining with purified GFP-tagged, catalytically inactive RNaseH1 (GFP-dRNH1)^36^. Nuclear GFP signals are increased by knockdown of TRDMT1 or MLH1 alone, and further increased by co-depletion of both proteins (Fig. 3A). Similarly, TRDMT1i alone increases nuclear GFP signals in cells treated with control siRNA, and further increases the signals in MLH1 knockdown cells (Fig. 3A). Notably, treatment of cells with RNases T1 and III, which remove double-stranded RNA (dsRNA), slightly reduces nuclear GFP signals (Fig. 3A), suggesting that most of the GFP signals do not come from dsRNA. Importantly, the nuclear GFP signals are largely eliminated by the treatment with RNases T, III, and H (Fig. 3A), confirming that most of the GFP signals are indeed R-loop dependent. These results demonstrate that TRDMT1 and MLH1 have non-redundant functions in R-loop suppression.

To confirm the suppression of R-loops by TRDMT1 and MLH1, we performed DNA–RNA Immunoprecipitation coupled with qPCR (DRIP-qPCR) to quantitatively measure the levels of R-loops at specific genomic loci. We previously reported that TRDMT1-mediated m5C formation was enriched in GC-rich and high-GC-skew sequences prone to form R-loops^16^. At two loci (*EEF2* and *ILF2*) where both R-loops and TRDMT1-dependent m5C were detected, knockdown of TRDMT1 increases R-loops substantially (Fig. 3B and Supplementary Fig. 5A). Knockdown of MLH1 increases R-loops to much lesser extents but further elevates R-loop levels in TRDMT1 knockdown cells (Fig. 3B and Supplementary Fig. 5A). At *ACTB* and *RPL13A* loci where R-loops but not m5C was detected, knockdown of TRDMT1 and MLH1 alone increases R-loops modestly and similarly, and co-depletion of both proteins induces a further increase (Fig. 3B and Supplementary Fig. 5A). These increases of R-loops are suppressed by expression of wild-type RNaseH1 (RNaseH1-WT), and they occur at much lower levels at a gene that is not actively transcribed (*NSUN5*) (Fig. 3B and Supplementary Fig. 5A). Thus, both TRDMT1 and MLH1 suppress R-loop formation at active genes. Notably, the function of TRDMT1 is more prominent at the loci where m5C occurs efficiently, supporting the idea that TRDMT1 suppresses R-loops by generating m5C.

To comprehensively understand the effects of TRDMT1 and MLH1 on R-loops in the genome, we performed CUT&TAG using the S9.6 antibody to analyze R-loops throughout the genome. Knockdown or inhibition of TRDMT1 results in an overall increase of R-loops in the genome (Fig. 3C and Supplementary Fig. 5B). Approximately one third of the R-loops detected in TRDMT1 knockdown cells are either unique or increased compared to control cells. Similarly, knockdown of MLH1 also leads to an overall increase of R-loops in the genome (Fig. 3D). Nearly half of the R-loops detected in MLH1 knockdown cells are either unique or increased compared to control cells. Notably, when the R-loops in TRDMT1 and MLH1 knockdown cells were compared, most of the R-loops are either unique or upregulated in TRDMT1 knockdown cells, or unique or upregulated in MLH1 knockdown cells (Fig. 3E), suggesting that these two proteins have non-redundant roles in R-loop suppression. However, when TRDMT1 and MLH1 were co-depleted, the fraction of unique and upregulated R-loops in double knockdown cells compared to control cells is similar to those in TRDMT1 or MLH1 single knockdown cells (Fig. 3F), suggesting that TRDMT1 and MLH1 affect similar or substantially overlapping subsets of R-loops. Similar observations are made in TRDMT1i-treated MLH1 knockdown cells (Supplementary Fig. 5C). Together, these results suggest that TRDMT1 and MLH1 regulate overlapping subsets of R-loops, but their effects on most of these R-loops are distinct.

Consistent with the non-redundant functions of TRDMT1 and MLH1 in R-loop suppression, double knockdown of TRDMT1 and MLH1 further increases R-loops compared to TRDMT1 or MLH1 knockdown alone (Fig. 3G, H). TRDMT1i treatment of MLH1 knockdown cells also further increases R-loops compared to TRDMT1i treatment or MLH1 knockdown alone (Supplementary Fig. 5D, E). Quantification of aggregated CUT&TAG signals at R-loop peaks confirms the increase of R-loops by knockdown of TRDMT1 or MLH1 alone, as well as the further increase of R-loops by co-depletion of TRDMT1 and MLH1 (Fig. 3I). Importantly, the CUT&TAG signals at R-loop peaks are reduced by expression of RNaseH1-WT, confirming the specificity of the assay (Fig. 3I). Similar observations are made in cells treated with TRDMT1i, MLH1 siRNA, and the combination of the two (Supplementary Fig. 5F). Analysis of CUT&TAG signals at individual R-loops identifies examples of R-loops that are similarly increased by TRDMT1 and MLH1 depletions, or preferentially increased by loss of either TRDMT1 or MLH1 (Fig. 3J and Supplementary Fig. 5G–I). Collectively, these results provide an explanation of how simultaneous loss of TRDMT1 and MLH1 exacerbates R-loop accumulation in the genome.

Our DRIP-qPCR results suggest that TRDMT1 preferentially acts on the R-loops where m5C can form efficiently (Fig. 3B). To verify this finding, we tested whether the R-loops unique or upregulated in TRDMT1 knockdown cells are associated with the TRDMT1-dependent m5C signals in the genome^16^. Indeed, the R-loops increased by TRDMT1 knockdown display a greater association with m5C compared to those increased by MLH1 depletion (Fig. 3K), supporting the idea that the propensity of m5C formation at individual R-loops is a determinant of the relative contribution of TRDMT1 to R-loop suppression at these loci.

### R-loop-mediated TRCs and fork slowdown in TRDMT1- and MLH1-deficient cells

**Fig. 4:**
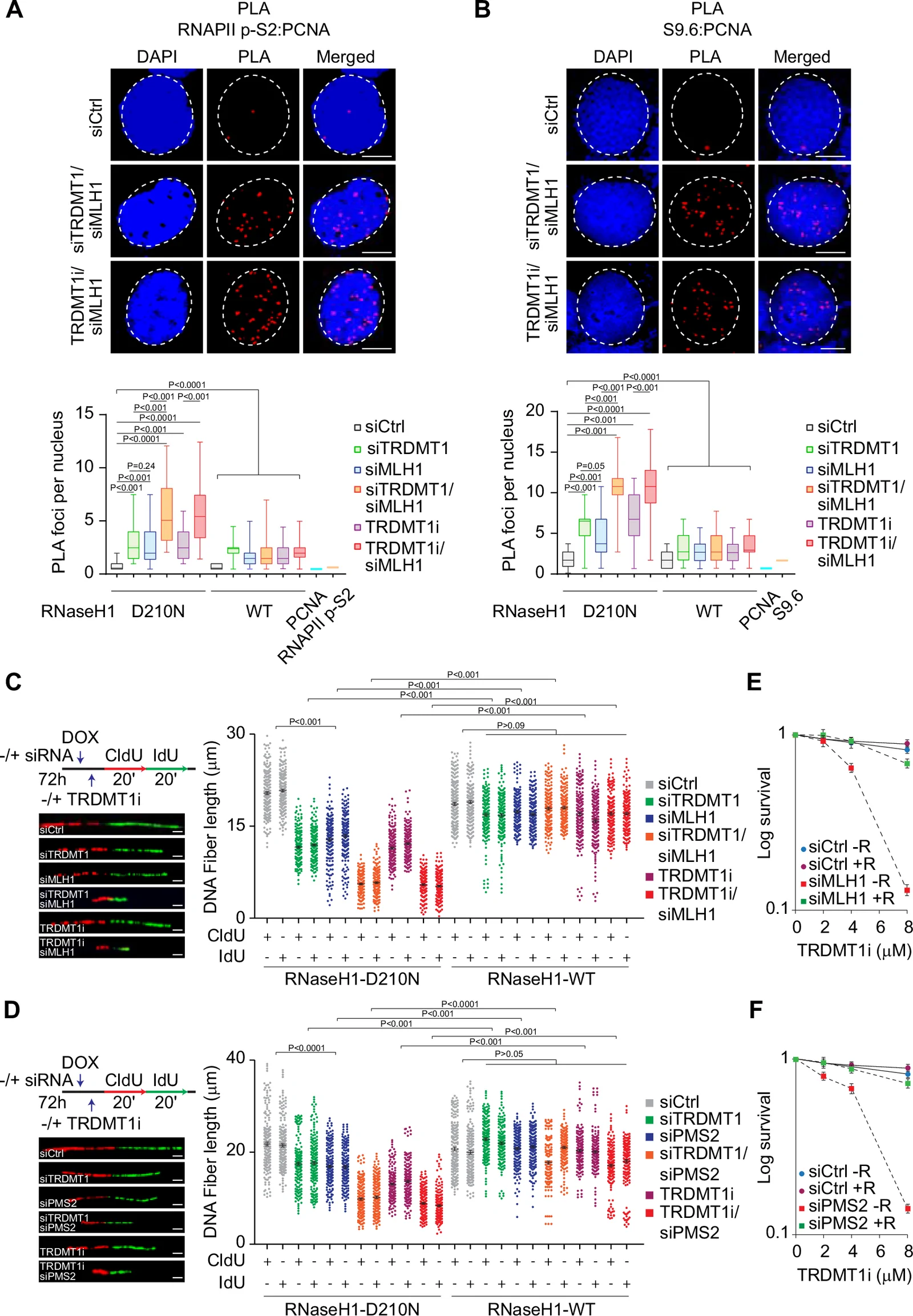
R-loops drive TRCs and replication stress upon loss of TRDMT1 and MutLα. **A** Representative images of HeLa cells analyzed by PLA using anti-RNAPII p-S2 and anti-PCNA antibodies, alone or in combination (top). Cells were transfected with the indicated siRNAs or treated with TRDMT1i. To assess the contribution of R-loops, cells were induced to express RNaseH1-WT or RNaseH1-D210N. Quantification of nuclear PLA foci is shown in box plots (bottom). *n* = 3 biological replicates, 160–170 cells per replicate. Scale bar, 5 μm. **B** HeLa cells were analyzed as in (A), except PLA was performed using anti-S9.6 and anti-PCNA antibodies (*n* = 3 biological replicates, 160–170 cells per replicate). Scale bar, 5 μm. **C**,** D** Experimental schemes, representative images, and quantification of DNA fiber length in HeLa cells transfected with the indicated siRNAs or treated with TRDMT1i. To assess the contribution of R-loops, cells were induced to express RNaseH1-WT or RNaseH1-D210N. Lengths of individual CldU- and IdU-labeled replication tracts are shown in scatter plots (*n* = 3 biological replicates, 60–70 fibers per replicate). Data are presented as mean values ± SEM. Scale bar, 10 μm. **E**,** F** TRDMT1i sensitivity of HeLa cells was assessed by colony formation assay. Cells were transfected with the indicated siRNAs or treated with TRDMT1i and induced to express RNaseH1-WT or RNaseH1-D210N to assess the contribution of R-loops. Data are presented as mean values ± SEM (*n* = 3 biological replicates). All statistical significance was determined by two-tailed unpaired Student’s *t* tests to generate *P* values. In all box plots, the center lines represent medians, the lower and upper bounds of the boxes represent the 25th and 75th percentiles, respectively, and the whiskers represent the minimum and maximum values. All numeric values are available in the Source Data.

The functions of TRDMT1 and MLH1 in suppressing R-loops and TRCs suggest that the aberrant R-loops in cells lacking TRDMT1 and MLH1 are the cause of TRCs. To test this possibility, we analyzed TRCs with the RNAPII p-Ser2:PCNA PLA in cells induced to express RNaseH1-WT or the catalytically inactive RNaseH1 mutant (RNaseH1-D210N). Expression of RNaseH1-WT but not RNaseH1-D210N is expected to remove R-loops in cells^37^. In cells expressing RNaseH1-D210N, knockdown of TRDMT1, MLH1, or both proteins, as well as TRDMT1i treatment of control or MLH1 knockdown cells, increases PLA foci (Fig. 4A). However, this induction of PLA foci is largely eliminated in cells expressing RNaseH1-WT (Fig. 4A). These results suggest that the increase of TRCs upon TRDMT1 and MLH1 loss is indeed dependent on R-loops. Similarly, increases of S9.6:PCNA PLA foci are detected in cells expressing RNaseH1-D210N, but substantially reduced by the expression of RNaseH1-WT (Fig. 4B). Together, these results suggest that R-loops are indeed responsible for the increased TRCs in TRDMT1- and MLH1-deficient cells.

To test whether R-loops are the cause of replication fork slowdown in TRDMT1- and MLH1-deficient cells, we performed DNA fiber assays in cells induced to express RNaseH1-WT or RNaseH1-D210N. In cells expressing RNaseH1-D210N, knockdown of either TRDMT1 or MLH1 alone reduces fork speed, and co-depletion of both proteins slows down forks further (Fig. 4C). Replication forks are also slowed down by TRDMT1i alone, and further impeded in MLH1 knockdown cells after TRDMT1i treatment (Fig. 4C). In RNaseH1-WT-expressing cells, however, the slowdown of replication forks in all the conditions is largely eliminated (Fig. 4C), suggesting that the replication defect caused by loss of TRDMT1 and MLH1 is dependent on R-loops. These results causally link the increase of R-loop-mediated TRCs upon loss of TRDMT1 and MLH1 to a severe defect in replication fork progression.

Given that TRDMT1i selectively induces synthetic lethality in MutLα-deficient cells, but not MutLβ and MutS-deficient cells, we next tested whether loss of PMS2 and MSH2 also induces R-loop-dependent replication defect. In cells expressing RNaseH1-D210N, depletion of PMS2 slows down replication forks and further reduces fork speed after TRDMT1 knockdown or inhibition (Fig. 4D, Supplementary Fig. 6B). These fork-slowing effects of PMS2 depletion are diminished by the expression of RNaseH1-WT, suggesting that PMS2, like MLH1, maintains replication by preventing R-loop-mediated TRCs. In contrast, although MSH2 depletion also reduces fork speed in cells expressing RNaseH1-D210N, co-depletion of TRDMT1 and MSH2, or TRDMT1i treatment of MSH2 knockdown cells does not reduce fork speed further (Supplementary Fig. 6A, C). Moreover, the fork slowdown induced by MSH2 loss is not affected by the expression of RNaseH1-WT (Supplementary Fig. 6D). Interestingly, like in cells in which TRDMT1 was depleted or inhibited, the slowdown of forks in cells lacking both TRDMT1 and MSH2 is reduced by RNaseH1-WT (Supplementary Fig. 6D), suggesting that the effect of TRDMT1 loss is dominant, such that MSH2 loss no longer affects fork speed in the absence of TRDMT1. Although how MSH2 loss slows forks remains to be elucidated, these results suggest that MSH2, a component of MutS, does not play a major role in countering R-loop-mediated TRCs. Similar to MSH2 knockdown, depletion of PMS1 also slows down replication forks in an R-loop-independent manner (Supplementary Fig. 6D, E). Thus, MutLα, but not MutS and MutLβ, prevents R-loop-associated replication defect, which may underlie the sensitivity of MutLα-deficient cells to TRDMT1i.

To investigate whether R-loop-associated replication defect is the cause of TRDMT1i-induced synthetic lethality in MutLα-deficient cells, we analyzed the effects of RNaseH1-WT expression on the sensitivity of cells to TRDMT1i. Knockdown of MLH1 or PMS2, but not MSH2 or PMS1, increases the TRDMT1i sensitivity of cells (Fig. 4E, F and Supplementary Fig. 6F, G). Importantly, expression of RNaseH1-WT reduces the sensitivity of MLH1 and PMS2 knockdown cells to TRDMT1i (Fig. 4E, F), showing that R-loops are indeed required for the TRDMT1i-induced cell death. Therefore, R-loops, which promote TRCs and fork slowdown in the MutLα-deficient cells treated with TRDMT1i, also drive TRDMT1i-induced synthetic lethality.

### Interplay between TRDMT1 and MutLα in R-loop suppression

**Fig. 5:**
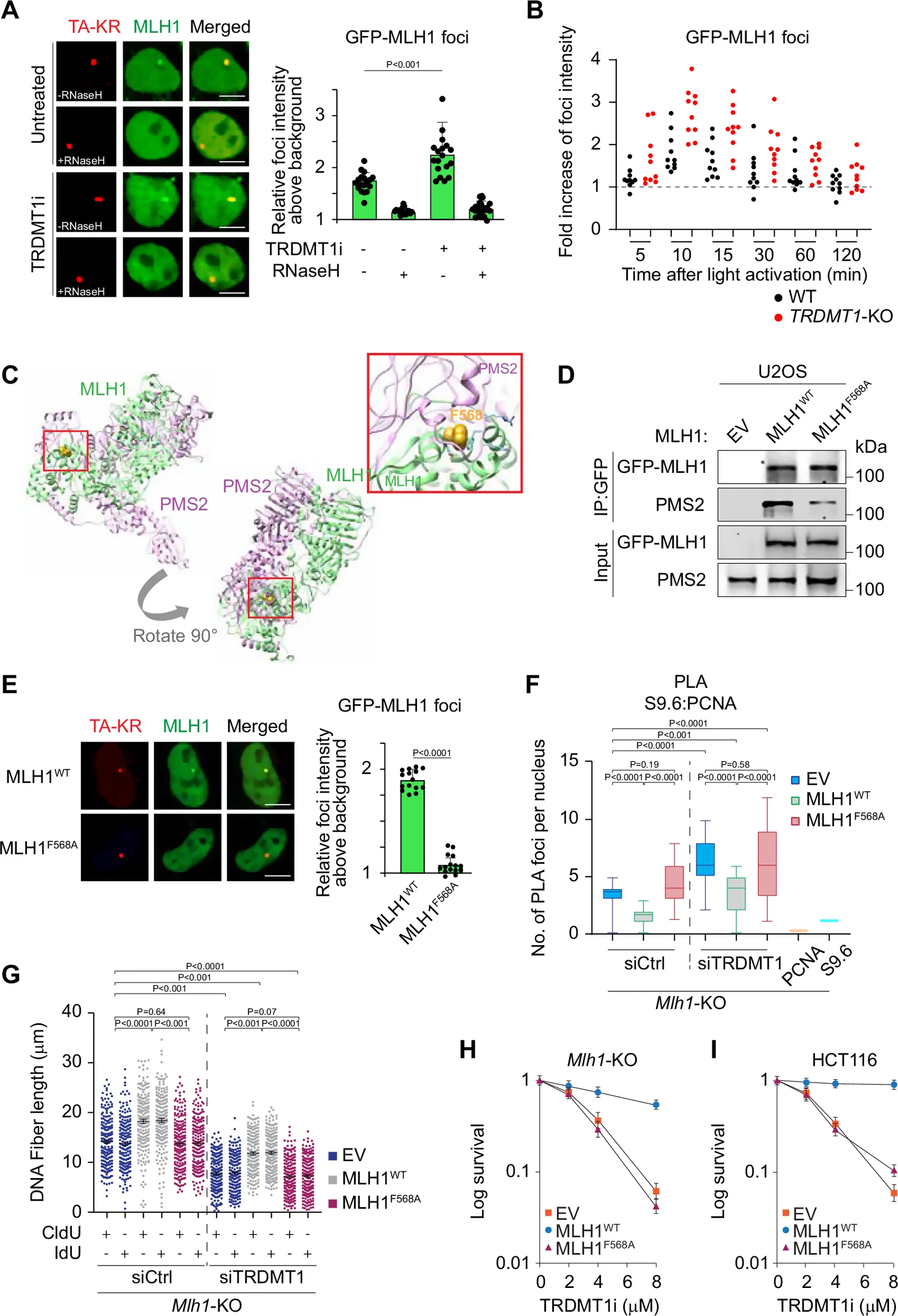
MLH1 localizes to R-loops in a PMS2-dependent manner independently of TRDMT1. **A** U2OS-TRE cells transfected with TA-KR and GFP-MLH1 expression plasmids were left untreated or treated with TRDMT1i, light-irradiated for 20 min, and allowed to recover for 10 min before microscopy. Data are presented as mean values ± SEM (*n* = 3 biological replicates). Scale bar, 10 μm. **B** Time-course analysis of GFP-MLH1 recruitment to TA-KR sites in TRDMT1-proficient and TRDMT1-deficient cells. Quantification represents fold enrichment of GFP-MLH1 intensity at TA-KR sites. *n* = 1 biological replicate. **C** Structural model of the MLH1 (green)-PMS2 (purple) complex generated using the Chai Discovery Server. MLH1 residue F568 (gold) is located at the protein-protein interface.** D** U2OS cells were transfected with GFP-MLH1^WT^, GFP-MLH1^F568A^, or GFP-empty vector (EV) plasmids. MLH1-PMS2 interaction was analyzed by co-immunoprecipitation using anti-GFP antibody. The co-immunoprecipitation experiment was performed three times, yielding consistent results. **E** U2OS-TRE cells transfected with TA-KR and GFP-MLH1^WT^ or GFP-MLH1^F568A^ plasmids were light-irradiated for 20 min and allowed to recover for 10 min before microscopy. Data are presented as mean values ± SEM. (*n* = 3 biological replicates). Scale bar, 10 μm. **F** CT26 *Mlh1-KO* cells transfected with GFP-MLH1^WT^, GFP-MLH1^F568A^, or GFP-EV plasmids were analyzed by PLA using anti-S9.6 and anti-PCNA antibodies, alone or in combination (*n* = 3 biological replicates, 160–170 cells per replicate). In the box plot, the center lines represent medians, the lower and upper bounds of the boxes represent the 25th and 75th percentiles, respectively, and the whiskers represent the minimum and maximum values. **G** CT26 *Mlh1-KO* cells transfected as in (**F**) were analyzed by DNA fiber assay (*n* = 3 biological replicates, 60–70 fibers per replicate). Data are presented as mean values ± SEM. **H** CT26 *Mlh1-KO* cells transfected as in (**F**) were analyzed for TRDMT1i sensitivity by colony formation assay (*n* = 3 biological replicates). Data are presented as mean values ± SEM. **I** HCT116 cells transfected with GFP-MLH1^WT^, GFP-MLH1^F568A^, or GFP-EV plasmids were analyzed for TRDMT1i sensitivity by colony formation assay (*n* = 3 biological replicates). Data are presented as mean values ± SEM. All statistical significance was determined by two-tailed unpaired Student’s *t* tests to generate *P* values. All numeric values are available in the Source Data.

To investigate how TRDMT1 and MutLα function in concert to suppress R-loops, we turned to the DART assay that we previously established^38^. In the DART assay, KillerRed (KR), a protein that releases ROS in a light-activated manner, is targeted to an array of tetracycline repressive elements (TRE) integrated to a single locus in the genome (Supplementary Fig. 7A). A fusion protein of KR, Tet repressor (TetR), and the transcription activator VP16 (TA-KR) marks the TRE array in cells and activates transcription locally. Upon light activation, TA-KR inflicts local DNA damage and induces R-loop formation in a transcription-dependent manner, providing a robust method to monitor the localization and functions of R-loop regulators. Using the DART assay, we previously showed that TRDMT1 colocalizes with R-loops and promotes R-loop-dependent m5C formation^13^. To understand how MLH1 suppresses R-loops, we analyzed the localization of MLH1 with the DART assay. Like TRDMT1, MLH1 is readily detected at the R-loop-enriched locus marked by TA-KR (Fig. 5A and Supplementary Fig. 7A). Consistent with the induction of R-loops by DNA damage at this locus, the colocalization of S9.6 and MLH1 foci is induced by the light activation of TA-KR (Supplementary Fig. 7A). The localization of MLH1 to the TA-KR-marked locus is abolished by RNaseH treatment of cells (Fig. 5A), confirming its R-loop dependency. Notably, the localization of MLH1 to R-loops is increased upon TRDMT1i treatment and in *TRDMT1-KO* cells (Fig. 5A, B and Supplementary Fig. 7B), supporting the idea that MLH1 partially compensates for the loss of TRDMT1 in the absence of m5C. Furthermore, the localization of MLH1 is not affected by MSH2 knockdown (Supplementary Fig. 7C), showing that MLH1 is recruited to R-loops independently of MutS.

MLH1 uses its C-terminal domain (CTD) to interact with PMS2 (Pms1 in yeast), which is important for the endonuclease activity of MutLα and the activation by PCNA^39,40^. To understand whether the interaction between MLH1 and PMS2 is important for the localization and function of MutLα, we used Chai-1, a structure prediction platform, to perform protein docking of MLH1 and PMS2 (Fig. 5C). Our analysis suggests that F568 of MLH1 is a key residue in the CTD that interacts with PMS2. To test if F568 is required for PMS2 binding, we expressed both GFP-tagged wild-type MLH1 (MLH1^WT^) and the MLH1^F568A^ mutant in cells and performed immunoprecipitation with anti-GFP antibody (Fig. 5D). MLH1^F568A^ is expressed as well as MLH1^WT^ but fails to bind endogenous PMS2 efficiently, confirming its defect in heterodimerization. In the DART assay, MLH1^F568A^ is severely impaired for the localization to the TA-KR-marked site (Fig. 5E), suggesting that the formation of MutLα complex is required for MLH1 recruitment. Consistent with the recruitment of MLH1 to R-loops, MLH1^WT^ binds to DNA–RNA hybrids efficiently in cell extracts (Supplementary Fig. 7D). In contrast, MLH1^F568A^ fails to bind DNA–RNA hybrids in cell extracts (Supplementary Fig. 7D), supporting the notion that MLH1 binds hybrids as part of MutLα. Interestingly, m5C modification of the RNA in DNA–RNA hybrids reduces MLH1^WT^ binding, providing a possible explanation of the relatively small contribution of MLH1 to the suppression of m5C-positive R-loops.

When MLH1^WT^ and MLH1^F568A^ were expressed in CT26 *Mlh1-KO* cells, MLH1^WT^ reduces the collisions between R-loops and replication forks as shown by the S9.6:PCNA PLA, but MLH1^F568A^ fails to do so (Fig. 5F and Supplementary Fig. 7E, F), suggesting that MLH1^F568A^ is defective for R-loop suppression. Even in *Mlh1-KO* cells depleted of TRDMT1, MLH1^WT^ but not MLH1^F568A^ suppresses collisions between R-loops and forks (Fig. 5F and Supplementary Fig. 7F), showing that MLH1^F568A^ is defective in a TRDMT1-independent function. Furthermore, expression of MLH1^WT^ in *Mlh1-KO* cells increases fork speed even after TRDMT1 knockdown (Fig. 5G and Supplementary Fig. 7G), suggesting that MLH1^WT^ promotes R-loop removal independently of TRDMT1. In contrast, MLH1^F568A^ fails to change fork speed (Figs. 5G and S7G), lending further support to the notion that MLH1 needs to function as part of MutLα to suppress R-loops.

To test the effect of MLH1^F568A^ on TRDMT1i sensitivity, we expressed both MLH1^WT^ and MLH1^F568A^ in *Mlh1-KO* cells and in HCT116, an MLH1-deficient human colon cancer cell line^32^. In both CT26 *Mlh1-KO* and HCT116 cells, expression of MLH1^WT^ but not MLH1^F568A^ reduces TRDMT1i sensitivity (Fig. 5H, I and Supplementary Fig. 7H), showing that a defect in the MLH1-PMS2 interaction is sufficient to render cells susceptible to TRDMT1i-induced synthetic lethality.

### Involvement of MutLα ATPase and nuclease activities and EXO1 in R-loop suppression

**Fig. 6:**
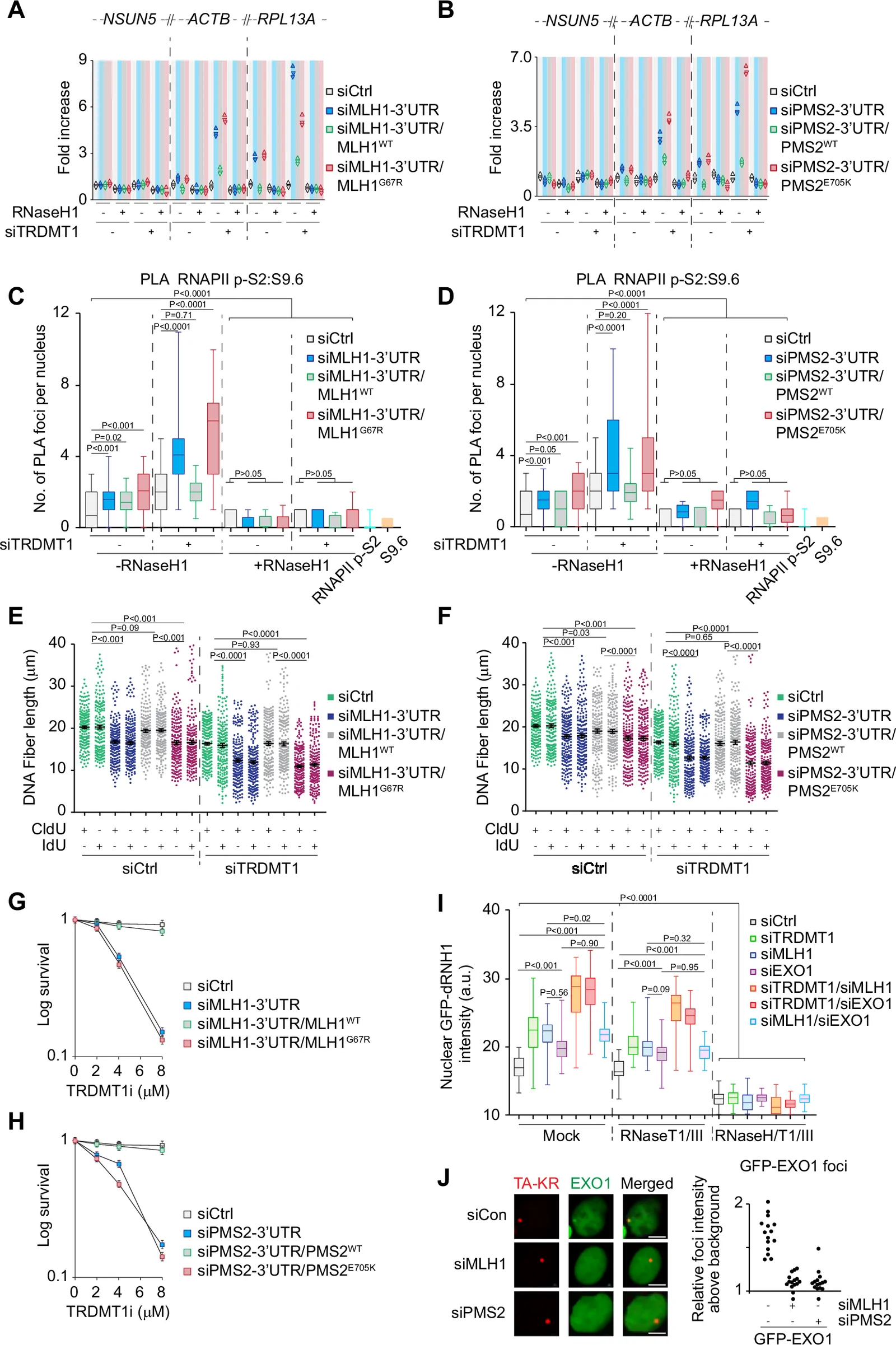
MutLα suppresses R-loops through its ATPase and nuclease activities and by recruiting EXO1. **A**,** B** DRIP-qPCR analysis of R-loop levels at the *NSUN5, ACTB*, and *RPL13A* loci in HeLa cells. Cells were transfected with siRNAs targeting MLH1-3′UTR or PMS2-3′UTR together with control or TRDMT1 siRNA. Cells depleted of endogenous MLH1 or PMS2 were complemented with MLH1^WT^ or MLH1^G67R^ (**A**), or PMS2^WT^ or PMS2^E705K^ (**B**). R-loop specificity was confirmed by RNaseH1 induction. *n* = 2 biological replicates (3 independent sets of technical replicates). Datapoints from different biological replicates are shown as symbols of different shapes. **C**,** D** Box plots showing nuclear RNAPII p-S2:S9.6 PLA foci in HeLa cells transfected with the indicated siRNAs and complemented with MLH1 or PMS2 variants as in (**A**–**B**) (*n* = 3 biological replicates, 160–170 cells per replicate). **E**,** F** DNA fiber analysis of HeLa cells transfected with the indicated siRNAs and complemented with MLH1 or PMS2 variants as in (**A**–**B**) (*n* = 3 biological replicates, 60–70 fibers per replicate). Data are presented as mean values ± SEM. **G**,** H** HeLa cells were transfected with the indicated siRNAs and complemented with MLH1 or PMS2 variants as in (**A**, **B**), and TRDMT1i sensitivity was analyzed by colony formation assay. Data are plotted as log survival (*n* = 3 biological replicates). Data are presented as mean values ± SEM. **I** Box plots showing GFP-dRNH1 R-loop staining intensity in HeLa cells transfected with the indicated siRNAs (*n* = 3 biological replicates, 20 cells per replicate). **J** U2OS-TRE cells were transfected with the indicated siRNAs and plasmids expressing TA-KR and GFP-EXO1. Cells were light-irradiated for 30 min and allowed to recover for 30 min before microscopy. *n* = 1 biological replicate. Scale bar, 10 μm. All statistical significance was determined by two-tailed unpaired Student’s *t* tests to generate *P* values. In all box plots, the center lines represent medians, the lower and upper bounds of the boxes represent the 25th and 75th percentiles, respectively, and the whiskers represent the minimum and maximum values. All numeric values are available in the Source Data.

Both the ATPase activity of MLH1 and the endonuclease activity of PMS2 are required for MMR^41,42^. During MMR, the ATP hydrolysis by MLH1 induces a conformational change in the MutLα complex, activating the endonuclease activity of PMS2. To test whether the ATPase and nuclease activities of MutLα are required for R-loop suppression, we generated the ATPase-defective MLH1^G67R^ mutant and the nuclease-defective PMS2^E705K^ mutant^41,42^. These two mutants, as well as MLH1^WT^ and PMS2^WT^, were individually expressed in cells in which endogenous MLH1 or PMS2 was knocked down by siRNA targeting the 3’ UTR (Supplementary Fig. 8A, B). In contrast to MLH1^WT^ and PMS2^WT^, MLH1^G67R^ and PMS2^E705K^ fail to suppress R-loops in MLH1/PMS2 knockdown cells treated with or without TRDMT1 siRNA (Fig. 6A, B). In addition, MLH1^G67R^ and PMS2^E705K^ are unable to suppress TRCs in MLH1/PMS2 knockdown cells treated with or without TRDMT1 siRNA as MLH1^WT^ and PMS2^WT^ do (Fig. 6C, D and Supplementary Fig. 8C, D). Finally, MLH1^G67R^ and PMS2^E705K^ do not restore fork speed in MLH1/PMS2 knockdown cells treated with or without TRDMT1 siRNA as MLH1^WT^ and PMS2^WT^ do (Fig. 6E, F and Supplementary Fig. 8E, F). Consistent with their inability in suppressing R-loops and TRCs, MLH1^G67R^ and PMS2^E705K^ do not reduce the TRDMT1i sensitivity of MLH1/PMS2 knockdown cells as MLH1^WT^ and PMS2^WT^ do (Fig. 6G, H). Together, these results demonstrate that both the ATPase and nuclease activities of MutLα are required for suppressing R-loops, TRCs, and TRDMT1i sensitivity.

During MMR, MutLα functions with EXO1 to remove mismatched nucleotides^19^. To investigate if EXO1 is involved in the suppression of R-loops, we tested the correlation between EXO1 expression and TRDMT1i sensitivity in cancer cell lines. A correlation between low EXO1 expression and high TRDMT1i sensitivity is observed (Supplementary Fig. 9A). Furthermore, cell lines highly sensitive to TRDMT1i display lower EXO1 expression than those resistant to TRDMT1i (Supplementary Fig. 9B). Thus, EXO1 deficiency is associated with increased TRDMT1i sensitivity in cancer cells. To understand if EXO1 suppresses R-loops, we performed R-loop staining with GFP-dRNH1 in cells treated with EXO1 siRNA, either alone or in combination with TRDMT1 or MLH1 siRNA (Fig. 6I and Supplementary Fig. 9C, D). Knockdown of EXO1 alone increases R-loops similarly as TRDMT1 or MLH1 knockdown. Notably, while co-depletion of TRDMT1 and EXO1 increases R-loops further compared to single knockdowns, co-depletion of MLH1 and EXO1 does not. Consistently, co-depletion of MLH1 and EXO1 does not further increase TRDMT1i sensitivity compared to single knockdowns (Supplementary Fig. 9E). These results suggest that MLH1 and EXO1 function in the same pathway to suppress R-loops. To understand how MutLα and EXO1 function together to suppress R-loops, we tested if EXO1 is recruited to R-loops by MutLα. GFP-EXO1 foci are readily detected at the DNA damage sites induced by TA-KR, but this localization is severely impaired by the loss of either MLH1 or PMS2 (Fig. 6J). Together, these results suggest that MutLα uses its ATPase and nuclease activities and recruits EXO1 to remove R-loops.

### TRDMT1i inhibits growth of MLH1-deficient tumors in vivo

**Fig. 7:**
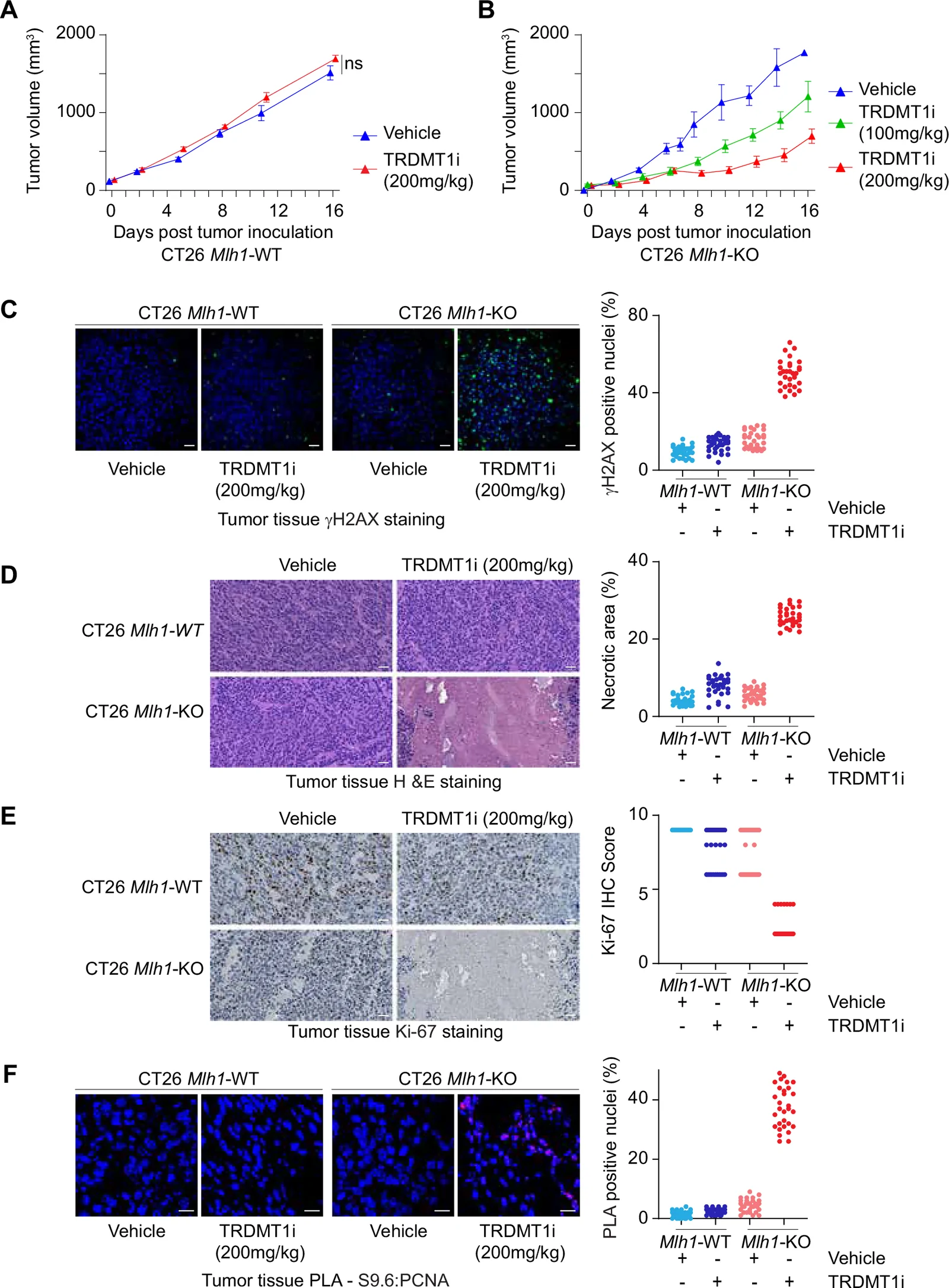
TRDMT1i suppresses growth of MLH1-deficient tumors in vivo. **A** Tumor volume measurements of BALB/c mice bearing CT26 *Mlh1-WT* tumors that were untreated or treated with TRDMT1i (200 mg/kg) for the indicated time periods (*n* = 5 mice per group). Data are presented as mean values ± SEM. **B** Tumor volume measurements of BALB/c mice bearing CT26 *Mlh1-KO* tumors that were untreated or treated with TRDMT1i (100 or 200 mg/kg) for the indicated time periods (*n* = 5 mice per group). Data are presented as mean values ± SEM. **C** Representative images of nuclear γH2AX foci detected by immunofluorescence staining in excised CT26 *Mlh1-WT* or CT26 *Mlh1-KO* tumor sections that were untreated or treated with TRDMT1i. The scatter plot shows fractions of γH2AX positive nuclei in individual sections. Thirty images of 3 independent sections of a tumor (*n* = 1) were analyzed. Scale bar, 10 μm. **D** Representative H&E-stained sections of excised CT26 *Mlh1-WT* or CT26 *Mlh1-KO* tumors that were untreated or treated with TRDMT1i. The scatter plot shows fractions of necrotic cells in individual sections. Thirty images of 3 independent sections of a tumor (*n* = 1) were analyzed. Scale bar, 50 μm. **E** Representative images of Ki-67 immunohistochemical staining in excised CT26 *Mlh1-WT* or CT26 *Mlh1-KO* tumor sections that were untreated or treated with TRDMT1i. The scatter plot shows Ki-67 immunohistochemistry (IHC) scores in individual sections. Thirty images of 3 independent sections of a tumor (*n* = 1) were analyzed. Scale bar, 50 μm. **F** Representative images of nuclear S9.6:PCNA PLA foci in CT26 *Mlh1-WT* or CT26 *Mlh1-KO* tumors that were untreated or treated with TRDMT1i. The scatter plot shows fractions of PLA-positive cells in individual sections. Thirty images of 3 independent sections of a tumor (*n* = 1) were analyzed. Scale bar, 10 μm. All statistical significance was determined by two-tailed unpaired Student’s *t* tests to generate *P* values. All numeric values are available in the Source Data.

The ability of TRDMT1i to induce synthetic lethality in MLH1-deficient cells prompted us to test whether TRDMT1i can be used to target MLH1-deficient tumors in vivo. Using CT26 *Mlh1-WT* and *Mlh1-KO* cells, we generated syngeneic mouse models of MLH1-proficient and -deficient tumors in BALB/c mice. The growth of MLH1-proficient tumors is not affected by TRDMT1i administered by oral gavage at 200 mg/kg (Fig. 7A). In contrast, the growth of *Mlh1-KO* tumors is reduced by TRDMT1i at both 100 mg/kg and 200 mg/kg in a dose-dependent manner (Fig. 7B). These results show that TRDMT1i can indeed selectively inhibit the growth of MLH1-deficient tumors in vivo.

To investigate how TRDMT1i inhibits growth of MLH1-deficient tumors in vivo, we analyzed the MLH1-proficient and -deficient tumors treated with vehicle or TRDMT1i with several assays. The levels of γH2AX staining in TRDMT1i-treated MLH1-deficient tumors are substantially higher than those in TRDMT1i-treated MLH1-proficient tumors and vehicle-treated MLH1-deficient tumors (Fig. 7C), showing that TRDMT1i preferentially induces DNA damage in MLH1-deficient tumors in vivo. Hematoxylin and Eosin (H&E) staining reveals an increase of areas of necrosis in TRDMT1i-treated MLH1-deficient tumors (Fig. 7D). Ki-67, a marker of cell proliferation, is reduced by TRDMT1i in MLH1-deficient tumors but not in MLH1-proficient tumors (Fig. 7E), suggesting that TRDMT1i induces a loss of proliferating cells specifically in MLH1-deficient tumors. To test whether TRDMT1i induces cell death by increasing R-loop-mediated TRCs, we applied the S9.6:PCNA PLA to tumor sections. PLA foci are detected in tumors only when both antibodies are used (Fig. 7F and Supplementary Fig. 10A), validating the specificity of this PLA for tumor samples. Strikingly, TRDMT1i treatment results in a drastic increase of PLA foci in MLH1-deficient tumors, but not in MLH1-proficient tumors (Fig. 7F), providing evidence that TRDMT1i increases R-loop-mediated TRCs in tumors lacking MLH1. Together, these results suggest that TRDMT1i inhibits growth of MLH1-deficient tumor in vivo by increasing R-loop-mediated TRCs, which subsequently induces a surge of replication stress, DNA damage, and ultimately cell death.

## Discussion

Our previous studies suggest that TRDMT1 is a unique RNA methyltransferase that specifically modifies the RNA in R-loops with m5C^16^. In response to DNA damage, TRDMT1 is recruited to damage-induced R-loops to generate m5C in RNA, thereby promoting DNA repair in transcribed regions^8,13,14,16^. The TRDMT1i sensitivity screen in this study presents a broader view of the cancer dependency on TRDMT1. The association of TRDMT1i sensitivity with tumor types of high genomic and chromosomal instability suggests that genomic stress is a general cause of TRDMT1 dependency, but the sources of such genomic stress may vary among and within tumor types. The screening data supports the model that HRD is a cause of TRDMT1i sensitivity, but it also reveals the existence of other unknown causes in cancers. In particular, our screen uncovers a previously unappreciated, unique, and MMR-independent function of MutLα, whose loss confers TRDMT1 dependency.

What is this function of MutLα? We show that loss of MLH1 increases R-loops and TRCs in undamaged cells and slows down replication forks. Importantly, these effects are completely suppressed by expression of RNaseH1, showing that MutLα safeguards replication through R-loop suppression. This finding suggests that MutLα-deficient cancer cells are not only unstable in microsatellites as other MMR-d cancer cells, but also uniquely under high replication stress throughout the genome due to heightened TRCs. This provides a unique opportunity to target MutLα-deficient cancer cells even when other therapies exploiting MMR-d or MSI fail. It is important to note that MMR-d does not always result in MSI in cancers^43^. Targeting the TRCs in MutLα-deficient tumors may be particularly beneficial to patients with *MLH1/PMS2* mutations but MSS tumors.

How does MutLα suppress R-loops? During MMR, MutLα is recruited to DNA mismatches and loops by MutSα/β, where its ATPase and endonuclease activities are activated^39,44,45^. The activation of MutLα endonuclease activity triggers DNA incision, which leads to ssDNA gap formation by EXO1 and subsequent gap repair by DNA polymerase δ and ligase I^46^. Our data suggests that the MutLα complex also acts as an ATPase and an endonuclease at R-loops, but its partner is not MutS. We postulate that MutLα recognizes R-loops with another partner and is stimulated by it at R-loops. Our data also suggests that MutLα recruits EXO1 to R-loops and functions with it to remove R-loops. MutLα may incise the DNA strand in DNA–RNA hybrids, enabling EXO1 to degrade the DNA strand and remove R-loops. Alternatively, MutLα may nick the displaced ssDNA strand in R-loops, allowing EXO1 to degrade this DNA strand. Degradation of the displaced ssDNA strand may increase the exposure of DNA–RNA hybrids to RNaseH1 or helicases, leading to R-loop removal. Of note, a recent study suggested that MLH1 suppresses R-loops in BRCA2-deficient cells by cleaving R-loops directly^47^. Another study suggested that MutLβ binds G-quadruplexes (G4s) and acts with the FANCJ helicase to suppress TRCs^48^. Our model is distinct from these models, and it highlights the importance of the MutLα complex in R-loop suppression and its unique functional relationship with TRDMT1.

Our data also reveals previously unknown functions of TRDMT1 in regulating R-loops and replication in undamaged cells. These functions of TRDMT1 are distinct from that in TC-HR but linked to co-transcriptional R-loops at active genes. Notably, the suppression of R-loops by TRDMT1 is mediated by m5C, suggesting that TRDMT1 is a sequence-directed suppressor of R-loops and TRCs. At DNA damage sites, TRDMT1 promotes the recruitment of the fragile X mental retardation protein FMRP, which preferentially binds m5C-modified R-loops, and TET1, an eraser of m5C interacting with FMRP^14^. Both FMRP and TET1 are required for the proper resolution of R-loops at DNA damage sites and the completion of DNA repair. At active genes in undamaged cells, TRDMT1-generated m5C may recruit similar or distinct readers to resolve or remove R-loops, thereby keeping R-loop and TRC levels below a tolerable threshold.

Although previous studies have identified numerous suppressors of R-loops, why cells need so many distinct R-loop suppressors and how these proteins function in concert in cells remains largely unknown. Our results on TRDMT1 and MLH1 provide a critical clue to the answer to this question. Loss of TRDMT1 preferentially increases R-loops in C-rich sequences where m5C occurs efficiently, whereas m5C reduces MLH1 binding to hybrids in cell extracts. Moreover, inhibition of TRDMT1 increases the localization of MLH1 to R-loops in cells, and numerous R-loops that go up upon TRDMT1 loss are further increased by co-depletion of TRDMT1 and MLH1. These results suggest that m5C controls the choice between TRDMT1- and MutLα-regulated R-loop suppressing pathways (Supplementary Fig. 10B). The more likely a sequence is modified by m5C in an R-loop, the more likely the R-loop is suppressed by TRDMT1 but not MutLα. This model not only explains how the functions of TRDMT1 and MutLα in R-loop suppression are coordinated in a sequence-directed manner, but also suggests that other R-loop suppressors may also act with sequence preferences. It is conceivable that cells rely on distinct proteins to suppress R-loops in various sequence contexts, providing cells the ability to fine-tune R-loop levels in different parts of the genome and prevent massive global increase of R-loops.

Although MMR-d and MSI have been successfully used as biomarkers for immunotherapy, durable responses were only seen in a fraction of patients^49–52^. WRN and ATR inhibitors can induce synthetic lethality in MSI or MMR-d cancer cells^28,53–60^. This work suggests that TRDMT1i can also target a large fraction of MMR mutant tumors and may stimulate immunotherapy. Although both TRDMT1i and PARPi kill HRD cancer cells, many cancer cell lines respond to TRDMT1i and PARPi differently. While PARPi kills HRD cancer cells by inducing persistent ssDNA gaps and ultimately collapsed replication forks^61,62^, TRDMT1i may kill cancer cells by inducing R-loop/TRC-mediated DSBs and inhibiting the repair of these DSBs by TC-HR. These differences make TRDMT1 inhibition a promising strategy to enhance PARPi efficacy, overcome PARPi resistance, and target tumors with repair defects other than HRD. Future studies to further develop TRDMT1i and test them in biomarker-guided trials will be critical for assessing the efficacy of TRDMT1i in cancer treatment.

## Methods

### Cell culture

The human osteosarcoma cell line U2OS, human cervical adenocarcinoma cell line HeLa and human colon carcinoma cell line HCT116 were cultured in Dulbecco’s modified Eagle’s medium (DMEM) supplemented with l-glutamine, 10% fetal bovine serum (FBS), and 1% penicillin/streptomycin (PS) at 37 °C; 5% CO_2_. The mouse colon carcinoma cell line CT26 was cultured in RPMI-1640 medium supplemented with l-glutamine, 10% fetal bovine serum (FBS) at 37 °C; 5% CO_2_. The HeLa-derived cell lines inducible for GFP-tagged nuclear RNaseH1 or its mutant derivative (D210N) expression were generated by lentiviral infection and G418 selection. All HeLa-derived cell lines were cultured in Dulbecco’s modified Eagle’s medium (DMEM) supplemented with G418 (400 μg/mL) and RNaseH1-GFP expression was typically induced by doxycycline (200 ng/ml) for 48 h.

### Cell line viability screening, dose-response analysis

Cell lines are maintained as part of the MGH Center for Molecular Therapeutics for drug sensitivity profiling^63,64^. All lines for the 300-cell line screen were tested for mycoplasma and grown in either RPMI or DMEM/F12 media with 10% fetal bovine serum. All cell lines were cultured at 37 °C with 5% CO_2_. Cells were seeded into 384-well plates into duplicate plates one day prior to drug addition. Cells were treated with vehicle (DMSO) or compound stocks dissolved in DMSO with a PerkinElmer JANUS workstation. Treatments were performed at 9 dose levels with 2-fold dilutions with a top dose of 10 mM. Following drug addition, cells were incubated for five days. Cell viability was then determined using the CellTiter-Glo assay (Promega), with luminescence measured by a PerkinElmer Envision plate reader. Viability was determined as the normalized luminescence to DMSO control for each cell line (*n* = 2 replicates). Dose-response curves were fitted to a four-parameter log-logistic model using SciPy scipy.optimize.curve_fit to estimate IC_50_, and AUC.

### Bioinformatic analyses

Tumor cell line expression data and mutation data were obtained from DepMap (version 25Q2). The MSI scores were obtained from CCLE (version 24Q4). Cell lines were classified as MSS if the MSI score was <20 and as MSI if the score was ≥20, following the criteria recommended by *MSIsensor2* (https://github.com/niu-lab/msisensor2). For TRDMT1, BRCA1 and MLH1, cell lines were classified into low- and high-expression groups using the median (50%) cutoff. For PMS2, PMS1 and MSH2, the mutant and bottom 10% expressed identified as low-group and top 10% expressed cells identified as high-group.

The SBS3 mutational signature scores were retrieved from a previously published study^65^. Cell lines were stratified into low- and high-SBS3 groups using the median (50%) cutoff value.

PARP inhibitor sensitivity data were obtained from GDSC1. Pearson correlation analysis was performed to assess the association between YW1842 and PARP inhibitor sensitivity.

Chromosomal Instability (CIN) and Aneuploidy scores were obtained from the OmicsSignatures_24Q4 dataset from DepMap. Comparisons of these metrics between TRDMT1 inhibitor-sensitive and -resistant groups were performed using a two-sided Wilcoxon rank-sum test.

### Plasmids and site directed mutagenesis

The TA-KR in pBroad3 plasmid was constructed as described previously^66^. The GFP-MLH1^WT^ construct was obtained from Prof. Akira Yasui, Tohoku University, Japan. The GFP-PMS2^WT^ and GFP-EXO1 constructs were obtained from Addgene (#215124; #215125). The GFP-MLH1^F568A^, GFP-MLH1^G67R^, and GFP-PMS2^E705K^ mutants were generated using the QuikChange II Site-Directed Mutagenesis Kit (Agilent Technologies).

### Antibodies

All antibodies used in this study, together with their sources, and working dilutions are listed in Supplementary Table 1.

### RNA interference

The siRNA transfections were done using 10 nM SMARTPool siRNAs with Lipofectamine RNAiMAX reagent for 72 h. The Horizon Discovery was used to design and order SMARTPool siRNAs. The siRNA list has been included in the key resource table.

### Plasmid and siRNA transfection

For plasmid transfection, cells were transfected using Lipofectamine 2000 (Thermo Fisher Scientific) following the manufacturer’s instructions. Standard siRNA transfections were done using 15 nM siRNA SMARTpool from Dharmacon-Horizon Discovery with Lipofectamine RNAiMAX reagent (Thermo Fisher Scientific) for 72 h at 37 °C; 5% CO_2_.

### GFP-dRNH1 expression and purification

BL21(DE3) cells transformed with pGFP-RNaseH1-D210N (Addgene #174448) were grown overnight at 37 °C in 2 × LB media till saturation and diluted 1:200 in 2× LB media the following day. Once the culture reached an OD of 0.4, protein expression was induced with 0.25 mM IPTG overnight at 16 °C. On the following day, cells were pelleted by centrifugation and lysed in lysis buffer (25 mM Tris-HCl, pH 7.5, 10% glycerol, 0.5 mM EDTA, 0.02% NP40, 1 mM beta-mercaptoethanol (BME), 500 mM KCl + protease inhibitors (PI)) and sonicated for 30 S ON, 60 S OFF for two rounds at 80% amplitude. The resulting lysate was centrifuged at 20,000 RPM for 30 min at 4 °C to pellet the insoluble fraction. The resulting supernatant was incubated with Ni-NTA resin and incubated for 1 h at 4 °C on a rotator. The mixture was added to a column and flow through was collected. The beads were washed with 100 mL wash buffer (25 mM Tris-HCl, pH 7.5, 10% glycerol, 0.5 mM EDTA, 0.02% NP40, 1 mM BME, 1 M KCl + 30 mM Imidazole) and eluted batchwise with elution buffer (25 mM Tris-HCl, pH 7.5, 10% glycerol, 0.5 mM EDTA, 0.02% NP40, 1 mM BME, 200 mM KCl + 250 mM Imidazole + PI) five times collecting 1 mL fractions with 10 min between each collection. Peak fractions were diluted to a final salt concentration of 100 mM and applied to SP Sepharose resin (Column Volume (CV)–200 μL) and flow through was collected. The resin was washed with 40 CV of SP wash buffer (25 mM Tris-HCl, pH 7.5, 10% glycerol, 0.5 mM EDTA, 0.02% NP40, 1 mM BME, 150 mM KCl) and eluted with 15 CV SP elution buffer (25 mM Tris-HCl, pH 7.5, 10% glycerol, 0.5 mM EDTA, 0.02% NP40, 1 mM BME, 600 mM KCl). The eluate was concentrated in Amicon 30 kDa filter and diluted further to attain 150 mM KCl concentration. The protein concentration was measured using BSA as a standard on an SDS-PAGE gel. The protein was aliquoted at final 15 µM concentration and flash frozen.

### DNA–RNA hybrid staining with GFP-dRNH1

Cells were rinsed twice with 1 mL of cold 1 × PBS, fixed with 4% PFA for 10 min on ice; rinsed twice with 1 mL cold 1 × PBS; permeabilized with 0.25% Triton X-100 in 1 × PBS and rinsed twice with 1 mL of cold 1 × PBS. For RNase T1 and RNase III treatment, the cells were subjected to enzymatic digestion with 2000 U of RNaseT1 and 5 U of ShortCut RNase III in low-salt buffer (50 mM Tris-HCl, pH 7.5, 75 mM KCl, 3 mM MgCl_2_, 0.1% BSA) and incubated for 3 h at 37 °C. For treatment with all three enzymes, coverslips were first incubated with 60 U of RNaseH overnight at 37 °C, washed in low-salt buffer, and subsequently incubated with RNasesT1 and III in low-salt buffer. After enzyme treatment, cells were subsequently washed briefly twice with 1 × PBS and blocked with staining buffer (3% BSA in PBS) for 30 min. Then coverslips were incubated with purified GFP-RNaseH1-D210N protein at 1:5000 dilution (200 μg/mL) in staining buffer for 2 h at 37 °C. After washing with PBST thrice for 5 min, the coverslips were mounted with Prolong Glass antifade mountant with DAPI and stored at 4 °C in the dark and imaged within 48 h.

### Proximity ligation assay

Cells were washed twice with 1 × PBS before being treated with CSK extraction buffer [0.2% Triton X-100, 20 mM HEPES-KOH (pH 7.9), 100 mM NaCl, 3 mM MgCl2, 300 mM sucrose, 1 mM EGTA] on ice for 5 min. Cells were fixed with 4% PFA on ice for 5 min, followed by ice-cold methanol treatment at −20 °C for 20 min. Subsequently, cells were permeabilized with 1 × PBS containing 0.5% Triton X-100 for 5 min and then blocked with 3% BSA in PBST at room temperature for 1 h. Afterward, cells were incubated with the indicated primary antibodies PCNA and S9.6 or RNAPII p-S2, at 4 °C overnight. After three washes with 1 × PBST, cells were incubated with anti-mouse minus and anti-rabbit plus PLA probes (PLA kit from Sigma-Aldrich) at 37 °C for 1 h. Following the manufacturer’s instructions, the PLA reaction was performed with the Duolink In Situ detection reagents (PLA kit). Lastly, cells were washed three times with wash buffer, stained with DAPI during the second wash, mounted on slides with Prolong Gold, and sealed with nail polish. Images were captured with Leica Stellaris confocal microscope (63×/1.4 oil objective) and quantified using ImageJ.

HeLa cells (0.1 million) were seeded in cover glass bottom dish in presence of G418 (400 μg/mL) in two sets. After 24 h, the cells were transfected with respective siRNAs. After 24 h post-transfection, in one set of cells, the media was supplemented with Doxycycline (200 ng/mL) for RNaseH1 expression for 48 h. The other set was maintained without any Doxycycline. After 72 h post-transfection, the cells were subjected to proximity ligation assay.

### DNA fiber assay

Cells were labeled with CldU (25 µM) for 20 min and then with IdU (250 µM) for 20 min. DNA fibers were spread as described previously^67^. Briefly, cells were suspended in 1xPBS (~200 cell/μL) and 6 μL of suspension were spotted on to a glass slide and allowed to dry. Cells were subsequently treated with 16 µL of spreading buffer (0.5% SDS, 200 mM Tris-HCl pH 7.4, 50 mM EDTA) for 3–5 min. Slides were tilted ( ~ 30°) to allow cell lysates to slowly run down the slide. The DNA on slides was air dried, fixed in methanol-acetic acid (3:1) for 10 min, and dried overnight at −20 °C. Fixed DNA was denatured with 2.5 N HCl for 1 h at room temperature (RT). The slides were neutralized in 400 mM Tris-HCl (pH 7.4) for 10 min. Then the slides were washed three times for 5 min with PBS and blocked in PBS-T (PBS + 0.1% Tween-20) containing 2% BSA for 30 min at RT in a humid chamber. The DNA fibers were incubated with rat anti-CldU (1:200) primary antibody in a humid chamber for 1 h at RT. The slides were washed three times for 5 min with PBST at RT on a rocker. Then DNA fibers were incubated with 200 μL of Alexa Fluor 594-conjugated anti-rat secondary antibody (1:200) in a humid chamber at RT for 45 min in dark. The slides were washed three times for 5 min with PBST at RT on a rocker. Again, the DNA fibers were incubated with rat anti-IdU (1:50) primary antibody in a humid chamber for 1 h at RT. The slides were washed three times for 5 min with PBST at RT on a rocker. Then DNA fibers were incubated with 200 μL of Alexa Fluor 488-conjugated anti-mouse secondary antibody (1:100) in a humid chamber at RT for 45 min in dark. The slides were washed three times for 5 min with PBST at RT on a rocker. The slides were air-dried and mounted with coverslips using Prolong Gold anti-fade reagent. DNA fibers were imaged at 63X with a Zeiss Axio Imager Z2 upright microscope.

### Cell survival assay

Approximately 500 cells were seeded in each 6-cm dish and cultured as described before ^17^. They were treated with TRDMT1i at indicated concentrations post seeding. After 15 days, colonies were fixed and stained with 0.3% crystal violet in methanol, and the number of colonies was counted manually.

### Apoptosis assay

Apoptosis was measured using an Annexin V-FITC/propidium iodide apoptosis detection kit according to the manufacturer’s protocol, with minor modifications. Briefly, cells were treated as indicated, harvested, and collected together with floating cells from the culture medium to avoid loss of apoptotic or dead cells. Cells were pelleted by centrifugation at 1000 × *g* for 5 min. Cell pellets were washed with PBS, counted, and 2 × 10^5^ cells per sample were resuspended in 195 μl Annexin V binding buffer. Cells were then stained with 5 μl Annexin V-FITC and 10 μl propidium iodide, gently mixed, and incubated for 10–20 min at room temperature in the dark. After staining, cells were washed or directly resuspended in PBS or Annexin V binding buffer and kept on ice protected from light until analysis. Samples were analyzed by flow cytometry within 1 h of staining. Unstained, Annexin V-FITC single-stained and propidium iodide single-stained controls were included for compensation and gating. Viable cells were defined as Annexin V⁻/PI⁻, early apoptotic cells as Annexin V⁺/PI⁻, and late apoptotic or necrotic cells as Annexin V⁺/PI⁺.

### Western blots

Cells were harvested and lysed in RIPA or IP lysis buffer supplemented with 1x protease inhibitors. Protein concentrations were normalized using BCA assay and mixed 1:1 with 2X SDS PAGE loading buffer (100 mM Tris at pH 6.8, 12% glycerol, 3.5% SDS, 0.2 M DTT). Samples were boiled for 10 min at 95 °C, subjected to electrophoresis in 10% SDS polyacrylamide gels at 80 V for 90 min. Subsequently, proteins were transferred onto PVDF membranes using a Bio-Rad electrophoretic blotting liquid transfer system for 1 h at 400 mA. Membranes were then blocked in Phosphate-buffered saline with 0.1% Tween-20 (PBS-T) and 3% BSA for 60 min at RT. Membranes were then immunoblotted with respective primary antibodies, diluted in blocking buffer, at 4 °C overnight with mild shaking. Membranes were washed twice for 5 min with PBST and incubated for 2 h at RT with respective secondary antibodies conjugated to horseradish peroxidase (HRP). Membranes were washed twice for 10 min with PBST, and an enhanced chemiluminescence solution was applied. Signals were detected using a Bio-Rad ChemiDoc MP imaging system.

### Coimmunoprecipitation

The lysates of U2OS cells transfected with Empty Vector, GFP-MLH1^WT^ or GFP-MLH1^F568A^, using Lipofectamine 2000 (Invitrogen) were prepared. Lysates were cleared by centrifugation at 13,000 rpm for 30 min and incubated with G-Sepharose protein beads attached with and without corresponding antibody overnight at 4 °C on a rocking platform. Beads were then collected by centrifugation at 8200 rpm for 30 s at 4 °C, extensively washed in lysis buffer, and resuspended in SDS gel loading buffer. The proteins were separated on a 10% SDS–polyacrylamide gel, transferred to a PVDF membrane, and analyzed by immunoblotting with the corresponding antibodies.

### Chromatin fractionation

To capture R-loop-containing chromatin fragments, S9.6 immunoprecipitation (IP) was performed in chromatin fractions. Cells were harvested by trypsinization and pelleted by centrifugation. Cell pellets were resuspended in 50 μl of ice-cold buffer A containing 10 mM HEPES, pH 7.9, 10 mM KCl, 1.5 mM MgCl₂, 0.34 M sucrose, 10% glycerol, 1 mM DTT and protease inhibitors. Triton X-100 was added to a final concentration of 0.1%, and cells were incubated on ice for 5 min. The cytoplasmic fraction was separated from nuclei by centrifugation at 1300 × *g* for 4 min at 4 °C. The nuclear pellet was washed once with 100 μl of buffer A and centrifuged again at 1300 × *g* for 4 min. Nuclei were then lysed in 50 μl of ice-cold buffer B containing 3 mM EDTA, 0.2 mM EGTA, 1 mM DTT and protease inhibitors. After incubation on ice for 10 min, soluble nuclear proteins were separated from the chromatin fraction by centrifugation at 1700 × *g* for 4 min at 4 °C. The chromatin pellet was washed once with 100 μl of buffer B and collected by centrifugation at 10,000 × *g* for 1 min. The final chromatin pellet was resuspended in 50 μl of buffer A and sheared by sonication for 5 min. Samples were subjected to IP or mixed with 6 × SDS loading dye and analyzed by immunoblotting.

### DNA–RNA hybrid pulldown

HeLa cells (0.5 million) were transfected with either GFP-MLH1^WT^ or GFP-MLH1^F568A^ construct. 18 h post-transfection, cells were lysed with IP-lysis buffer. Lysates were cleared by centrifugation at 13,000 rpm for 30 min and incubated with G-Sepharose protein beads attached with and without corresponding antibody overnight at 4 °C on a rocking platform. Beads were then collected by centrifugation at 8200 rpm for 30 s at 4 °C, and suspended in elution buffer [0.1 M Glycine (pH 2.2)] and neutralized by neutralization buffer [50 mM Tris-HCl (pH 8.0)]. A fraction of eluted protein was separated as input. Then 3′-biotin labeled RNA oligonucleotides with or without m5C and complementary ssDNA (synthesized from IDT) were annealed in a 1:1 ratio to generate the biotin labeled DNA–RNA hybrid or m5C DNA–RNA hybrid. Next, the biotin labelled hybrids were crosslinked with Dynabeads M-280 streptavidin beads. The crosslinked DNA–RNA hybrids were incubated with GFP-MLH1^WT^ or GFP-MLH1^F568A^ pulldown fractions overnight at 4 °C. After overnight incubation the beads-hybrid-protein conjugate was heated in SDS loading buffer at 95 °C for 5 min and subjected to 10% SDS-PAGE.

Oligos used for pulldown:

1. ssDNA: ATCATCACCATAACGTCGATGTATCAACTTCGATTAGTCACACCAATTAA

2. ssDNA: TTAATTGGTGTGACTAATCGAAGTTGATACATCGACGTTATGGTGATGAT

3. ssRNA: UUAAUUGGUGUGACUAAUCGAAGUUGAUACAUCGACGUUAUGGUGAUGAU

4. m5C-RNA*: UUAAUUGGUGUGACUAAUCGAAGUUGAUACAUCGACGUUAUGGUGAUGAU (* Oligo 4 is methylated at each C).

### DRIP-qPCR

The DRIP assay was performed as described previously^16^. HeLa cells (0.1 million) were seeded in cover glass bottom dish in presence of G418 (400 μg/mL) in two sets. After 24 h, the cells were transfected with respective siRNAs. After 24 h post-transfection, in one set of cells, the media was supplemented with Doxycycline (200 ng/mL) for RNaseH1 expression for 48 h. The other set was maintained without any Doxycycline. After 72 h post-transfection, the cells were trypsinized and resuspended in 1.6 mL of TE with 0.5% of SDS and 5 μl of Proteinase K (Roche Life Sciences), and incubated overnight at 37 °C. The genomic DNA was extracted with phenol/chloroform in MaXtract High Density phase lock gel tubes (Qiagen), and precipitated with ethanol/sodium acetate. After washing 5 times with 70% ethanol, the genomic DNA was resuspended in TE. DNA was digested with HindIII, BsrGI, XbaI, EcoRI, and SspI at 37 °C overnight and was purified by phenol/chloroform and ethanol method. Then, 4.4 μg DNA was incubated with 10 μg of S9.6 antibody in 1 × DRIP binding buffer (10 mM NaPO_4_ pH 7.0, 140 mM NaCl, 0.05% Triton X-100) overnight at 4 °C and washed protein G agarose beads were added for an additional 2 h. After washing three times in 1 × DRIP binding buffer, the bound immunocomplexes were eluted in elution buffer (50 mM Tris-HCl pH 8.0, 10 mM EDTA, 0.5% SDS, Proteinase K) at 55 °C with rotation. The eluted samples were then subjected to nucleic acid purification using the phenol/chloroform and ethanol method. The immunoprecipitated nucleic acid and input DNA were analyzed by qPCR using forward (F) and reverse (R) primers.

Primer list for DRIP-qPCR:

CtrlF (*NSUN5*): AAAGCATCAGCACCAGAACGC,

CtrlR (*NSUN5*): CGAGGAAGGGACCCAATAACC;

m5C-RF (*EEF2*): GTGCCATCACTCAACCATAACA,

m5C-RR (*EEF2*): TTCCTGCCTGCTAGAAATCATC;

m5C-RF (*ILF2*): TCGACAGGTGGGATCCTATAA;

m5C-RR (*ILF2*): ATCCTGTGCTCTTAGGCTTTC;

ActinF: CGGGGTCTTTGTCTGAGC;

ActinR: CAGTTAGCGCCCAAAGGAC;

RPL13AF: AGGTGCCTTGCTCACAGAGT;

RPL13AR: GGTTGCATTGCCCTCATTAC.

### R-loop mapping by CUT&TAG

The protocol for Cleavage Under Targets and Tagmentation was used with slight modifications^68^. Briefly, HeLa cells (0.5 million) were seeded in a six-well plate in presence of G418 (400 μg/mL) in two sets. After 24 h, the cells were transfected with respective siRNAs. After 24 h post-transfection, in one set of cells, the media was supplemented with Doxycycline (200 ng/mL) for RNaseH1 expression for 48 h. The other set was maintained without any Doxycycline. After 72 h post-transfection, the cells were washed twice with wash buffer [20 mM HEPES-KOH (pH 7.5), 150 mM NaCl, 0.5 mM spermidine, and 1 × protease inhibitors] and harvested with accutase solution followed by resuspension in wash buffer. Then 10 mL of concanavalin A–coated magnetic beads were activated and added to the cells with incubation at room temperature for 10 min. The supernatant was discarded using magnetic separator, and bead-bound cells were resuspended in 50 mL of antibody buffer [20 mM HEPES-KOH (pH 7.5), 150 mM NaCl, 0.5 mM spermidine, 1 × protease inhibitors, 0.05% digitonin, 0.01% NP-40, and 2 mM EDTA]. Then 20 μg of S9.6 (anti-DNA–RNA hybrid antibody) was added to incubate with the bead-bound cells by rotating overnight at 4 °C. After overnight incubation with S9.6 antibody, the samples were incubated with rabbit anti-mouse immunoglobulin G (IgG) antibody (10 μg/mL) for 1 h. The bead-bound cells were briefly washed three times with 150 μl dig-wash buffer to remove the unbound antibodies. Then samples were again incubated with mouse anti-rabbit IgG antibody (10 μg/mL) for 1 h followed by washing with 150 μl of dig-wash buffer for three times.

Next, the pA-Tn5 adapter complex ( ~ 20 mM) at 1:100 dilution prepared in dig-300 buffer [20 mM HEPES-KOH (pH 7.5), 300 mM NaCl, 0.5 mM spermidine, 1 × protease inhibitors, and 0.05% digitonin] was mixed with the bead-bound cells and was rotated at room temperature for 1 h. Then the samples were washed three times in 100 μl of dig-300 buffer to remove unbound pA-Tn5 protein. Next, cells were resuspended in 47 μl of tagmentation buffer [10 mM TAPS-NaOH (pH 8.5), 10 mM MgCl_2_, 10% DMF, and 0.85 mM ATP] and incubated at 37 °C for 1 h. The tagmentation reaction was stopped with 1.5 μl of 0.5 M EDTA, 0.5 mL of 10% SDS, and 1.0 μl of proteinase K (20 mg/mL) with further incubation at 55 °C for 1 h. The tagmentation products were purified with AMPure XP Beads and eluted in 21 μl of 0.1% Tween 20. The eluent was mixed with 0.5 μL 10 mM dNTPs, 0.4 U Q5 high-fidelity DNA polymerase, and 10 U Bst 2.0 WarmStart DNA polymerase in 20 μL of 1 × Q5 polymerase reaction buffer with 1 × Q5 high GC enhancer. The mixture was incubated at 65 °C for 30 min to complete the strand displacement reaction. Then the reaction was stopped by incubation at 80 °C for 20 min. Then, sequencing libraries were generated, the mixture was mixed with a universal i5 primer and a uniquely barcoded i7 primer and then amplified with the Q5 high-fidelity master mix. The libraries were size-selected with AMPure XP Beads and eluted in 22 μl water. The library size distribution and concentration were determined by capillary electrophoresis using an Agilent Tape station 4150 and subjected to Illumina sequencing.

### CUT&TAG data analysis

Adapter trimming was performed using Trim Galore (v0.6.10) to remove Illumina adapter sequences from raw FASTQ files. The resulting clean reads were aligned to the human reference genome (GRCh38) using Bowtie2 (v2.5.4) with the parameters–end-to-end–very-sensitive–no-mixed–no-discordant–phred33 -I 10 -X 700. PCR duplicates were removed using the markdup function in SAMtools (v1.20). BigWig files were generated using bamCoverage (v3.5.5), and peak calling was conducted with MACS2 (v2.2.7.1). Read counts were normalized as RPKM for each peak in each sample using the dba.count function in the DiffBind package. Average signal profiles were visualized using the ggChIPvis package.

For differential peak analysis, peaks with a log₂(fold change) >0.5 between any two compared groups were defined as upregulated. For the overlap analysis between R-loop peaks upregulated in siTRDMT1 or siMLH1 and m5C sites, m5C data were obtained from previously published datasets (PRJNA984695). Only m5C sites detected in U2OS-WT cells were included in the analysis. Peaks showing log₂(fold change) >0 relative to siCTRL were extended by 5 kb upstream and downstream from their loci and intersected with the m5C sites. The overlapping ratio was calculated as the number of overlapping m5C sites divided by the total number of m5C sites.

### DART assay

The TRE array with a transcription cassette was integrated at a single chromosomal locus of U2OS-TRE cells. Killer-Red (KR), which releases reactive oxygen species (ROS) upon exposure to 550–590 nm light, was fused with a transcription activator (TA). TA-KR was transfected into U2OS-TRE cells. Activation of KR was performed by exposing cells to a 15 W Sylvania cool white fluorescent bulb for 25 min using a UVP (Upland, CA) stage system. Cells were then allowed to recover for 30 min before fixation. Imaging was performed on the Olympus FV1000 confocal microscopy system (Cat. F10PRDMYR-1, Olympus), and FV1000 software was used for image acquisition. The frequency of foci-positive cells was counted from three groups of individual cells per experiment in three independent experiments. The mean intensity was calculated by dividing the measured intensity of the selected area colocalizing with KR foci by that of three arbitrarily selected nuclear regions of the same size, using ImageJ 1.52i software. The relative fold change was calculated by subtracting the background mean intensity from the measured value and dividing by the average background intensity.

For S9.6 and MLH1 co-staining, the fixed samples were steamed in TE buffer (10 mM Tris-HCl, 2 mM EDTA, pH 9.0) on the 95 °C heating block for 20 min. Then GFP and KillerRed fluorescence were quenched by treatment with 2 mM HCl for 2 min, followed by immediate washing with PBS. Complete signal quenching was confirmed under a confocal microscope. Samples were then blocked with 2% BSA in PBS for 1 h and incubated with primary antibodies overnight at 4 °C. After washing three times with 0.05% PBST, cells were incubated with the corresponding secondary antibodies including Alexa Fluor 488/594/647 goat/donkey anti-mouse/rabbit/goat IgG conjugate (1: 10,000). DAPI was added during the second washing step after staining.

### Computational modeling of the MLH1–PMS2 complex

The complex structure of human MLH1 and PMS2 was predicted using the *Chai-1 Discovery server* (https://chai1.ai), an advanced AI-based platform for macromolecular structure and interaction modeling. Full-length amino acid sequences of MLH1 (UniProt ID: P40692) and PMS2 (UniProt ID: P54278) were submitted as input. The prediction mode was set to *Protein–Protein Complex Prediction*, which leverages large-scale machine learning models trained on experimentally determined multimeric structures to generate high-confidence interaction assemblies. Five independent models were generated, each with corresponding interface, confidence scores and predicted alignment error (PAE) maps.

Among the generated models, the top-ranked prediction was selected based on the interface probability score and overall predicted local distance difference test (pLDDT) values. Structural validation was conducted by assessing inter-protein contact maps and comparing conserved domains with previously reported MLH1–PMS2 interaction regions. The final model displayed a high-confidence interface between the MLH1 CTD and PMS2 CTD, consistent with prior biochemical evidence.

Visualization and analysis of the predicted complex were carried out using UCSF Chimera, enabling detailed mapping of the interface residues, including the functionally relevant residue F568 of MLH1 that mediates PMS2 binding.

### Mouse handling

CT26 *Mlh1-WT* or *Mlh1-KO* (6.0 × 10^5^) cells were injected intramuscularly into female BALB/c mice, which were then randomly assigned into one of four groups (eight animals per condition). When mice had palpable tumors (~1 week post-injection), the TRDMT1i (100 mg/kg or 200 mg/kg) and/or saline was injected intraperitoneally once every other day for 16–20 days. Mice were then killed, and the tumors were harvested. Tumors were fixed, embedded in paraffin, and sectioned into 4 μm-thick slices. After deparaffinization and rehydration, sections were either processed for Hematoxylin and Eosin (H&E) staining or blocked and incubated for Ki-67 staining followed by detection from the BioRepository & Precision Pathology Center (BRPC), Duke University School of Medicine. All animal experiments were approved by and conducted in accordance with the guidelines established by the Institutional Animal Care and Use Committee at Duke University. Ambient temperature was controlled at 18–26 °C, relative humidity was maintained at 40–70%, with a 12 h light/12 h dark cycle.

### Immunostaining and PLA of tumor samples

The tumor sections were heated in an oven at 65 °C for 1 h. Then sections were deparaffinized and rehydrated using the following series of washes: three Xylene washes (5 min each), followed by two 100% ethanol rinses (5 min each), followed by two 95% ethanol rinses (5 min each), two 70% ethanol rinses (5 min each), two 50% ethanol rinses (5 min each), followed by two rinses with deionized H_2_O (5 min each). Next, sections were dipped in citrate acetate buffer at 95 °C for 20 min for antigen retrieval. Then sections were allowed to cool down by dipping in deionized H_2_O for 30 min. Next for staining the sections were rinsed with PBS + Triton X-100 (0.1%) for 10 min on a shaker. Then, sections were blocked with 5 % BSA in PBS + Triton X-100 (0.1%) for 1 h at RT. For PLA staining, the sections were incubated with primary antibodies PCNA and S9.6 (diluted in the blocking solution) at 4 °C overnight in the humidified chamber followed by general PLA protocol. For γH2AX staining, sections were incubated with the γH2AX antibody (diluted in the blocking solution) at 4 °C overnight in the humidified chamber. The following day, the sections were washed twice with 1% BSA in PBS + Triton X-100 (0.1%) for 10 min on a shaker and incubated with Alexa Fluor 488-conjugated anti-mouse secondary antibody (diluted in the blocking solution) at RT for 2 h in the humidified chamber. Next, the sections were washed twice with 1% BSA in PBS + Triton X-100 (0.1%) for 10 min on a shaker. The sections were partially air-dried and mounted with coverslips using Prolong Gold anti-fade reagent. The sections were imaged with Leica Stellaris confocal microscope (63X/1.4 oil objective) and quantified using ImageJ.

### Statistics and reproducibility

Statistical analyses were performed using GraphPad Prism and R software. Data are presented as mean ± SEM, median with IQR, or otherwise as indicated in the corresponding figure legends. For comparisons between two groups, statistical significance was determined using an unpaired two-tailed Student’s *t* test unless otherwise stated. Where indicated, statistical significance was assessed using a two-sided Wilcoxon rank-sum test. *P* values less than 0.05 were considered statistically significant. For box plots, the centre line represents the median, the lower and upper bounds of the box represent the 25th and 75th percentiles, respectively, and the whiskers extend to the most extreme observations within 1.5 × the IQR. Observations beyond the whiskers are displayed as individual outliers. All experiments were performed with at least three independent biological replicates unless otherwise stated. Immunoblotting and co-immunoprecipitation experiments were performed independently three times with consistent results, and representative blots are shown. Microscopy-based quantifications, including PLA, immunofluorescence, DRIP-qPCR-associated validation, γH2AX staining, Ki-67 staining, and H&E necrotic-area analysis, were performed from independent biological samples as indicated in the corresponding figure legends. For animal tumor analyses, each tumor or mouse was considered an independent biological replicate. No data were excluded from the analysis unless exclusion criteria are explicitly stated in the relevant figure legend or “Methods” section. Source data underlying the graphs, including raw numerical values used for plotting, are provided in the Source Data file.

### Reporting summary

Further information on research design is available in the Nature Portfolio Reporting Summary linked to this article.

## Supplementary information

Supplementary Information

Reporting Summary

Transparent Peer Review file

## Source data

Source data

## Data Availability

The R-loop CUT&TAG data generated in this study have been deposited in NCBI’s Sequence Read Archive (SRA) under BioProject accession number PRJNA1478282. The m5C sequencing data used in this study were obtained from a previously published dataset available in the NCBI’s SRA under BioProject accession number PRJNA984695. The IC50 and AUC data for human cancer cell lines treated with the TRDMT1 inhibitor YW1842 are available in *Zenodo* (https://zenodo.org/records/21142540)^69^. All other datasets generated in this study are provided in the main manuscript and/or its Supplementary Information files. Source data are provided with this paper.
