# Supplementary material for: Suppression of transcription-replication conflicts by sequence-coordinated actions of TRDMT1 and MutLα
Source: Nat Commun. 2026 Aug 10;17:9566. doi: 10.1038/s41467-026-76137-8 (PMC13550614; doi:10.1038/s41467-026-76137-8)
Supplement: Supplementary file 1 [file 41467_2026_76137_MOESM1_ESM.pdf]

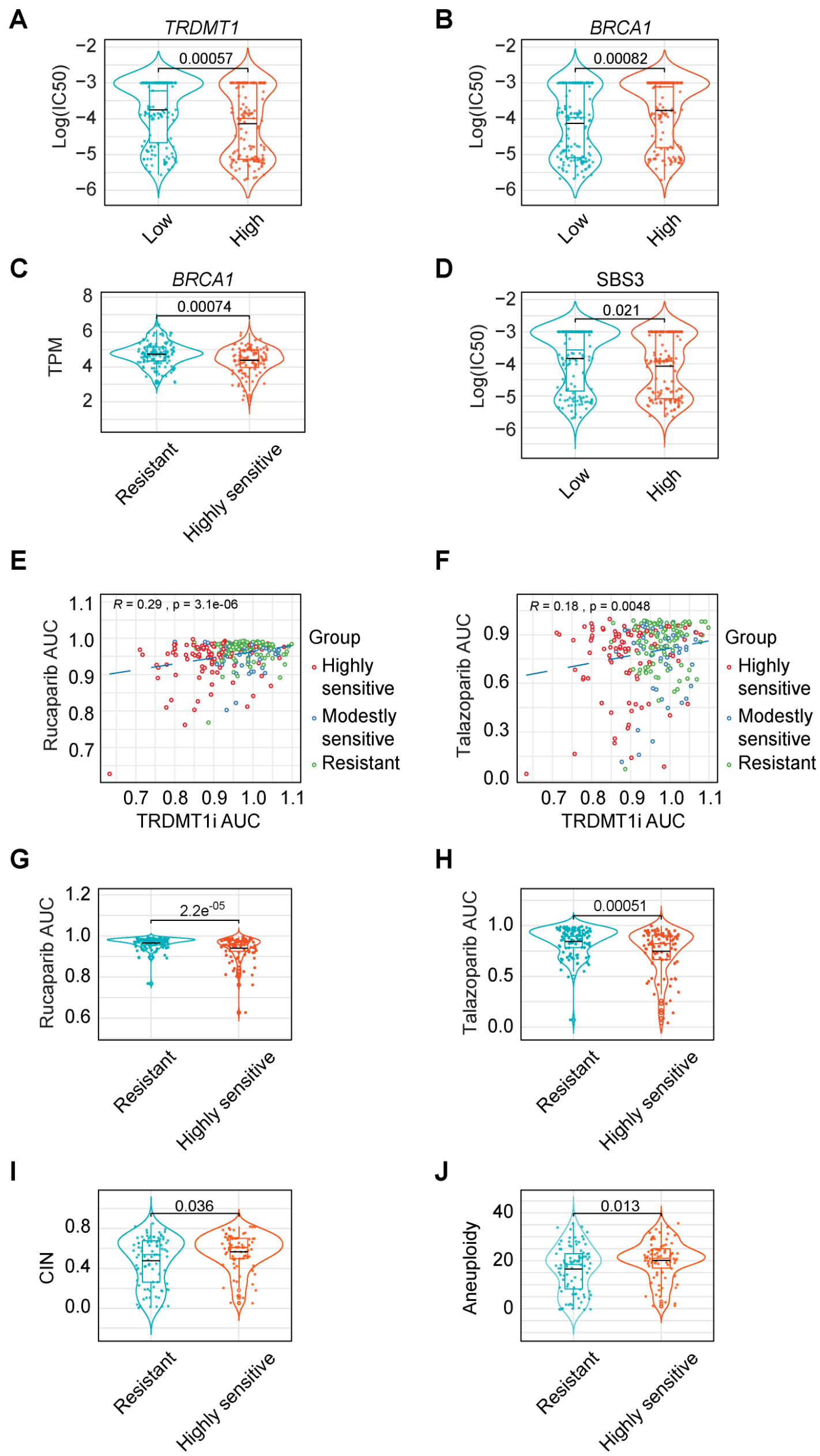

Supplementary Fig. 1. Molecular correlates and drug response associations of TRDMT1i sensitivity. (A–B) Violin plots showing differences in TRDMT1i sensitivity between cancer cell lines with high or low expression of TRDMT1 (A) and BRCA1 (B). Cell lines in the bottom 50% of expression were defined as low-expression, and those in the top 50% as high-expression groups. **(C)** Violin plot comparing BRCA1 expression between TRDMT1i-highly-sensitive and -resistant cancer cell lines. **(D)** Violin plot comparing SBS3 mutational signature levels between TRDMT1i-highly-sensitive and -resistant cancer cell lines. **(E–F)** Scatter plots showing correlations between TRDMT1i sensitivity and sensitivity to the PARP inhibitors Rucaparib (E) and Talazoparib (F). Pearson correlation analysis was used to assess associations between TRDMT1i and PARPi sensitivity. **(G–H)** Violin plots comparing sensitivity to Rucaparib (G) and Talazoparib (H) among TRDMT1i-highly-sensitive, -modestly-sensitive, and -resistant cancer cell lines. **(I–J)** Violin plots comparing CIN scores (I) and aneuploidy scores (J) between TRDMT1i-highly-sensitive and -resistant cancer cell lines. In all violin plots, each dot represents one cell line. Violin widths represent the kernel density distribution of the data. Box plots show the median (centre line), the first and third quartiles (box limits), and whiskers extending to the most extreme observations within 1.5 × the interquartile range (IQR). The black horizontal line indicates the mean. Statistical significance was assessed using a two-sided Wilcoxon rank-sum test. Exact P values are shown.

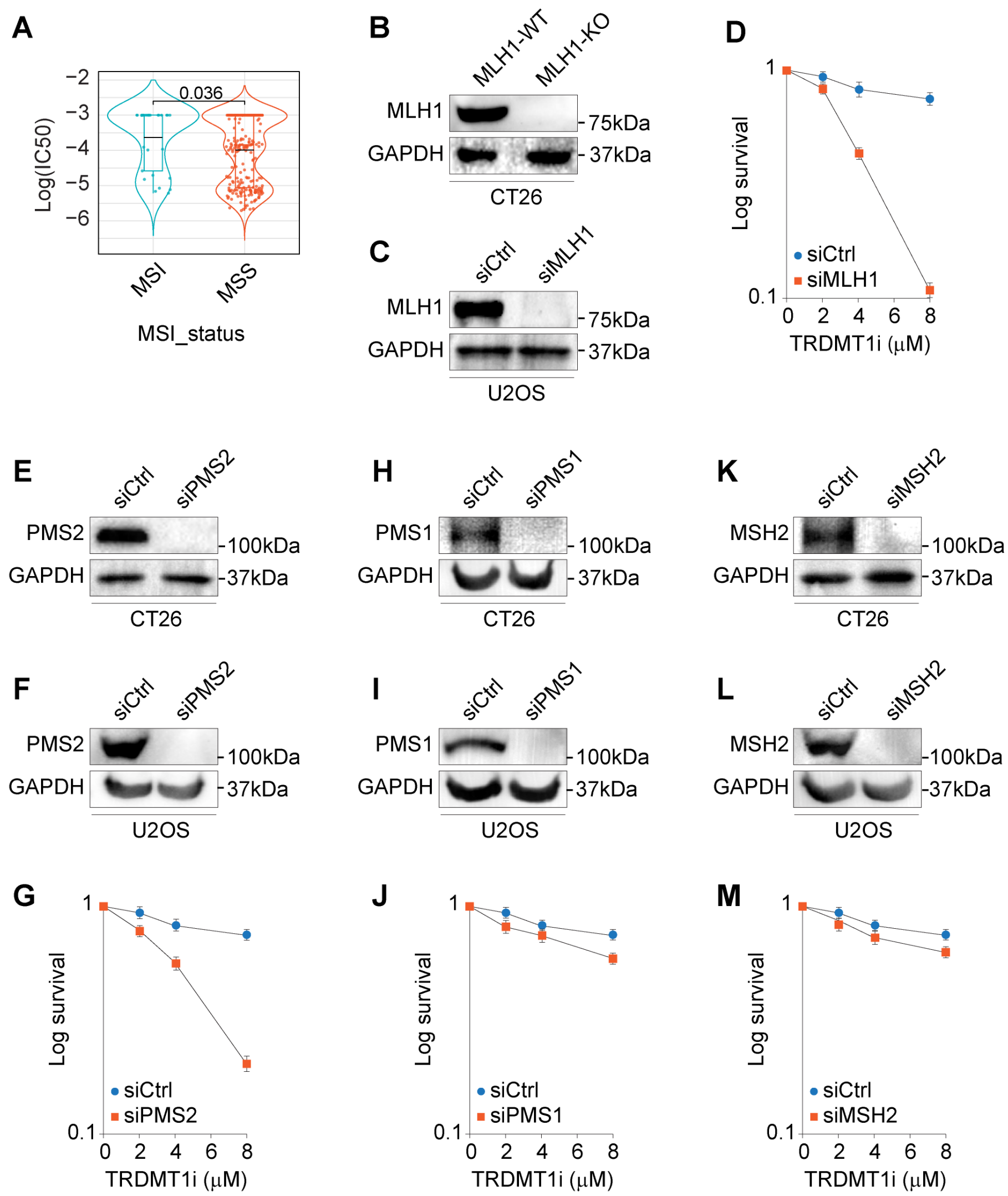

Supplementary Fig. 2. Effects of MMR gene deficiencies on TRDMT1i sensitivity. (A) Violin plot comparing TRDMT1i sensitivity between MSI and MSS cell lines. Each dot in violin plot represents one cell line. Violin widths represent the kernel density distribution of the data. Box plots show the median (centre line), the first and third quartiles (box limits), and whiskers extending to the most extreme observations within 1.5 × the interquartile range (IQR). The black horizontal line indicates the mean. Statistical significance was assessed using a two-sided Wilcoxon rank-sum test. Exact P values are shown. **(B–C)** Western blot analysis of lysates from CT26 *Mlh1-WT* and *Mlh1-KO* cells (B) or siCtrl and siMLH1 treated U2OS cells (C), probed with anti-MLH1 and anti-GAPDH antibodies. GAPDH served as a loading control. **(D)** Colony formation assay of siCtrl and siMLH1 treated U2OS cells exposed to the indicated doses of TRDMT1i (n = 3 biological replicates). Data are presented as mean values ± SEM. **(E–F)** Western blot analysis of lysates from siCtrl and siPMS2 treated CT26 cells (E) or U2OS cells (F), probed with anti-PMS2 and anti-GAPDH antibodies. GAPDH served as a loading control. **(G)** Colony formation assay of siCtrl and siPMS2 treated U2OS cells exposed to the indicated doses of TRDMT1i (n = 3 biological replicates). Data are presented as mean values ± SEM. **(H–I)** Western blot analysis of lysates from siCtrl and siPMS1 treated CT26 cells (H) or U2OS cells (I), probed with anti-PMS1 and anti-GAPDH antibodies. GAPDH served as a loading control. **(J)** Colony formation assay of siCtrl and siPMS1 treated U2OS cells exposed to the indicated doses of TRDMT1i (n = 3 biological replicates). Data are presented as mean values ± SEM. **(K–L)** Western blot analysis of lysates from siCtrl and siMSH2 treated CT26 cells (K) or U2OS (L), probed with anti-MSH2 and anti-GAPDH antibodies. GAPDH served as a loading control. **(M)** Colony formation assay of siCtrl and siMSH2 treated U2OS cells exposed to the indicated doses of TRDMT1i (n = 3 biological replicates). Data are presented as mean values ± SEM. Statistical significance was determined by two-tailed unpaired Student's t-tests to generate P values. All numeric values are available in the Source Data.

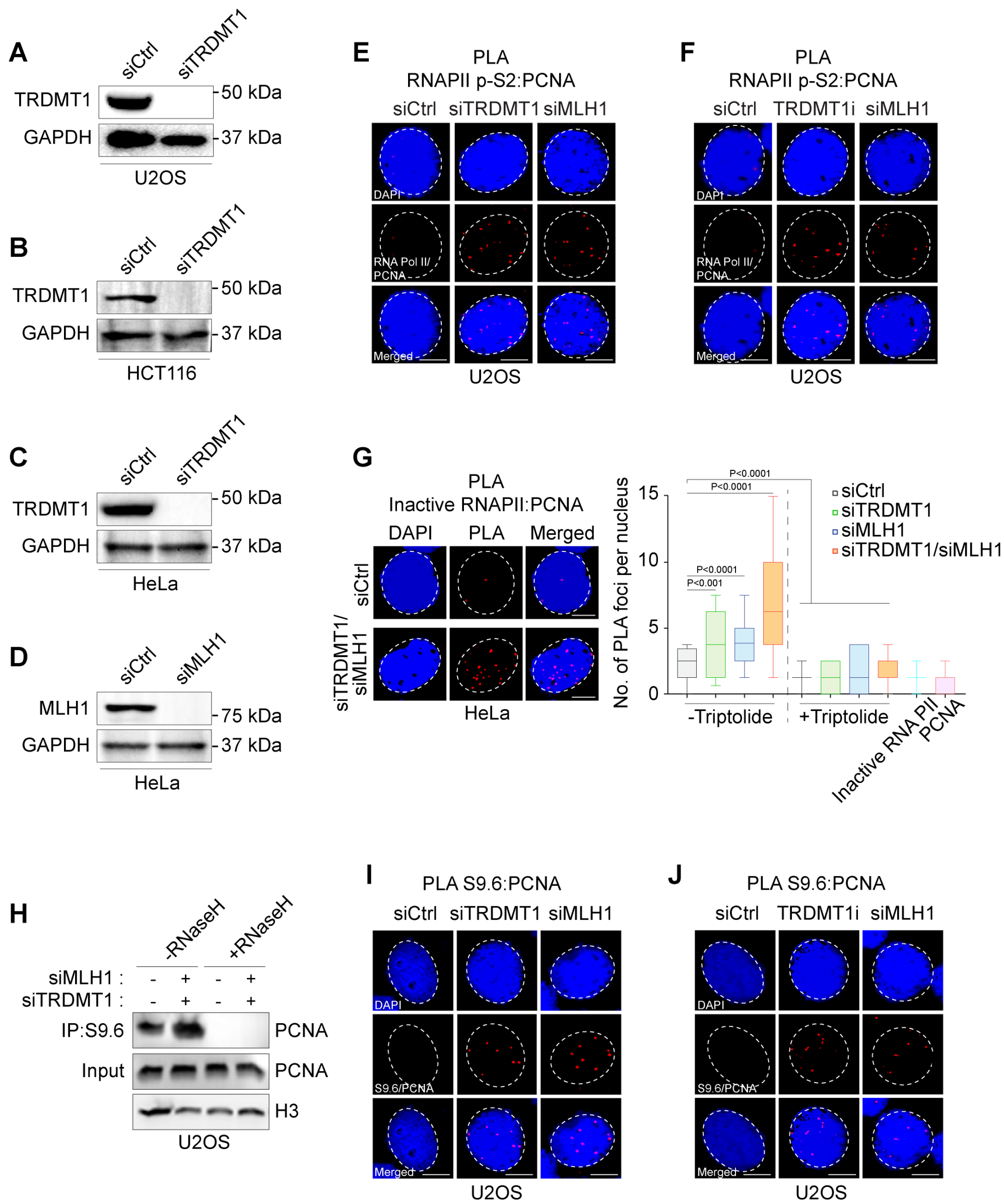

Supplementary Fig. 3. Effects of TRDMT1 and MLH1 deficiencies on TRCs. (A–C) Western blot analysis of lysates from U2OS (A), HCT116 (B), and HeLa (C) cells transfected with siCtrl or siTRDMT1, probed with anti-TRDMT1 and anti-GAPDH antibodies. GAPDH served as a loading control. **(D)** Western blot analysis of lysates from HeLa cells transfected with siCtrl or siMLH1, probed with anti-MLH1 and anti-GAPDH antibodies. GAPDH served as a loading control. **(E–F)** Representative images of PLA foci in U2OS cells transfected with control or indicated siRNAs, or treated with TRDMT1i, and analyzed by PLA using anti–RNAPII p-S2 and anti-PCNA antibodies, individually or in combination. **(G)** Representative images of PLA foci in U2OS cells transfected with control or indicated siRNAs and either untreated or treated with 1 µM triptolide for 4 h. PLA was performed using anti-PCNA and anti-RNAPII antibodies (n = 3 biological replicates, 150-160 cells per replicate). Scale bar, 5 µm. **(H)** Subcellular fractionation of U2OS cells transfected with control or indicated siRNAs and treated with or without RNaseH. Chromatin fragments with DNA-RNA hybrids were pulled down using anti-S9.6 antibody, followed by immunoblotting with anti-PCNA antibody. Histone H3 served as a chromatin loading control. **(I–J)** Representative images of PLA foci in U2OS cells transfected with control or indicated siRNAs, or treated with TRDMT1i, and analyzed by PLA using anti-S9.6 and anti-PCNA antibodies, individually or in combination. In all box plots, the center lines represent medians, the lower and upper bounds of the boxes represent the 25th and 75th percentiles, respectively, and the whiskers represent the minimum and maximum values. Statistical significance was determined by two-tailed unpaired Student's t-tests to generate P values. All numeric values are available in the Source Data.

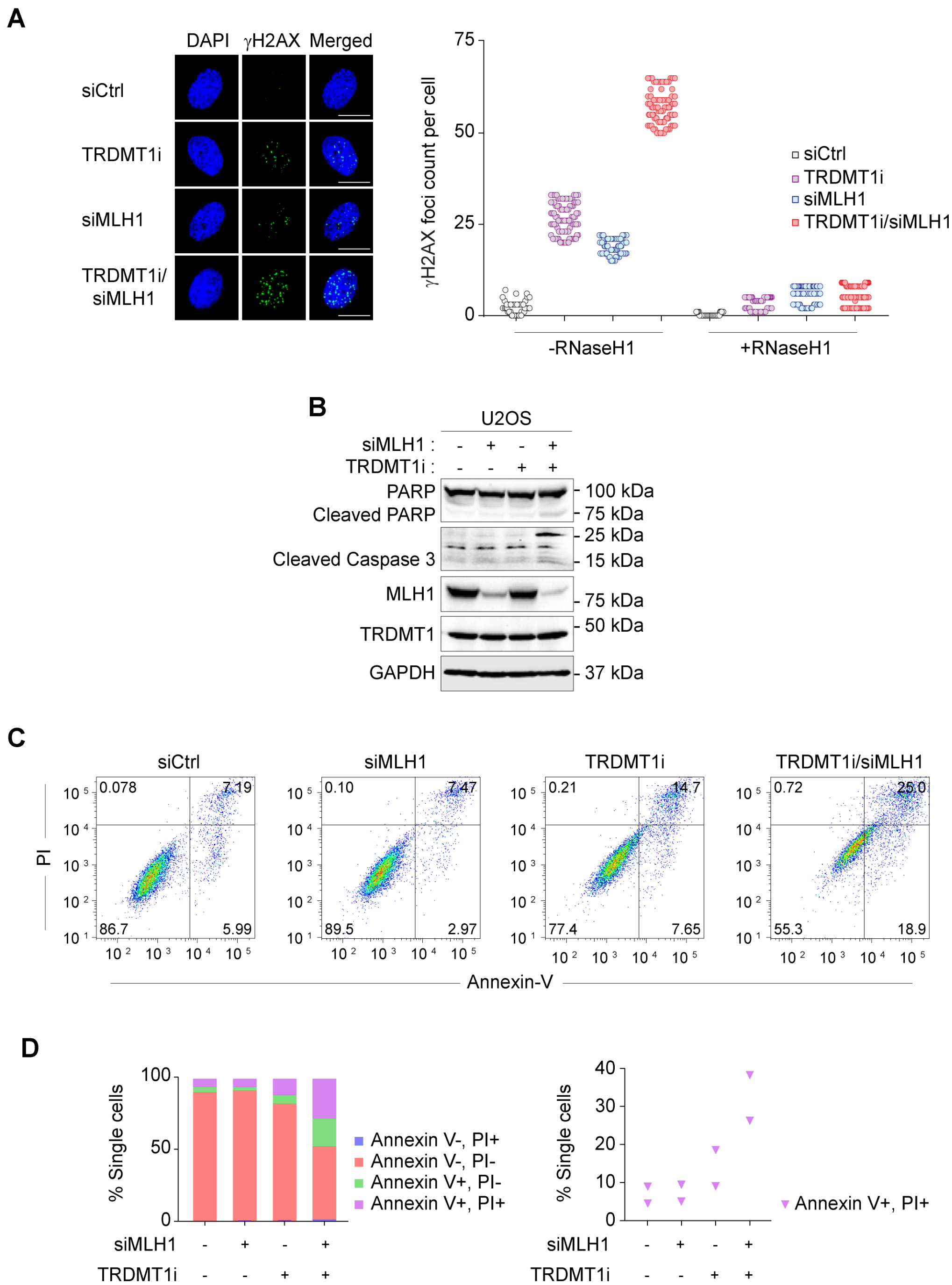

Supplementary Fig. 4. Combined loss of TRDMT1 and MLH1 induces R-loop-dependent DNA damage and apoptosis. (A) Representative immunofluorescence images and quantification of γH2AX foci in HeLa cells transfected with siCtrl or siMLH1, either untreated or treated with TRDMT1 inhibitor, with or without RNase H1 induction (n = 2 technical replicates, 50 cells per replicate). Scale bar, 5 µm. **(B)** Immunoblot analysis of apoptotic markers in HeLa cells transfected with control siRNA or siMLH1 and either untreated or treated with TRDMT1 inhibitor. PARP, cleaved PARP, cleaved caspase-3, MLH1, and TRDMT1 levels were analyzed by western blotting. GAPDH served as a loading control. **(C)** Representative Annexin V/PI flow-cytometry plots showing apoptosis in HeLa cells transfected with control siRNA or siMLH1, with or without TRDMT1 inhibition. **(D)** Quantification of Annexin V/PI populations from (C). Stacked bar plots show the distribution of viable, early apoptotic, late apoptotic, and PI-positive cells. Bar graphs show the percentage of Annexin V-positive/PI-positive cells. n=2 biological replicates. All numeric values are available in the Source Data.

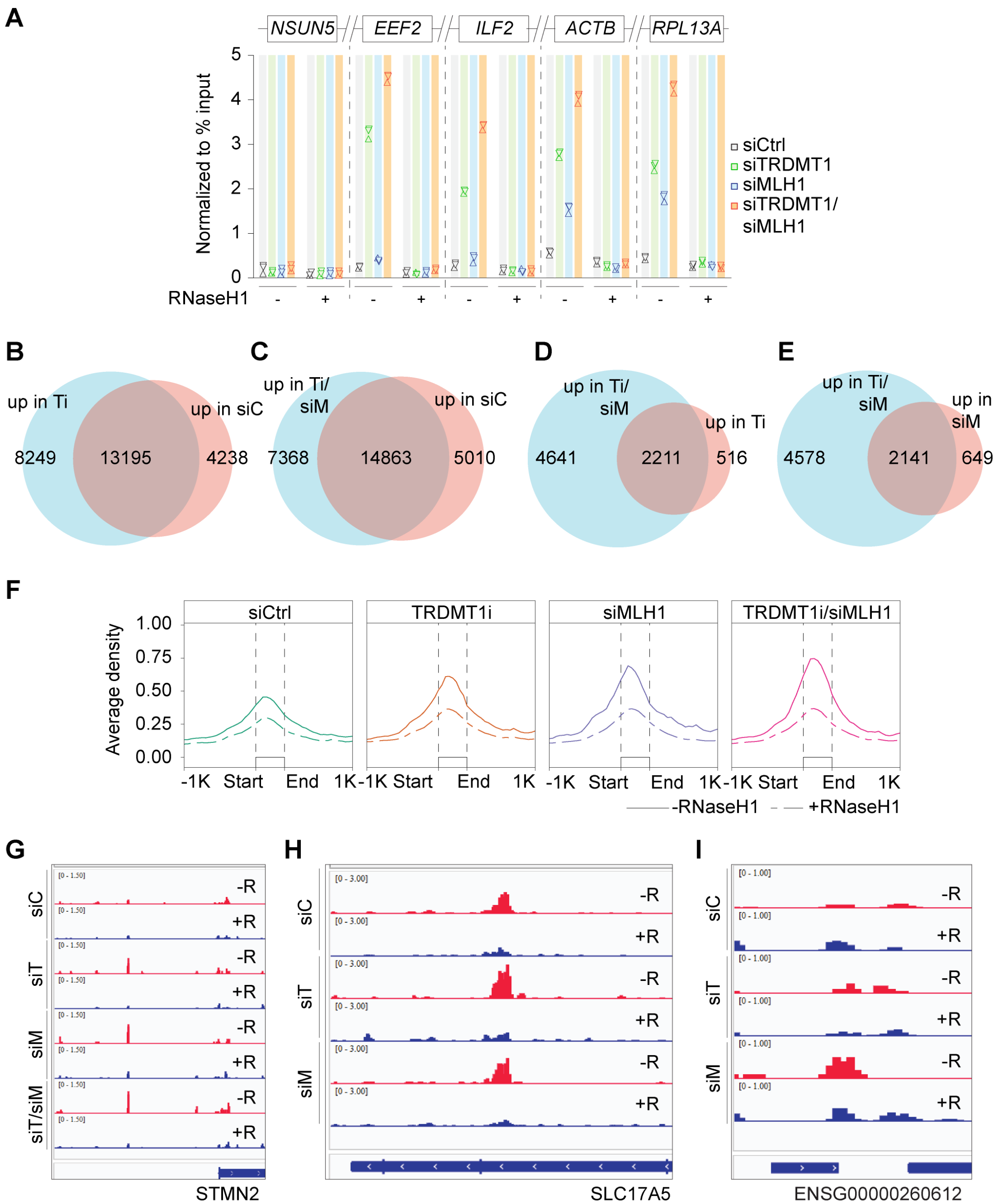

Supplementary Fig. 5. Effects of TRDMT1 and MLH1 deficiencies on R-loop accumulation. (A) Quantification of RNA/DNA hybrid accumulation by DRIP-qPCR in HeLa cells transfected with control or indicated siRNAs, with or without RNaseH1 induction, at the *NSUN5* (control), *EEF2* (m5C+), *ILF2* (m5C+), *ACTB*, and *RPL13A* loci. The same data in Fig. 3B was reanalyzed in this panel. Instead of fold increase relative to the siCtrl -RNaseH1 control, samples were analyzed for percent of input in this panel. n=2 biological replicates (3 independent sets of technical replicates). Datapoints from different biological replicates are shown as symbols in different shapes. **(B–E)** Venn diagrams showing shared and distinct R-loop peaks increased under different conditions: **(F)** Aggregated profiles of R-loop peaks upregulated in the TRDMT1i, siMLH1, and TRDMT1i/siMLH1 samples relative to siCtrl under ±RNaseH conditions. **(G–I)** IGV browser views showing representative examples of R-loop peaks in siTRDMT1, siMLH1, and siTRDMT1/siMLH1 samples compared with siCtrl under ±RNaseH1 conditions.

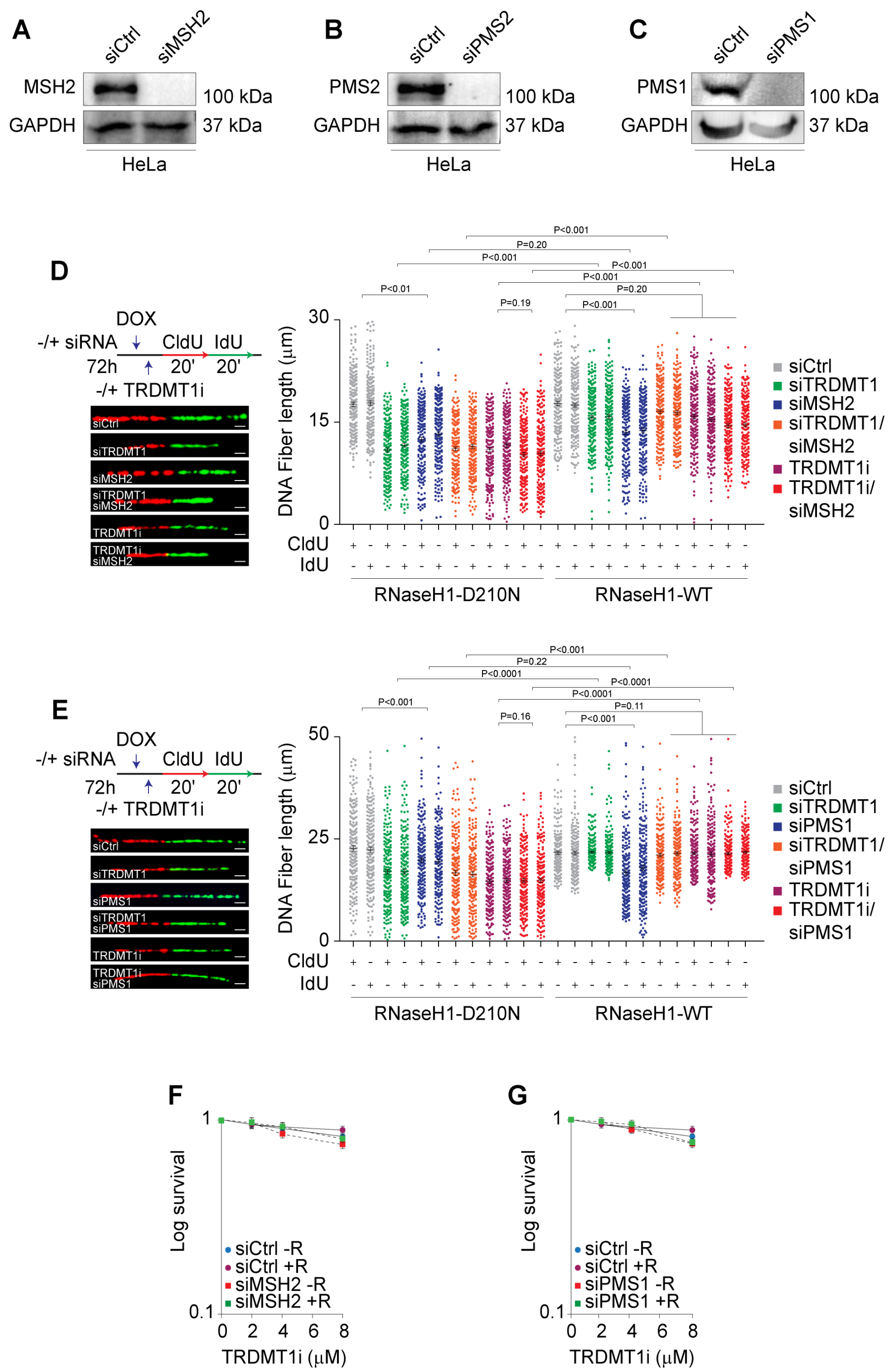

Supplementary Fig. 6. Effects of MSH2 and PMS1 deficiencies on replication progression and TRDMT1i sensitivity. (A–C) Western blot analysis of lysates from HeLa cells transfected with siCtrl or siMSH2 (A), siPMS2 (B), or siPMS1 (C), probed with anti-MSH2, anti-PMS2, or anti-PMS1 antibodies, respectively, together with anti-GAPDH antibody. GAPDH served as a loading control. **(D–E)** Schematic of the CldU/IdU pulse-labeling protocol, representative DNA fiber images, and quantification of DNA fiber lengths in HeLa cells inducible for RNaseH1-WT or RNaseH1-D210N expression and transfected with control or indicated siRNAs, or treated with TRDMT1i. Scatter plots show CldU and IdU tract lengths of individual forks (n = 3 biological replicates, 60-70 fibers per replicate). Data are presented as mean values ± SEM. Scale bar, 10 μm. **(F–G)** Colony formation assays in HeLa cells with or without RNaseH induction, transfected with indicated siRNAs and treated with increasing concentrations of TRDMT1i. n=3 biological replicates. Data are presented as mean values ± SEM. All statistical significance was determined by two-tailed unpaired Student's t-tests to generate P values. All numeric values are available in the Source Data.

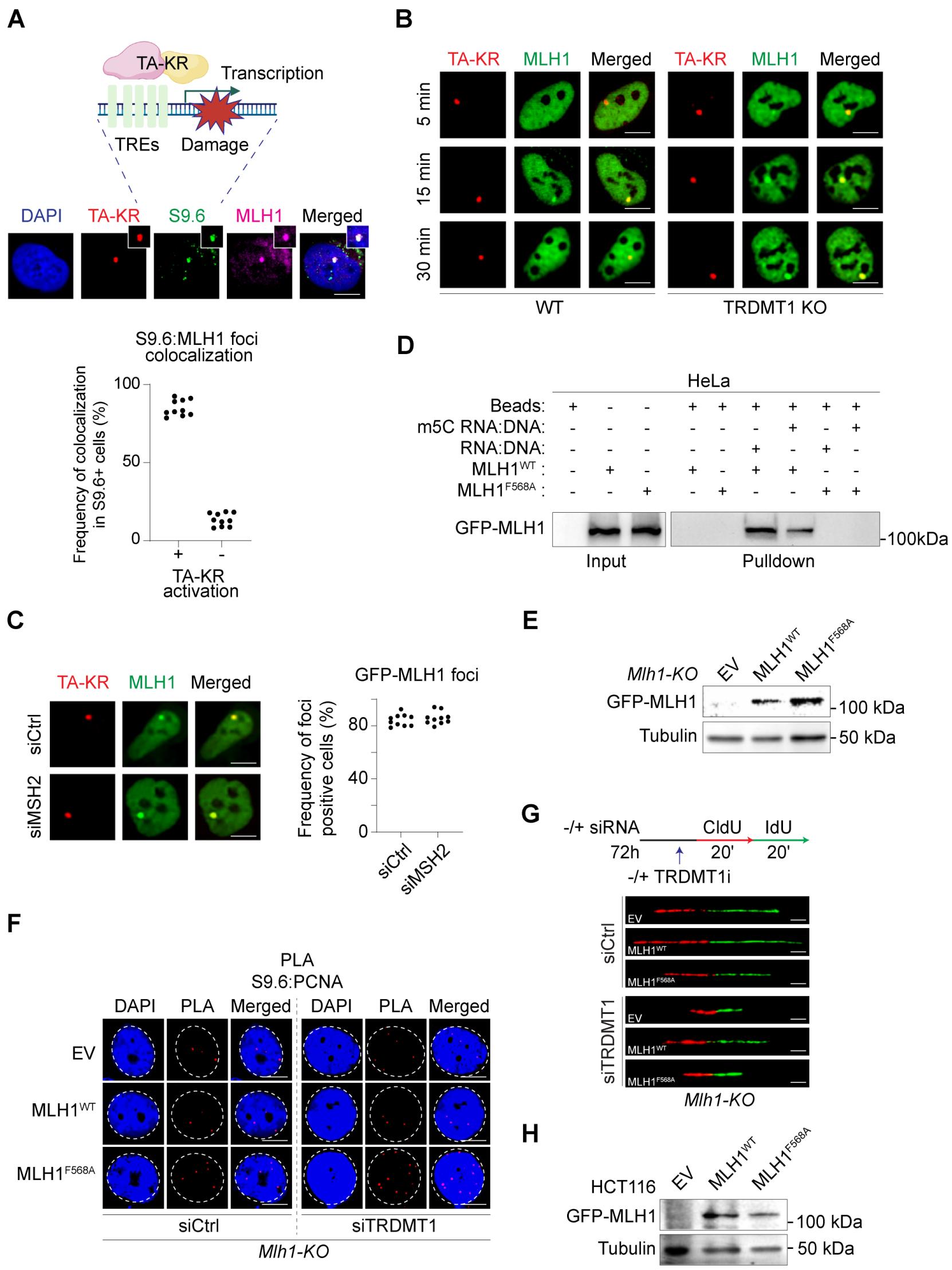

Supplementary Fig. 7. Characterization of the localization and function of MLH1 at R-loops. **(A)** U2OS-TRE cells were transfected with TA-KR and GFP-MLH1^{WT} plasmids, exposed to light irradiation for 20 min to activate KR, and allowed to recover for 10 min before imaging. Representative images and quantification of S9.6 and MLH1 foci colocalization upon TA-KR activation are shown. n = 1 biological replicate. Scale bar, 10 µm. **(B)** U2OS-TRE *TRDMT1-WT* or *TRDMT1-KO* cells were transfected with TA-KR and GFP-MLH1 plasmids, exposed to light irradiation for 10 min, and allowed to recover for the indicated times before microscopy. Scale bar, 10 µm. **(C)** U2OS-TRE cells transfected with indicated siRNAs together with TA-KR and GFP-MLH1 plasmids were exposed to light irradiation for 20 min and allowed to recover for 10 min before imaging. Scale bar, 10 µm. Quantification shows fold increase in GFP-MLH1 foci intensity at TA-KR sites. n = 1 biological replicate. **(D)** Cells expressing GFP-MLH1^{WT} or GFP-MLH1^{F568A} were subjected to pulldowns with unmethylated or methylated RNA–DNA hybrids. Bound fractions were analyzed for GFP-MLH1 by Western blot. **(E)** Western blot analysis of lysates from CT26 *Mlh1-KO* cells transfected with GFP-MLH1^{WT}, GFP-MLH1^{F568A}, or GFP-empty vector (EV), probed with anti-GFP and anti-tubulin antibodies. Tubulin served as a loading control. **(F)** Representative images showing S9.6:PCNA PLA foci in CT26 *Mlh1-KO* cells transfected with indicated siRNAs and expressing GFP-MLH1^{WT}, GFP-MLH1^{F568A}, or GFP-EV. **(G)** Schematic of the CldU/IdU pulse-labeling assay and representative images of DNA replication fork progression in CT26 *Mlh1-KO* cells transfected with indicated siRNAs and expressing GFP-MLH1^{WT}, GFP-MLH1^{F568A}, or GFP-EV. Scale bar, 10 µm. **(H)** Western blot analysis of lysates from HCT116 cells transfected with GFP-MLH1^{WT}, GFP-MLH1^{F568A}, or GFP-EV, probed with anti-GFP and anti-tubulin antibodies. All numeric values are available in the Source Data. Panel A was created with BioRender.com. Publication license: https://BioRender.com/3l2dv1v.

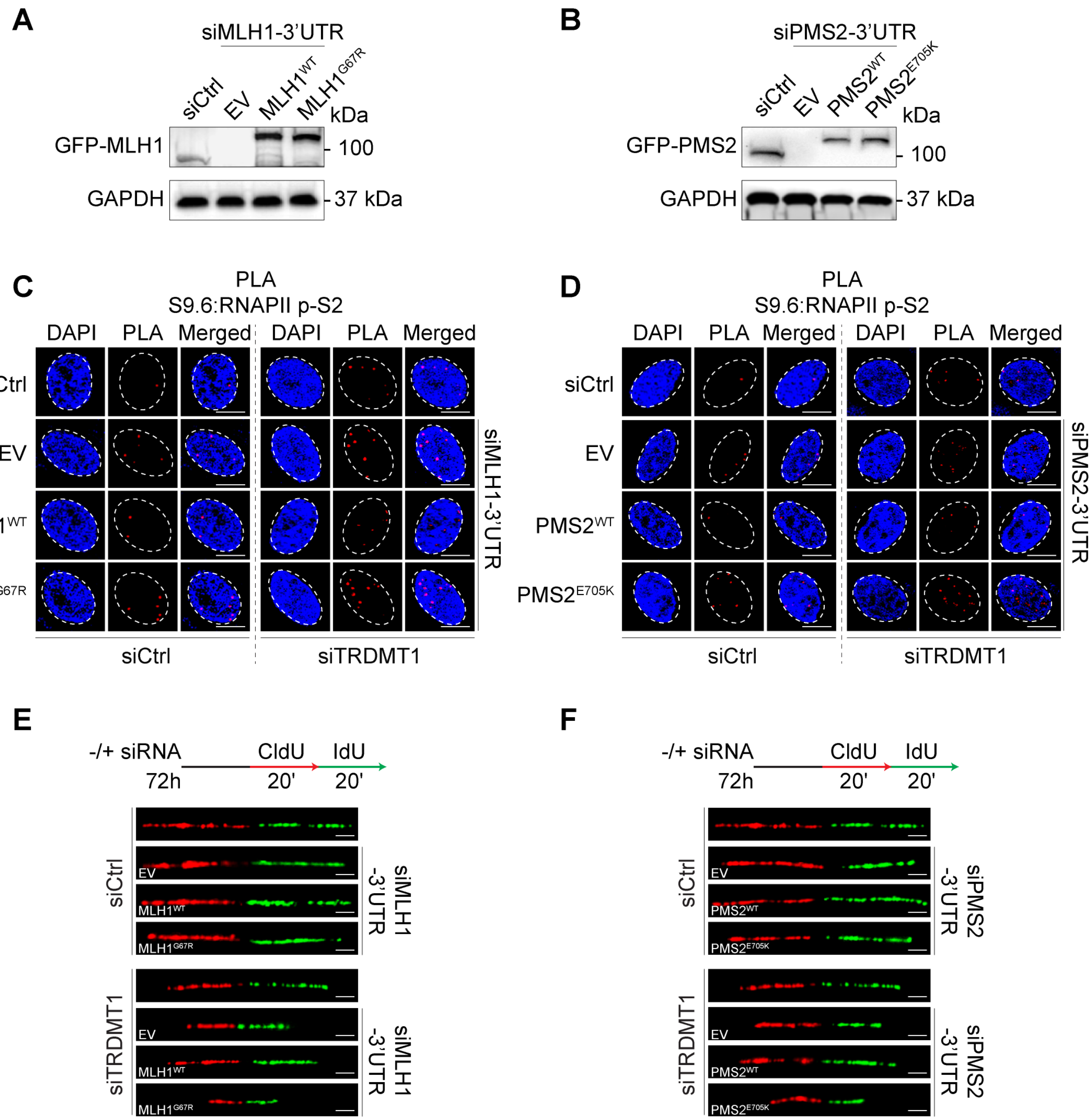

Supplementary Fig. 8. MutLα uses ATPase and nuclease activities to suppress TRCs. (A–B) Western blot analysis confirming expression of GFP-MLH1^{WT} or GFP-MLH1^{G67R} in HeLa cells depleted of endogenous MLH1 using siMLH1-3′UTR (A), and GFP-PMS2^{WT} or GFP-PMS2^{E705K} in cells depleted of endogenous PMS2 using siPMS2-3′UTR (B). Blots were probed with anti-GFP and anti-GAPDH antibodies. GAPDH served as a loading control. **(C–D)** Representative PLA images of HeLa cells transfected with siMLH1-3′UTR or siPMS2-3′UTR together with siCtrl or siTRDMT1. Cells were complemented with the corresponding WT or mutant MLH1 (C) or PMS2 (D) constructs, with or without RNase H1 induction, and analyzed by PLA using anti–RNAPII p-S2 and anti-PCNA antibodies, individually or in combination. **(E–F)** Schematic of the CldU/IdU pulse-labeling assay and representative DNA fiber length comparisons in HeLa cells transfected with siMLH1-3′UTR or siPMS2-3′UTR together with siCtrl or siTRDMT1. Cells were complemented with the corresponding WT or mutant MLH1 (E) or PMS2 (F) constructs. Scale bar, 10 μm.

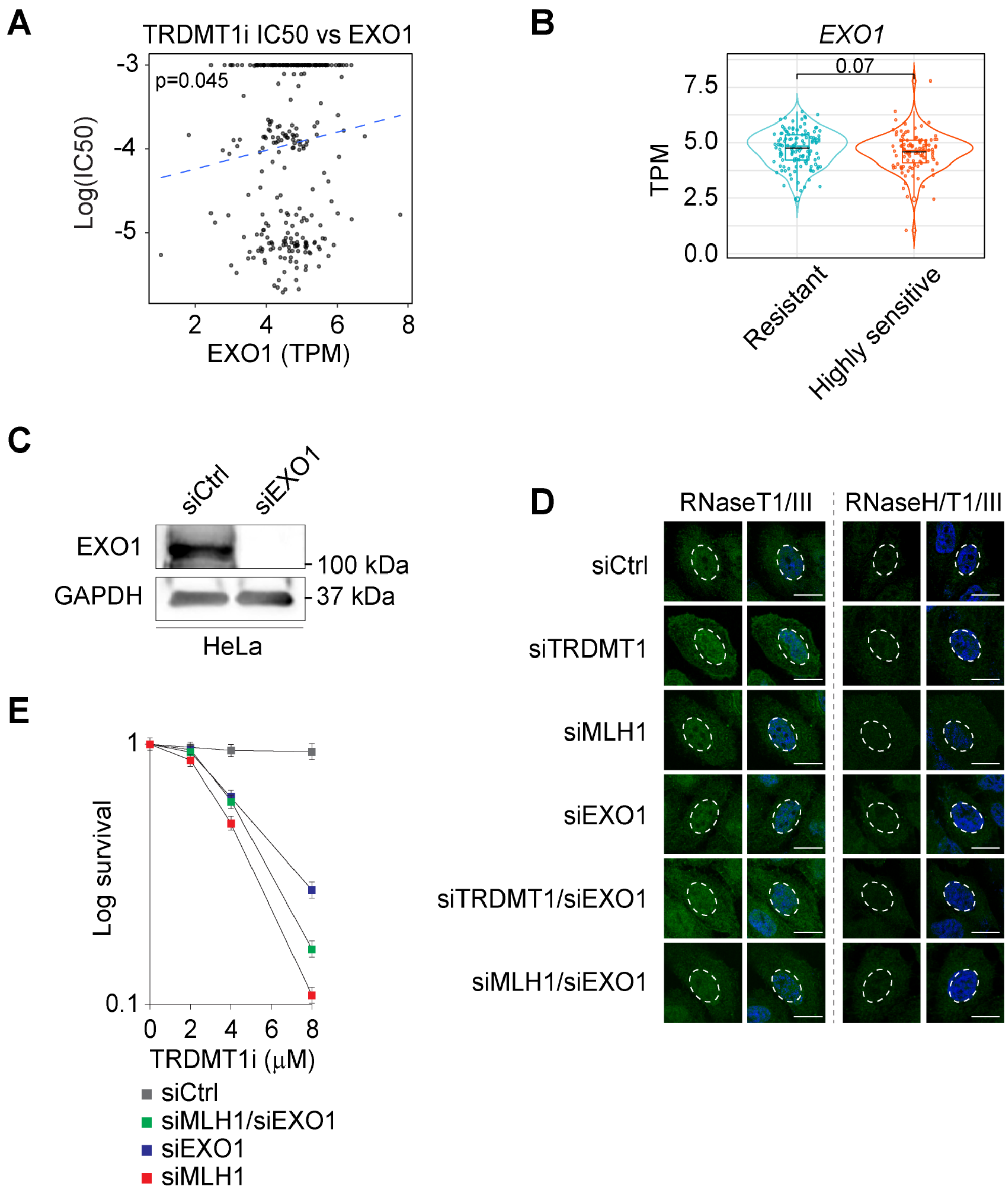

Supplementary Fig. 9. EXO1 suppresses R-loops and TRDMT1i sensitivity. (A) Correlation between EXO1 expression and TRDMT1 inhibitor IC50 values across cancer cell lines. Each dot represents one cell line. Statistical significance was determined by correlation analysis (P = 0.045). **(B)** Violin plot comparing EXO1 mRNA expression levels (TPM) in TRDMT1i-highly-sensitive and -resistant cancer cell lines. Each dot in violin plot represents one cell line. Violin widths represent the kernel density distribution of the data. Box plots show the median (centre line), the first and third quartiles (box limits), and whiskers extending to the most extreme observations within 1.5 × the interquartile range (IQR). The black horizontal line indicates the mean. Statistical significance was assessed using a two-sided Wilcoxon rank-sum test. Statistical significance was assessed using a two-sided Wilcoxon rank-sum test. **(C)** Western blot validation of EXO1 depletion in HeLa cells transfected with siEXO1. GAPDH served as a loading control. **(D)** Representative confocal images showing RNA–DNA hybrid accumulation detected by GFP-dRNH1 (green) in HeLa cells transfected with control or indicated siRNAs or treated with TRDMT1i. After fixation, cells were treated with mock, RNase T1/RNase III (T1/III), or RNaseH/RNase T1/RNase III (H/T1/III). **(E)** Colony formation assay in HeLa cells transfected with control or indicated siRNAs and treated with increasing concentrations of TRDMT1 inhibitor (n = 3 biological replicates). Data are presented as mean values ± SEM. All statistical significance was determined by two-tailed unpaired Student's t-tests to generate P values. All numeric values are available in the Source Data.

A

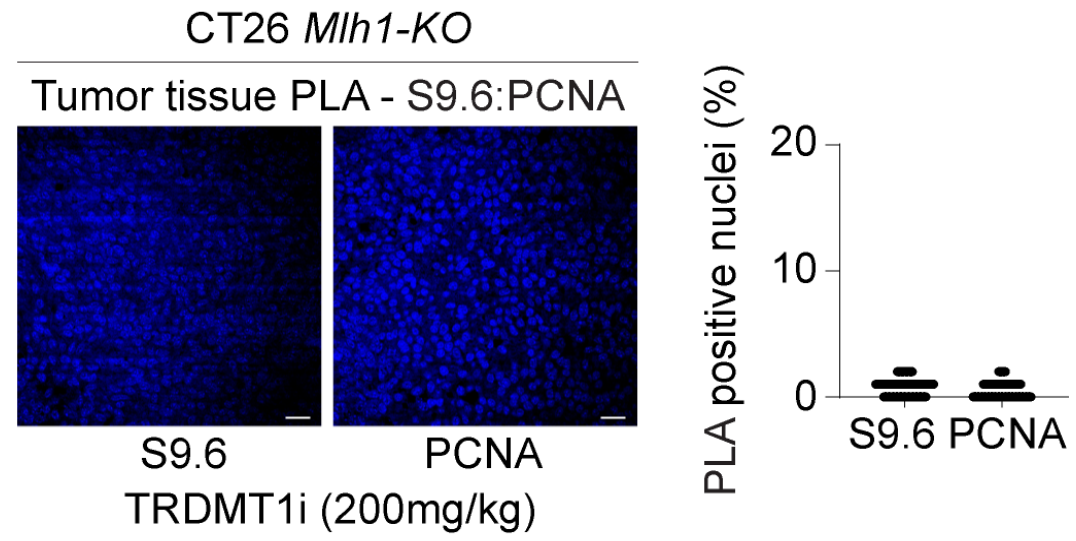

B

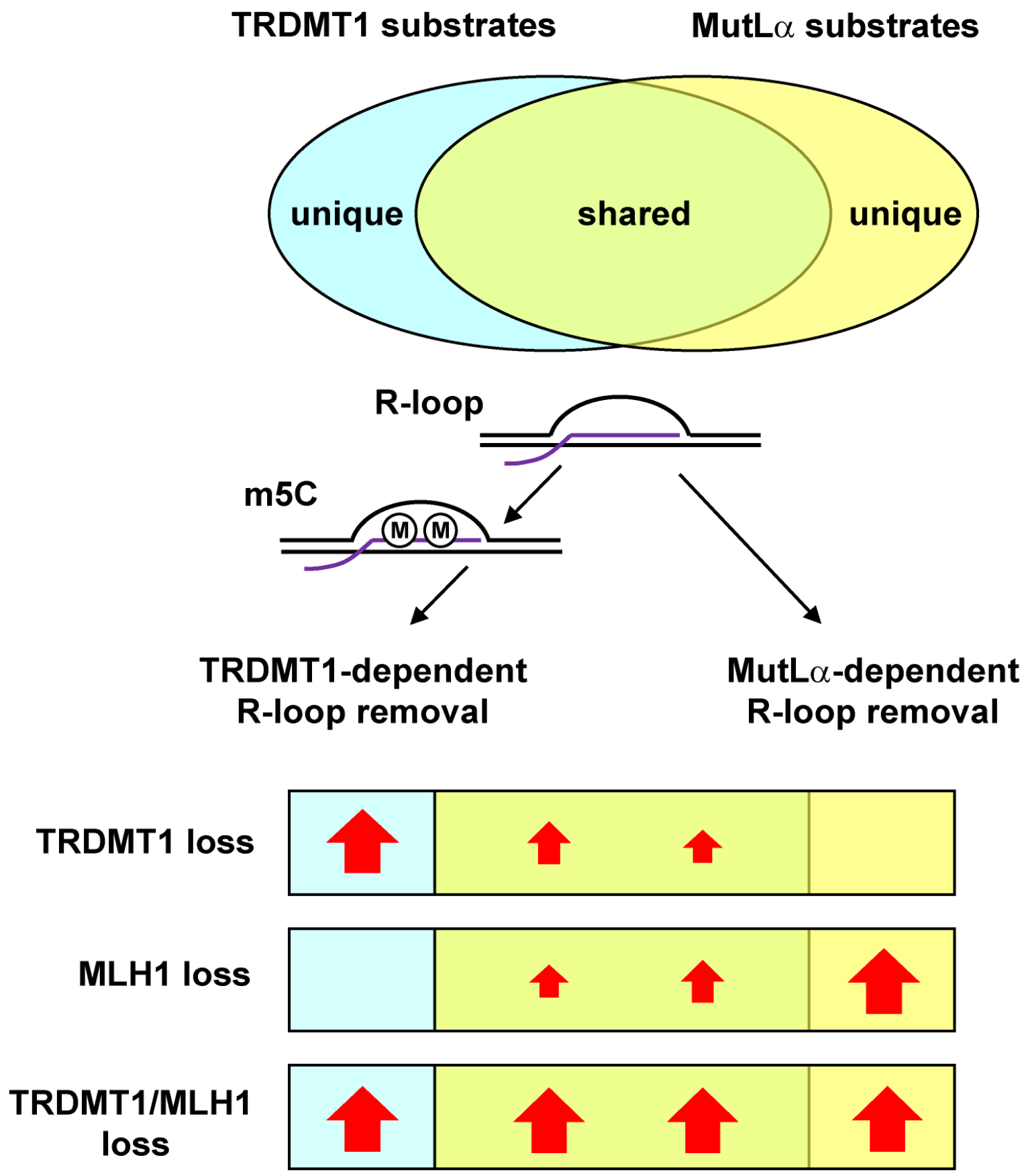

Supplementary Fig. 10. PLA analysis of TRDMT1i effects in vivo and a model for the coordinated roles of TRDMT1 and MutLα in R-loop suppression. (A) Representative images of nuclear PLA foci generated using anti-S9.6 and anti-PCNA antibodies in sections of CT26 *Mlh1-KO* tumor treated with TRDMT1i. PLA foci are shown in red and nuclei were stained with DAPI (blue). Quantification of nuclear PLA foci is shown in the right panel. n = 1 biological replicate. **(B)** Model illustrating the coordinated actions of TRDMT1 and MutLα in R-loop suppression. TRDMT1 and MutLα function at overlapping subsets of R-loops to prevent aberrant R-loop accumulation. TRDMT1 preferentially acts at R-loop substrates that efficiently form m5C. At shared R-loop substrates, m5C formation determines pathway choice between TRDMT1- and MutLα-mediated R-loop suppression. Loss of either TRDMT1 or MLH1 increases R-loops at their respective unique substrates and modestly elevates R-loops at shared substrates. Concurrent loss of TRDMT1 and MLH1 further increases R-loops at shared substrates and elevates R-loop levels across all substrates. All numeric values are available in the Source Data.

Supplementary Table 1. Antibodies and conditions used in this study

Antibody	Manufacturer	Working dilutions
TRDMT1	Santa Cruz	1:500
MLH1	Cell Signaling Technology	1:1000
PMS2	Cell Signaling Technology	1:1000
MSH2	Abcam	1:1000
PMS1	Abcam	1:1000
GAPDH	Cell Signaling Technology	1:2000
Tubulin	Cell Signaling Technology	1:2000
S9.6	Millipore	1:1000
S9.6	Kerafast	1:1000
PCNA	Santa Cruz	1:1000
RNA Pol II p-S2	Abcam	1:1000
γH2AX	Millipore	1:1000
GFP	Roche	1:1000
GFP	Novus	1:1000
KillerRed	Evrogen	1:1000
Ki-67	Cell Signaling Technology	1:500
Anti-BrdU rat (for DNA fiber)	Abcam	1:100
Anti-BrdU mouse (for DNA fiber)	BD Biosciences	1:100
Goat Anti-Rat Alexa Fluor 594 (for DNA fiber)	Invitrogen	1:100
Goat Anti-Mouse Alexa Fluor 488 (for DNA fiber)	Invitrogen	1:100
Anti-HRP rabbit (for WB)	Abcam	1:5000
Anti-HRP mouse (for WB)	Abcam	1:5000
Rabbit anti-Mouse IgG (H+L) Secondary Antibody	Thermo Fisher Scientific	1:100
Anti-Rabbit IgG, Native antibody, Mouse monoclonal	Sigma	1:100
EXO1	Proteintech	1:1000
FMRP	Abcam	1:1000
PCNA	Abcam	1:1000
H3	Cell Signaling Technology	1:1000
PARP	Cell Signaling Technology	1:1000
Cleaved Caspase-3	Cell Signaling Technology	1:500
