# Supplementary material for: Suppression of transcription-replication conflicts by sequence-coordinated actions of TRDMT1 and MutLα
Source: Nat Commun. 2026 Aug 10;17:9566. doi: 10.1038/s41467-026-76137-8 (PMC13550614; doi:10.1038/s41467-026-76137-8)
Supplement: Supplementary file 2 [file 41467_2026_76137_MOESM2_ESM.pdf]

nature portfolio

Corresponding author(s): NCOMMS-25-102189-T

Last updated by author(s): Jul 20, 2026

Reporting Summary

Nature Portfolio wishes to improve the reproducibility of the work that we publish. This form provides structure for consistency and transparency in reporting. For further information on Nature Portfolio policies, see our Editorial Policies and the Editorial Policy Checklist.

Statistics

For all statistical analyses, confirm that the following items are present in the figure legend, table legend, main text, or Methods section.

n/a	Confirmed	
☐	☒	The exact sample size (*n*) for each experimental group/condition, given as a discrete number and unit of measurement
☐	☒	A statement on whether measurements were taken from distinct samples or whether the same sample was measured repeatedly
☐	☒	The statistical test(s) used AND whether they are one- or two-sided<br>*Only common tests should be described solely by name; describe more complex techniques in the Methods section.*
☒	☐	A description of all covariates tested
☒	☐	A description of any assumptions or corrections, such as tests of normality and adjustment for multiple comparisons
☐	☒	A full description of the statistical parameters including central tendency (e.g. means) or other basic estimates (e.g. regression coefficient) AND variation (e.g. standard deviation) or associated estimates of uncertainty (e.g. confidence intervals)
☐	☒	For null hypothesis testing, the test statistic (e.g. *F*, *t*, *r*) with confidence intervals, effect sizes, degrees of freedom and *P* value noted<br>*Give P values as exact values whenever suitable.*
☒	☐	For Bayesian analysis, information on the choice of priors and Markov chain Monte Carlo settings
☒	☐	For hierarchical and complex designs, identification of the appropriate level for tests and full reporting of outcomes
☒	☐	Estimates of effect sizes (e.g. Cohen's *d*, Pearson's *r*), indicating how they were calculated

Our web collection on statistics for biologists contains articles on many of the points above.

Software and code

Policy information about availability of computer code

Data collection	EnVision plate reader (PerkinElmer), Leica Stellaris confocal microscope, Zeiss Axio Imager Z2 upright microscope, Bio-Rad ChemiDoc MP imaging system, Bio-Rad CFX Opus 384 Real-Time PCR System, Agilent Tape station 4150, The BD LSRFortessa X-20 analyzer
Data analysis	GraphPad Prism 10, Bio-Rad Image Lab, FIJI, Zeiss ZEN 3.4 (blue edition), Sysmex Case Viewer

For manuscripts utilizing custom algorithms or software that are central to the research but not yet described in published literature, software must be made available to editors and reviewers. We strongly encourage code deposition in a community repository (e.g. GitHub). See the Nature Portfolio guidelines for submitting code & software for further information.

Data

Policy information about availability of data

All manuscripts must include a data availability statement. This statement should provide the following information, where applicable:

- Accession codes, unique identifiers, or web links for publicly available datasets
- A description of any restrictions on data availability
- For clinical datasets or third party data, please ensure that the statement adheres to our policy

The CUT&TAG sequencing raw datasets has been deposited Sequence Read Archive (SRA) and are available with the accession number PRJNA1210604 (https://www.ncbi.nlm.nih.gov/sra/PRJNA1210604).

Research involving human participants, their data, or biological material

Policy information about studies with human participants or human data. See also policy information about sex, gender (identity/presentation), and sexual orientation and race, ethnicity and racism.

Reporting on sex and gender	N/A
Reporting on race, ethnicity, or other socially relevant groupings	N/A
Population characteristics	N/A
Recruitment	N/A
Ethics oversight	N/A

Note that full information on the approval of the study protocol must also be provided in the manuscript.

Field-specific reporting

Please select the one below that is the best fit for your research. If you are not sure, read the appropriate sections before making your selection.

☒ Life sciences ☐ Behavioural & social sciences ☐ Ecological, evolutionary & environmental sciences

For a reference copy of the document with all sections, see nature.com/documents/nr-reporting-summary-flat.pdf

Life sciences study design

All studies must disclose on these points even when the disclosure is negative.

Sample size	Sample sizes, numbers of replicates, error bars, and statistical tests were selected according to accepted practices and are specified in the figure legends. Unless otherwise stated, experiments were independently performed with three biological replicates. Experiments conducted with fewer than three biological replicates are explicitly mentioned in the figure legends.<br>For animal experiments, sample sizes were set at the minimum necessary to reduce cost while maintaining adequate statistical power to detect differences between experimental groups (n = 5).
Data exclusions	No data were excluded in the analysis.
Replication	Unless otherwise stated, experiments were independently performed with three biological replicates. Experiments conducted with fewer than three biological replicates are explicitly mentioned in the figure legends.
Randomization	For cell-based experiments, cells were randomly selected from the population. No randomization was performed for animal experiments.
Blinding	Investigators were not blinded to experimental conditions during data collection and analysis.

Reporting for specific materials, systems and methods

We require information from authors about some types of materials, experimental systems and methods used in many studies. Here, indicate whether each material, system or method listed is relevant to your study. If you are not sure if a list item applies to your research, read the appropriate section before selecting a response.

Materials & experimental systems

n/a	Involved in the study	
☐	☒	Antibodies
☐	☒	Eukaryotic cell lines
☒	☐	Palaeontology and archaeology
☐	☒	Animals and other organisms
☒	☐	Clinical data
☒	☐	Dual use research of concern
☒	☐	Plants

Methods

n/a	Involved in the study	
☒	☐	ChIP-seq
☐	☒	Flow cytometry
☒	☐	MRI-based neuroimaging

Antibodies

Antibodies used	TRDMT1 Santa Cruz sc-365001; RRID: AB_10708139<br>MLH1 Cell Signaling Technology 4256S; RRID: AB_10545447<br>PMS2 Cell Signaling Technology 27884; RRID: AB_3094662<br>MSH2 Abcam ab52266; RRID: AB_2144800<br>PMS1 Abcam Ab315798; RRID: N/A<br>gH2AX Millipore 05-636-I; RRID: AB_2755003<br>GFP Roche 11814460001; RRID: AB_390913<br>Ki-67 Cell Signaling Technology 9129; RRID: AB_2687446<br>Anti-BrdU rat (for DNA fiber) Abcam ab6326; RRID: AB_305426<br>Anti-BrdU mouse (for DNA fiber) BD Biosciences 347580; RRID: AB_400326<br>Goat Anti-Rat Alexa Fluor 594 (for DNA fiber) Invitrogen A-11007; RRID: AB_10561522<br>Goat Anti-Mouse Alexa Fluor 488 (for DNA fiber) Invitrogen A-11001; RRID: AB_2534069<br>Anti-HRP rabbit (for WB) Abcam ab6721; RRID: AB_955447<br>Anti-HRP mouse (for WB) Abcam ab6728; RRID: AB_955440<br>Rabbit anti-Mouse IgG (H+L) Secondary Antibody Thermo Fisher Scientific 31188; RRID: AB_228419<br>Anti-Rabbit IgG, Native antibody, Mouse monoclonal Sigma R2655; RRID: AB_1079113<br>GAPDH Cell Signaling Technology 5174S; RRID: AB_10622025<br>Tubulin Cell Signaling Technology 2144S; RRID: AB_2210548<br>S9.6 Millipore MABE1095; RRID: AB_2861387<br>PCNA Abcam ab18197; RRID: AB_444313<br>RNA Pol II p-S2 Abcam ab5095; RRID: AB_304749<br>EXO1 Proteintech 16253-1-AP; RRID:AB_2278140<br>FMRP Abcam ab17722; RRID:AB_2278530<br>H3 Cell Signaling Technology 14269S; RRID:AB_2756816<br>PARP Cell Signaling Technology 9542; RRID:AB_2160739<br>Cleaved Caspase-3 Cell Signaling Technology 9661; RRID:AB_2341188
Validation	All antibodies used in this study were commercially available and have been validated by the suppliers as well as in previous publications. Detailed validation data are available on the respective manufacturers' websites. Antibodies were used in accordance with the manufacturers' instructions.

Eukaryotic cell lines

Policy information about cell lines and Sex and Gender in Research

Cell line source(s)	U2OS ATCC HTB-96<br>U2OS TRDMT1 KO Generated for this study N/A<br>HeLa ATCC CRM-CCL-2<br>HeLa RNaseH WT Generated for this study N/A<br>HeLa RNaseH D210N Generated for this study N/A<br>HCT116 ATCC CCL-247<br>CT26 ATCC CRL-2638<br>CT26 MLH1 KO Clone #13 Generated for this study N/A
Authentication	Cell lines were not authenticated.
Mycoplasma contamination	All cell lines were routinely tested and found to be free of mycoplasma contamination.
Commonly misidentified lines<br>(See ICLAC register)	No commonly misidentified lines were used.

Animals and other research organisms

Policy information about studies involving animals; ARRIVE guidelines recommended for reporting animal research, and Sex and Gender in Research

Laboratory animals	Mouse BALB/c; NCI BALB/cAnNCr; Charles River Laboratories; #555
Wild animals	N/A
Reporting on sex	All mice used were female
Field-collected samples	N/A
Ethics oversight	All animals were housed and the experiments were conducted in accordance with the guidelines established by the Institutional Animal Care and Use Committee at the Duke University.

Note that full information on the approval of the study protocol must also be provided in the manuscript.

Plants

Seed stocks	N/A
Novel plant genotypes	N/A
Authentication	N/A

Flow Cytometry

Plots

Confirm that:

- [ ] The axis labels state the marker and fluorochrome used (e.g. CD4-FITC).
- [x] The axis scales are clearly visible. Include numbers along axes only for bottom left plot of group (a 'group' is an analysis of identical markers).
- [x] All plots are contour plots with outliers or pseudocolor plots.
- [x] A numerical value for number of cells or percentage (with statistics) is provided.

Methodology

Sample preparation	The details of sample preparation has been mentioned in the method section of the manuscript.
Instrument	The BD LSRFortessa X-20 analyzer was used for data acquisition.
Software	The FACS diva software was used for Data acquisition and Flow Jo software (version 10.9) was used for data analysis.
Cell population abundance	A total of 10,000 events were recorded for each sample.
Gating strategy	The cell populations were visualized using Forward scatter (FSC) and side scatter (SSC). Since, the Annexin V/PI assay looks at both live and dead cells, all cells were visualized for Annexin V/PI staining after identification and exclusion of doublets using FSC-A vs FSC-H. Annexin V signal was detected and measured using FITC emission channel and PI was measured using the PE-Cy5 emission channel.

- [ ] Tick this box to confirm that a figure exemplifying the gating strategy is provided in the Supplementary Information.
