# Supplementary material for: Suppression of transcription-replication conflicts by sequence-coordinated actions of TRDMT1 and MutLα
Source: Nat Commun. 2026 Aug 10;17:9566. doi: 10.1038/s41467-026-76137-8 (PMC13550614; doi:10.1038/s41467-026-76137-8)
Supplement: Supplementary file 3 [file 41467_2026_76137_MOESM3_ESM.pdf]

nature portfolio

Peer Review File

Suppression of Transcription-Replication Conflicts by Sequence-Coordinated Actions of TRDMT1 and MutLα

Corresponding Author: Dr Lee Zou

This file contains all reviewer reports in order by version, followed by all author rebuttals in order by version.

Version 0:

Reviewer comments:

Reviewer #1

(Remarks to the Author)
This manuscript identifies a novel and potentially therapeutically relevant synthetic lethal interaction between MutLα (MLH1-PMS2) deficiency and inhibition of the RNA methyltransferase TRDMT1. The work compellingly demonstrates that both factors function in parallel to suppress co-transcriptional R-loops, transcription-replication conflicts (TRCs), and associated DNA damage. The in vivo efficacy of a TRDMT1 inhibitor against MLH1-deficient tumors is particularly promising. However, as outlined below, several major conceptual and mechanistic gaps significantly weaken the support for the central claims. Addressing these concerns is essential to establish the proposed model and validate the therapeutic hypothesis.
Major Concerns:
1. The Mechanistic Role of MutLα in R-loop Suppression is Undefined. The claim that MutLα suppresses R-loops independently of its canonical MMR role is not substantiated. The manuscript fails to investigate whether known MutLα activities (ATP hydrolysis, endonuclease function) are required for this proposed novel function. Direct experiments are needed to elucidate the mechanism. This may include expressing separation-of-function MutLα mutants (e.g., ATPase-dead, endonuclease-dead) in MLH1-KO cells to test their ability to rescue R-loop accumulation, fork progression defects, and TRDMT1i sensitivity.
2. The Link Between Genomic Instability, MSI, and TRDMT1i Sensitivity is Ambiguous. The assertion that cancers with "high genomic instability" are sensitive is not quantitatively supported. Since MutLα-deficiency causes MSI, it is critical to distinguish whether TRDMT1i sensitivity is specifically tied to the MSI phenotype, the underlying R-loop burden, or a broader genomic instability signature. Is it possible that the authors could calculate a quantitative genomic instability index (e.g., integrating CIN score, loss of heterozygosity, MSI status) for the cell line panel and correlate it directly with TRDMT1i IC50 values?
3. Lack of Evidence that R-loop Accumulation is the Primary Lethal Driver. This is a critical omission. If R-loop accumulation is the primary driver of synthetic lethality, the authors should demonstrate that RNase H1 overexpression rescues not only cell viability but also DNA damage (e.g., γH2AX) in TRDMT1i-treated, MutLα-deficient cells. Without this key experiment, the causal chain remains speculative.
4. The Mechanism of Cell Death is Not Investigated. The cellular consequence of TRDMT1 inhibition in MutLα-deficient cells (apoptosis, necrosis, mitotic catastrophe, senescence) is entirely undefined. Characterizing the mode of cell death using standard assays is essential for understanding the therapeutic outcome.
5. The Contribution of Endogenous TRDMT1 Expression Levels is Unclear. Since high TRDMT1 expression correlates with inhibitor sensitivity, it is vital to rule out that the observed synthetic lethality is not confounded by higher basal TRDMT1 expression/activity in the MLH1-deficient models used. The authors should quantify and report TRDMT1 mRNA and protein levels in the isogenic cell pairs (MLH1 WT vs. KO) and perform a correlation analysis of MLH1 and TRDMT1 expression across the broader cell line panel.
Minor Concerns:
1. Discrepancy in PLA Foci Detection: The absence of baseline S9.6:PCNA PLA foci in in vivo MLH1-KO tumors (Fig. 6F) compared to in vitro data (Ext. Data Fig. 3G) needs explanation. Comment on assay sensitivity or potential in vivo compensation.
2. Nomenclature: Correct "gH2AX" to "γH2AX" throughout the manuscript and figures.
3. Figure Citations: Verify and correct erroneous figure panel citations (e.g., Page 11 reference to Ext. Data Fig. 5c).
4. Typographical Error: Confirm the cell concentration for the DNA fiber assay ("200 cells/mL" is likely incorrect).
5. Gene Nomenclature: Use correct mouse gene symbols (e.g., Mlh1-KO CT26, not MLH1-KO CT26).
6. Data Interpretation: Re-evaluate and clarify the description of the data in Fig. 3B, particularly regarding the ILF2 locus, to

ensure accuracy.
7. Figure Quality: Provide a cleaner western blot for Fig. 5D to unequivocally demonstrate the specific MLH1-PMS2 interaction.
8. In Vivo Dosing: Justify the high doses of TRDMT1i used in vivo (100-200 mg/kg) with references or discuss potential toxicity.

Reviewer #2

(Remarks to the Author)
This is an excellent study on new roles of MutLa mismatch repair complex in coordination with the m5C RNA methyla trasferase TRDMT1. The study uncover a cooperative functions of these two factors to suppress co-transcriptional R-loops accumulation and prevent harmful transcription–replication conflicts. Loss of either pathway slows DNA replication, but combined loss causes more severe replication fork stalling, R-loop accumulation, and synthetic lethality, Importantly, cancer cells lacking MLH1 or PMS2 are highly dependent on TRDMT1, and pharmacological inhibition of TRDMT1 selectively impairs growth of MLH1-deficient tumors in vivo. This work is relevant because it expands on the idea that R-loop are regulated by RNA methylation–and proposes TRDMT1 inhibition as a targeted therapeutic strategy for MutLα-deficient cancers. I globally support pubblication of this study in Nature Comm. I only have few suggestions

1) My main concern is on the extensive use of PLA assay for RNApII and PCNA as a read out of TRC. Despite PLA is a powerfull approach, it can be prone to folse positive results due to interaction amonge the relevant factors elsewhere in the nuceloplasms on on chromatin. PLA results has been coupled with ChIP -qPCR for either PCNA and RNA ppolII in the different conditions, since CUT&TAG approach in the study identify the relevat genomic context in which TRC likely occurs. I believe it would be reassuring to complement main PLA results with more standard appraches like Co-IP RNA immunoprecipitaion or DRIP.
2) Suppression of TRC with a treatment of any RNApolII inhibitor (DRB for example) could be informative in the different condition also to undestand if R-loops form also involving pre existing transcris.

Reviewer #3

(Remarks to the Author)
This study identifies a synthetic-lethal interaction between the RNA methyltransferase TRDMT1 and the mismatch repair complex MutLα (MLH1–PMS2), independent of canonical mismatch repair. Through a large-scale cancer cell line screen, the authors show that MLH1 or PMS2 loss confers strong sensitivity to a first-in-class TRDMT1 inhibitor, driven by excessive R-loop accumulation, transcription–replication conflicts (TRCs), and replication fork slowing. Mechanistically, TRDMT1 and MutLα independently recognize RNA–DNA hybrids and suppress overlapping but distinct subsets of R-loops, with RNA m5C modification dictating pathway choice. Importantly, TRDMT1 inhibition selectively suppresses MLH1-deficient tumor growth in vivo by inducing R-loop–mediated replication stress.
Overall, the manuscript is mechanistically sound, and clinically relevant, well suited for Nature Communications. The core conclusions are generally well supported, and the work advances understanding of how RNA modifications and DNA repair factors cooperate to suppress transcription–replication conflicts. Addressing the biochemical mechanism of non-canonical role of MutLα at R-loops would further elevate the impact, but even in its current form, the study findings are original and represents a major conceptual advance in the field.
The use of multiple orthogonal approaches to detect R-loops (PLA, GFP-dRNaseH1 staining, DRIP-qPCR, CUT&Tag), combined with functional rescue by RNaseH1, provides compelling evidence that R-loop accumulation is causal for TRCs, fork slowing, and synthetic lethality. Experimental approaches are appropriate, well controlled, and described in sufficient detail to allow reproduction. The integration of cell-based assays, genome-wide profiling, and in vivo tumor models strengthens the overall rigor of the study.
Points for Improvement and Clarification:
There are some areas that could be improved to further enhance mechanistic clarity and impact.
1. Direct biochemical activity of MutLα at R-loops. The data convincingly show recruitment and functional relevance of MutLα at R-loops, but direct biochemical evidence demonstrating whether MutLα cleaves RNA–DNA hybrids or displaced ssDNA strands is lacking. If testing this experimentally is technically not feasible, a discussion about its possible mechanistic role should be included.
2. Downstream R-loop resolution machinery. The manuscript does not identify which helicases or nucleases act downstream of MutLα-mediated R-loop recognition or incision. Genetic or epistasis analyses with known R-loop regulators (e.g., SETX) would add depth.

3. Mechanism of TRDMT1-mediated R-loop suppression in undamaged cells. While prior work implicates FMRP/TET1 at DNA damage sites, the identity of m5C “readers” or effectors operating at co-transcriptional R-loops in unperturbed cells remains speculative. Even limited exploratory data would strengthen this aspect.

Addressing the mechanistic points above experimentally where possible but in text otherwise would further strengthen the manuscript.

Minor Points:
4. There are several instances of inconsistent terminology (e.g., MutLa, MutLα, MHL1 instead of MLH1 in a few figure labels/text passages) that should be standardized throughout the manuscript.

5. In figure 5, the zoomed insets of IF images do not seem to show any added details. Either show more enlarged images or delete the zoomed boxes.

6. Minor typographical errors are present, such as occasional misspellings (e.g., “TRMT1” instead of “TRDMT1,” “PNCA” instead of “PCNA”) and missing or extra spaces in gene/protein names.

7. Some sentences in the Introduction and Discussion are overly long and dense and would benefit from light editing for clarity and readability.

8. Figure legends occasionally repeat information already provided in the main text and could be streamlined to improve clarity.

9. Ensure consistent gene/protein nomenclature (italicized gene names vs. non-italicized proteins) and uniform use of abbreviations upon first definition.

Version 1:

Reviewer comments:

Reviewer #1

(Remarks to the Author)
The revised manuscript has been greatly improved, and the authors have addressed most of my concerns. However, there are still some minor points that need to be corrected.
1. Figure Citations: Please verify and correct erroneous figure panel citations (e.g., Figs. S8C-D and S8E-F).
2. Nomenclature: Please correct the remaining "gH2AX" to "γH2AX" in both the manuscript text and the figures.

Reviewer #2

(Remarks to the Author)
The authors provide satisfactory responses to my concerns regarding the conclusions based on PLA by adding S9.6 immunoprecipitation and PCNA accumulation analyses. Moreover, they demonstrate that the RNAPII inhibitor Triptolide can reduce TRC. In addition, they use mutant isoforms of MLH1 and PMS2 in complementation experiments to better demonstrate the mechanism of action of these two factors in R-loop accumulation and harmful TRC, in coordination with the m5C RNA methyltransferase TRDMT1.

I therefore support publication of this manuscript in Nature Communications.

Reviewer #3

(Remarks to the Author)
Recommendation: Accept in its current form
I have carefully reviewed the revised manuscript and the authors’ point-by-point responses to the reviewers' comments. The authors have substantially strengthened the manuscript through the addition of several new experiments and expanded mechanistic analyses.

In particular, the authors have addressed the major concern regarding the non-canonical role of MutLα in R-loop suppression by demonstrating that both the ATPase activity of MLH1 and the endonuclease activity of PMS2 are required for R-loop removal, suppression of transcription–replication conflicts, maintenance of replication fork progression, and resistance to TRDMT1 inhibition. While direct biochemical reconstitution of MutLα-mediated R-loop processing remains an important future direction, the newly presented genetic and functional evidence significantly strengthens the proposed model.

The authors have also provided important new data implicating EXO1 as a downstream effector of MutLα in R-loop resolution. The epistasis analyses, TRDMT1 inhibitor sensitivity studies, and recruitment experiments collectively support a functional MutLα–EXO1 pathway that suppresses R-loop-associated genomic instability. These findings considerably enhance the mechanistic depth and conceptual impact of the study.

The authors have also adequately addressed the minor concerns regarding nomenclature, figure presentation, data interpretation, and methodological details.

Overall, the revised manuscript presents a compelling and mechanistically well-supported study identifying a therapeutically actionable synthetic lethal interaction between TRDMT1 and MutLα deficiency. The work advances our understanding of how RNA modification pathways cooperate with DNA repair factors to suppress transcription–replication conflicts and maintain genome stability. The findings have clear translational relevance for the treatment of MLH1/PMS2-deficient

cancers.

Open Access This Peer Review File is licensed under a Creative Commons Attribution 4.0 International License, which permits use, sharing, adaptation, distribution and reproduction in any medium or format, as long as you give appropriate credit to the original author(s) and the source, provide a link to the Creative Commons license, and indicate if changes were made.
In cases where reviewers are anonymous, credit should be given to 'Anonymous Referee' and the source.
The images or other third party material in this Peer Review File are included in the article's Creative Commons license, unless indicated otherwise in a credit line to the material. If material is not included in the article's Creative Commons license and your intended use is not permitted by statutory regulation or exceeds the permitted use, you will need to obtain permission directly from the copyright holder.
To view a copy of this license, visit https://creativecommons.org/licenses/by/4.0/

Point-by-point response to reviewers' comments

Reviewer #1 (Remarks to the Author):

This manuscript identifies a novel and potentially therapeutically relevant synthetic lethal interaction between MutLα (MLH1-PMS2) deficiency and inhibition of the RNA methyltransferase TRDMT1. The work compellingly demonstrates that both factors function in parallel to suppress co-transcriptional R-loops, transcription-replication conflicts (TRCs), and associated DNA damage. The in vivo efficacy of a TRDMT1 inhibitor against MLH1-deficient tumors is particularly promising. However, as outlined below, several major conceptual and mechanistic gaps significantly weaken the support for the central claims. Addressing these concerns is essential to establish the proposed model and validate the therapeutic hypothesis.

We thank the reviewer for his/her appreciation of this work and constructive comments. Below, we have carefully addressed each of the comments.

Major Concerns:

1. The Mechanistic Role of MutLα in R-loop Suppression is Undefined. The claim that MutLα suppresses R-loops independently of its canonical MMR role is not substantiated. The manuscript fails to investigate whether known MutLα activities (ATP hydrolysis, endonuclease function) are required for this proposed novel function. Direct experiments are needed to elucidate the mechanism. This may include expressing separation-of-function MutLα mutants (e.g., ATPase-dead, endonuclease-dead) in MLH1-KO cells to test their ability to rescue R-loop accumulation, fork progression defects, and TRDMT1i sensitivity.

The reviewer raised a critical question about the mechanism by which MutLα suppresses R-loops, which we completely agree. To address this comment, we have generated MLH1-G67R, a MLH1 mutant defective for the ATPase activity, and PMS2-E705K, a PMS2 mutant lacking endonuclease activity. We depleted endogenous MLH1 and PMS2 proteins with 3' UTR siRNAs from HeLa cells and expressed the mutants in these cells (**Rebuttal Fig. 1A-B**/revised Fig. S8A). Using the cells expressing the MLH1 and PMS2 mutants, we performed the following experiments to test their effects on R-loops, TRCs, and TRDMT1i sensitivity.

First, we performed DRIP-qPCR to examine the effects of MLH1-G67R and PMS2-E705K on the levels of R-loops (**Rebuttal Fig. 2A-B**/revised Fig. 6A-B). Knockdown of MLH1 or PMS2 increased R-loops at *ACTB* and *RPL13A* genes, and expression of wild-type MLH1 or PMS2 reversed this phenotype. In TRDMT1 knockdown cells, depletion of endogenous MLH1 or PMS2 further increased R-loops, and this phenotype was also suppressed by exogenous MLH1-WT and PMS2-WT. In contrast to their wild-type counterparts, MLH1-G67R and PMS2-E705K failed to suppress R-loops in MLH1 or PMS2 depleted cells, regardless of whether TRDMT1 was knocked down. These results clearly demonstrate that the ATPase and endonuclease activities of MutLα are required for the suppression of R-loops.

Second, we used S9.6:RNAPII p-S2 PLA to analyze TRCs (**Rebuttal Fig. 3A-B**/revised Fig. 6C-D). Knockdown of MLH1 or PMS2 increased PLA foci, and these effects were suppressed by MLH1-WT and PMS2-WT, but not MLH1-G67R and PMS2-E705K mutants. Similar results were

obtained in TRDMT1 knockdown cells. Notably, the PLA foci are removed by RNase H1 expression, confirming the specificity of the PLA assay. These results show that the ATPase and endonuclease activities of MutLα are also required for suppressing TRCs.

Third, we preformed DNA fiber assay to analyze replication forks speed (**Rebuttal Fig. 4A-B**/revised Fig. 6E-F). Knockdown of MLH1 or PMS2 reduced fork speed, and this effect was reversed by exogenous MLH1-WT or PMS2-WT. Consistent results were observed in TRDMT1 knockdown cells. In contrast to WT proteins, MLH1-G67R and PMS2-E705K mutants failed to rescue fork speed, suggesting that the ATPase and endonuclease activities of MutLα are also required for maintaining fork speed in the presence of increased R-loops and TRCs.

Finally, we tested the effects of the mutants on TRDMT1i sensitivity using colony formation assay (**Rebuttal Fig. 5A-B**/revised Fig. 6G-H). Knockdown of MLH1 or PMS2 increased TRDMT1i sensitivity, and this phenotype was reversed by MLH1-WT or PMS2-WT. In contrast, MLH1-G67R and PMS2-E705K mutants failed to restore TRDMT1i resistance.

Collectively, our new results above compellingly demonstrate that MutLα suppresses R-loops and TRCs, enables normal replication fork progression, and prevents TRDMT1i sensitivity by acting as an ATP-dependent endonuclease. These results shed important lights on the mechanism by which MutLα functions as a suppressor of R-loop-associated genomic instability and vulnerability. We thank the reviewer for this constructive comment.

2. The Link Between Genomic Instability, MSI, and TRDMT1i Sensitivity is Ambiguous. The assertion that cancers with "high genomic instability" are sensitive is not quantitatively supported. Since MutLα-deficiency causes MSI, it is critical to distinguish whether TRDMT1i sensitivity is specifically tied to the MSI phenotype, the underlying R-loop burden, or a broader genomic instability signature. Is it possible that the authors could calculate a quantitative genomic instability index (e.g., integrating CIN score, loss of heterozygosity, MSI status) for the cell line panel and correlate it directly with TRDMT1i IC50 values?

This is another excellent suggestion by the reviewer. To address this comment, we used our cell line screening data to analyze the possible association of CIN, aneuploidy, and LOH with TRDMT1i sensitivity (**Rebuttal Fig. 6A-C**/revised Fig. S1I-J). When cell lines were divided into TRDMT1i resistant and highly sensitive groups (see Fig. 1A), the highly sensitive group showed significantly higher CIN and Aneuploidy scores. Furthermore, the TRDMT1i highly sensitive group also displayed significantly higher LOH fraction. These results show that high TRDMT1i sensitivity is generally associated with high chromosomal instability.

In our original manuscript, we have already shown that the MSI status is not associated with TRDMT1i sensitivity (Fig. S2A). This is because the MSI of cancer cells is caused by not only MLH1 and PMS2 mutations, but also MSH2/3/6 and PMS1 mutations. As we showed, the latter group of MMR mutants do not increase TRDMT1i sensitivity, though they can still cause MSI. Notably, when we specifically analyzed the MSI cell lines, mutations or low expression of MLH1, which cause MSI in a subset of these cell lines, are associated with high TRDMT1i sensitivity (Fig. 1C). This result shows that loss of MLH1 but not all MMR genes is associated with TRDMT1i sensitivity, and that MSI alone is not a good predictor of TRDMT1i sensitivity.

3. Lack of Evidence that R-loop Accumulation is the Primary Lethal Driver. This is a critical omission. If R-loop accumulation is the primary driver of synthetic lethality, the authors should demonstrate that RNase H1 overexpression rescues not only cell viability but also DNA damage (e.g., γH2AX) in TRDMT1i-treated, MutLα-deficient cells. Without this key experiment, the causal chain remains speculative.

Please note that we have already demonstrated in our original manuscript that RNase H1 expression rescued cell viability in TRDMT1i-treated, MutLα-deficient cells (Fig. 4E and 4F). To extend these experiments as the reviewer suggested, we analyzed the levels of γH2AX in these cells (**Rebuttal Fig. 7**/revised Fig. S4A). Knockdown of MLH1 or TRDMT1i treatment alone increased γH2AX foci, and the combination increased γH2AX foci further. Importantly, these γH2AX foci were drastically reduced by RNase H1 expression. These results show that the TRDMT1i-induced DNA damage and synthetic lethality in MLH1-deficient cells are indeed driven by R-loops.

4. The Mechanism of Cell Death is Not Investigated. The cellular consequence of TRDMT1 inhibition in MutLα-deficient cells (apoptosis, necrosis, mitotic catastrophe, senescence) is entirely undefined. Characterizing the mode of cell death using standard assays is essential for understanding the therapeutic outcome.

To address this comment, we analyzed markers of cell death using both Western blot and FACS (**Rebuttal Fig. 8, 9A-B**/revised Fig. S4B-D). Using western blot, we found that cleaved PARP and Caspase 3 were induced by the combination of siMLH1 and TRDMT1i, but not by siMLH1 or TRDMT1i alone. In addition, using Annexin V-PI staining and FACS, we found that the combination of siMLH1 and TRDMT1i substantially increased the fraction of Annexin V and PI double-positive cells. These results demonstrate that TRDMT1i induces apoptosis in MLH1-defiicent cells.

5. The Contribution of Endogenous TRDMT1 Expression Levels is Unclear. Since high TRDMT1 expression correlates with inhibitor sensitivity, it is vital to rule out that the observed synthetic lethality is not confounded by higher basal TRDMT1 expression/activity in the MLH1-deficient models used. The authors should quantify and report TRDMT1 mRNA and protein levels in the isogenic cell pairs (MLH1 WT vs. KO) and perform a correlation analysis of MLH1 and TRDMT1 expression across the broader cell line panel.

We would like to clarify our interpretation of the relationship between TRDMT1 expression levels and TRDMT1i sensitivity. In a large panel of cancer cell lines, high TRDMT1 expression is associated with high TRDMT1i sensitivity (Fig. S1A). We think this reflects that TRDMT1 is up regulated in cancer cells where the dependency on TRDMT1 is increased – a result of selection for cell fitness. Therefore, high TRDMT2 expression is generally associated with increased TRDMT1 sensitivity in cancer cells, which presumably includes MLH1-deficient cancer cells. Therefore, even if TRDMT1 expression/activity is increased in MLH1-deficient cells, it is not a "confounding" factor, but rather a line of evidence for TRDMT1 dependency.

Minor Concerns:

1. Discrepancy in PLA Foci Detection: The absence of baseline S9.6:PCNA PLA foci in in vivo MLH1-KO tumors (Fig. 6F) compared to in vitro data (Ext. Data Fig. 3G) needs explanation. Comment on assay sensitivity or potential in vivo compensation.

Indeed, the sensitivity of PLA could be different in cultured cells and in tumor sections. Also, the baseline TRCs in tumors may be different due to compensatory mechanisms. However, we would like to point out that a baseline level of S9.6:PCNA PLA foci was observed in MLH1-KO tumors in vivo (~3-5%, see revised Fig. 7F), which is similar to that observed in vitro (revised Fig. 3F-G). Therefore, we do not think that there is a discrepancy between the in vivo and in vitro results.

2. Nomenclature: Correct "gH2AX" to "γH2AX" throughout the manuscript and figures.

These errors have been corrected.

3. Figure Citations: Verify and correct erroneous figure panel citations (e.g., Page 11 reference to Ext. Data Fig. 5c).

Thank you for spotting these errors. All of these errors have been corrected.

4. Typographical Error: Confirm the cell concentration for the DNA fiber assay ("200 cells/mL" is likely incorrect).

ML should be ul. This error has been corrected.

5. Gene Nomenclature: Use correct mouse gene symbols (e.g., Mlh1-KO CT26, not MLH1-KO CT26).

This nomenclature error has been corrected.

6. Data Interpretation: Re-evaluate and clarify the description of the data in Fig. 3B, particularly regarding the ILF2 locus, to ensure accuracy.

We thank that reviewer for this thoughtful comment. In this panel (Fig. 3B), the DRIP-qPCR results show that TRDMT1 but not MLH1 is the major suppressor of R-loops at two m5C-positive genes (*EEF2* and *ILF2*), supporting the idea that TRDMT1 suppresses R-loops by generating m5C. However, at two other genes where m5C was not detected (*ACTB* and *RPL13A*), both TRDMT1 and MLH1 contributed to R-loop suppression. We interpret that the levels of m5C at these genes are lower (below the threshold for reliable detection by bisulfide sequencing), thereby limiting the R-loop suppressor function of TRDMT1 at these genes. Consequently, the R-loop suppressor function of MLH1, which is not dependent on m5C, is increased at these genes. We confirm that our interpretation is clearly explained.

7. Figure Quality: Provide a cleaner western blot for Fig. 5D to unequivocally demonstrate the specific MLH1-PMS2 interaction.

We have repeated the experiment and provided cleaner Western blots in the revised figure (**Rebuttal Fig. 10**/revised Fig. 5D).

8. In Vivo Dosing: Justify the high doses of TRDMT1i used in vivo (100-200 mg/kg) with references or discuss potential toxicity.

In our unpublished studies, we have checked the general toxicity of TRDMT1i in mice. Daily treatments of 5 mice with TRDMT1i at 200 mg/kg by gavage did not reduce body weight significantly over 14 days (**Rebuttal Fig. 11**), suggesting that this dose of TRDMT1i is well tolerated in vivo.

Reviewer #2 (Remarks to the Author):

This is an excellent study on new roles of MutLa mismatch repair complex in coordination with the m5C RNA methyla trasferase TRDMT1. The study uncover a cooperative functions of these two factors to suppress co-transcriptional R-loops accumulation and prevent harmful transcription–replication conflicts. Loss of either pathway slows DNA replication, but combined loss causes more severe replication fork stalling, R-loop accumulation, and synthetic lethality, Importantly, cancer cells lacking MLH1 or PMS2 are highly dependent on TRDMT1, and pharmacological inhibition of TRDMT1 selectively impairs growth of MLH1-deficient tumors in vivo. This work is relevant because it expands on the idea that R-loop are regulated by RNA methylation–and proposes TRDMT1 inhibition as a targeted therapeutic strategy for MutLα-deficient cancers. I globally support pubblication of this study in Nature Comm. I only have few suggestions

We thank the reviewer for his/her support!

1) My main concern is on the extensive use of PLA assay for RNApII and PCNA as a read out of TRC. Despite PLA is a powerfull approach, it can be prone to folse positive results due to interaction amonge the relevant factors elsewhere in the nuceloplasms on on chromatin. PLA results has been coupled with ChIP -qPCR for either PCNA and RNA ppoIII in the different conditions, since CUT&TAG approach in the study identify the relevat genomic context in which TRC likely occurs. I believe it would be reassuring to complement main PLA results with more standard appraches like Co-IP RNA immunoprecipitaion or DRIP.

To address this comment, we have performed S9.6 immunoprecipitation to capture chromatin fragments containing R-loops (**Rebuttal Fig. 12**/revised Fig. S3H). Subsequently, we analyzed the R-loop-containing chromatin by Western blot with anti-PCNA antibody to detect any replication forks on the fragments. Our results show that PCNA was detected on the chromatin fragments captured by S9.6, suggesting that some R-loops and replication forks are indeed in close

proximity on chromatin in the presence of baseline TRCs. In cells treated with siTRDMT1 and siMLH1, PCNA levels on S9.6-captured chromatin were increased, suggesting that the levels of TRCs are increased by loss of TRDMT1 and MutLα. Importantly, PCNA was not detected on S9.6-captured chromatin when RNaseH1 was overexpressed in cells, confirming that the chromatin capture by S9.6 is R-loop-dependent. Together, this new experiment lends further support to the point that concurrent loss of TRDMT1 and MLH1 substantially increases TRCs.

2) Suppression of TRC with a treatment of any RNApolII inhibitor (DRB for example) could be informative in the different condition also to undestand if R-loops form also involving pre existing transcris.

To address this comment, we have tested the effects of the RNAPII inhibitor Triptolide on TRCs (**Rebuttal Fig. 13**/revised Fig. S3G). Using PNAPII:PCNA PLA, we confirmed that knockdown of TRDMT1 or MLH1 alone increased TRCs, and double knockdown increased TRCs further. Importantly, a brief Triptolide pre-treatment of cells (4 hours) substantially reduced PLA foci, suggesting that TRCs are dependent on pre-existing transcription (and presumably transcripts).

Reviewer #3 (Remarks to the Author):

This study identifies a synthetic-lethal interaction between the RNA methyltransferase TRDMT1 and the mismatch repair complex MutLα (MLH1–PMS2), independent of canonical mismatch repair. Through a large-scale cancer cell line screen, the authors show that MLH1 or PMS2 loss confers strong sensitivity to a first-in-class TRDMT1 inhibitor, driven by excessive R-loop accumulation, transcription–replication conflicts (TRCs), and replication fork slowing. Mechanistically, TRDMT1 and MutLα independently recognize RNA–DNA hybrids and suppress overlapping but distinct subsets of R-loops, with RNA m5C modification dictating pathway choice. Importantly, TRDMT1 inhibition selectively suppresses MLH1-deficient tumor growth in vivo by inducing R-loop–mediated replication stress.

Overall, the manuscript is mechanistically sound, and clinically relevant, well suited for Nature Communications. The core conclusions are generally well supported, and the work advances understanding of how RNA modifications and DNA repair factors cooperate to suppress transcription–replication conflicts. Addressing the biochemical mechanism of non-canonical role of MutLα at R-loops would further elevate the impact, but even in its current form, the study findings are original and represents a major conceptual advance in the field. The use of multiple orthogonal approaches to detect R-loops (PLA, GFP-dRNaseH1 staining, DRIP-qPCR, CUT&Tag), combined with functional rescue by RNaseH1, provides compelling evidence that R-loop accumulation is causal for TRCs, fork slowing, and synthetic lethality. Experimental approaches are appropriate, well controlled, and described in sufficient detail to allow reproduction. The integration of cell-based assays, genome-wide profiling, and in vivo tumor models strengthens the overall rigor of the study.

We thank the reviewer for his/her appreciation of our work.

Points for Improvement and Clarification:

There are some areas that could be improved to further enhance mechanistic clarity and impact.
1. Direct biochemical activity of MutLα at R-loops. The data convincingly show recruitment and functional relevance of MutLα at R-loops, but direct biochemical evidence demonstrating whether MutLα cleaves RNA–DNA hybrids or displaced ssDNA strands is lacking. If testing this experimentally is technically not feasible, a discussion about its possible mechanistic role should be included.

We completely agree with the reviewer that direct biochemical evidence demonstrating the activity of MutLα in removing R-loops is critical for solidifying our model. However, as the reviewer also alluded to, biochemical reconstitution of this process is challenging, especially when we still don't know all the proteins involved. To strengthen our model and provide more mechanistic details, we have generated and tested an ATPase-deficient MLH1 mutant and an endonuclease-deficient PMS2 mutant (**Rebuttal Fig. 1A-B**/revised Fig. S8A). Using cells in which endogenous MLH1/PMS were replaced by these mutants, we showed that the ATPase and endonuclease activities of MutLα are required for removing R-loops (**Rebuttal Fig. 2A-B**/revised Fig. 6A-B), suppressing TRCs (**Rebuttal Fig. 3A-B**/revised Fig. 6C-D), maintaining replication fork speed (**Rebuttal Fig. Fig. 4A-B**/revised Fig. 6E-F), and preventing TRDMT1i sensitivity (**Rebuttal Fig. 4A-B**/revised Fig. 6G-H). Collectively, these new results strongly suggest that MutLα acts as an ATP-dependent endonuclease to suppress R-loop-associated genomic instability by cleaving and removing R-loops. In addition, our new results show that MutLα recruits EXO1 to R-loops, where EXO1 functions with MutLα in concert to remove R-loops (see below). Based on these new findings, we have revised the Discussion section to include a detailed discussion on the possible mechanisms by which MutLα and EXO1 cleave and remove R-loops.

2. Downstream R-loop resolution machinery. The manuscript does not identify which helicases or nucleases act downstream of MutLα-mediated R-loop recognition or incision. Genetic or epistasis analyses with known R-loop regulators (e.g., SETX) would add depth.

This is a great suggestion. The reviewer is right that the removal of R-loops ultimately requires one or more helicases or nuclease. Our new results show that the endonuclease activity of MutLα is required for removing R-loops. In the context of MMR, MutLα nicks DNA at mismatches, but is not sufficient for removing the DNA bases causing mismatches. The completion of MMR also requires EXO1, which generates ssDNA gaps at the mismatches, allowing removal of incorrect nucleotides and synthesis of DNA free of mismatches. Based on this corporative relationship between MutLα and EXO1 in MMR, we asked if EXO1 is also involved in the removal of R-loops.

We first asked if low EXO1 expression in cancer cell lines also correlates with TRDMT1i sensitivity as in cells deficient for MLH1 and PMS1 (**Rebuttal Fig. 14A-B**/revised Fig. S9A-B). Indeed, in a large panel of cancer cell lines, EXO1 expression <u>positively</u> correlates with TRDMT1i IC50, suggesting that low EXO1 expression is associated with high TRDMT1i sensitivity. In addition, when cell lines are divided into TRDMT1i resistant and highly sensitive groups (see Fig. 1A), the highly sensitive groups displays lower EXO1 expression, again linking low EXO1 to high TRDMT1i

sensitivity. These results suggest that EXO1 may be involved in the same pathway as MutLα to suppress R-loops and TRDMT1i sensitivity.

Next, we directly tested if EXO1 is required for R-loop suppression (**Rebuttal Fig. 15A-B**/revised Fig. 6I, S9C-D). We knocked down EXO1 with siRNA and measure R-loop levels with GFP-dRNH1 staining. In addition to knocking down EXO1 alone, we knocked down MLH1 and TRDMT1 alone, or co-depleted EXO1/TRDMT1, EXO1/MLH1, and TRDMT1/MLH1. Similar to MLH1 knockdown, EXO1 knockdown also increased R-loops, and EXO1 knockdown in TRDMT1-depleted cells further increased R-loops. Importantly, co-depletion of EXO1 and MLH1 did not increase R-loops further compared to single knockdowns, suggesting that EXO1 and MLH1 are epistatic in R-loop suppression.

To confirm the functional relationship between MLH1 and EXO1, we compared the TRDMT1i sensitivity of MLH1 knockdown cells, EXO1 knockdown cells, and MLH1/EXO1 double knockdown cells (**Rebuttal Fig. 16**/revised Fig. S9E). MLH1 knockdown cells and EXO1 knockdown cells are similarly sensitive to TRDMT1i, and MLH1/EXO1 double knockdown cells does not increase TRDMT1i sensitivity. Together, these new results strongly suggest that MutLα and EXO1 function together to remove R-loops.

Finally, we asked if EXO1 is recruited to R-loops by MutLα (**Rebuttal Fig. 17**/revised Fig. 6J). Using the TA-KR assay, we found that GFP-EXO1 is recruited to the locus of TA-KR-induced DNA damage and R-loops, which is similar to MLH1. Importantly, the localization of EXO1 was significantly reduced by knockdown of MLH1 or PMS2, showing that EXO1 is recruited to sites of R-loops by MutLα.

Based on these new results, we propose that MutLα nicks DNA at R-loops and provides an entry point for EXO1 to remove the DNA strand in DNA-RNA hybrids. This two-step processing event not only removes DNA-RNA hybrids, but also generates ssDNA gaps that can be subsequently filled by other repair proteins – a model sharing similarity to that of MMR. We plan to test this hypothesis using a biochemical approach in a follow-up study.

3. Mechanism of TRDMT1-mediated R-loop suppression in undamaged cells. While prior work implicates FMRP/TET1 at DNA damage sites, the identity of m5C “readers” or effectors operating at co-transcriptional R-loops in unperturbed cells remains speculative. Even limited exploratory data would strengthen this aspect.

We thank the reviewer for this interesting question. Indeed, our prior work suggested that FMRP/TET1 promote the “erasing” of m5C at DNA damage sites, which is necessary for the completion of DNA repair. To address the reviewer’s comment, we have knocked down FMRP using siRNA and analyzed its effects on co-transcriptional R-loops using DRIP-qPCR (**Rebuttal Fig. 18A-B**). Our results show that R-loop levels were reduced at two R-loop-positive genes overlapping with m5C sites (*EEF2* and *ILF2*), but no change was detected in the “noise” level of R-loops at a locus that is not actively transcribed (*NSUN5*). These results are consistent with the idea that m5C promotes R-loop suppression or resolution. In the absence of FMRP, m5C is expected to increase and/or become more persistent, which leads to more efficient removal of R-loops.

Addressing the mechanistic points above experimentally where possible but in text otherwise would further strengthen the manuscript.

Minor Points:

4. There are several instances of inconsistent terminology (e.g., MutLa, MutLα, MHL1 instead of MLH1 in a few figure labels/text passages) that should be standardized throughout the manuscript.

We use MLH1 to specifically describe the MLH1 protein and MutLα to describe the MLH1-PMS2 complex or functions/properties of this complex. We have corrected all MutLa to MutLα.

5. In figure 5, the zoomed insets of IF images do not seem to show any added details. Either show more enlarged images or delete the zoomed boxes.

We have removed the zoomed boxes as suggested.

6. Minor typographical errors are present, such as occasional misspellings (e.g., “TRMT1” instead of “TRDMT1,” “PNCA” instead of “PCNA”) and missing or extra spaces in gene/protein names.

These errors have been corrected.

7. Some sentences in the Introduction and Discussion are overly long and dense and would benefit from light editing for clarity and readability.

Following this suggestion, we have shortened several sentences to improve clarify.

8. Figure legends occasionally repeat information already provided in the main text and could be streamlined to improve clarity.

Follow this suggestion, we have removed redundant information from figure legends.

9. Ensure consistent gene/protein nomenclature (italicized gene names vs. non-italicized proteins) and uniform use of abbreviations upon first definition.

We have checked the use of italicized and non-italicized fonts in gene/protein names in the text and figures and definitions for abbreviations, and corrected a number of errors.

Rebuttal Fig. 1

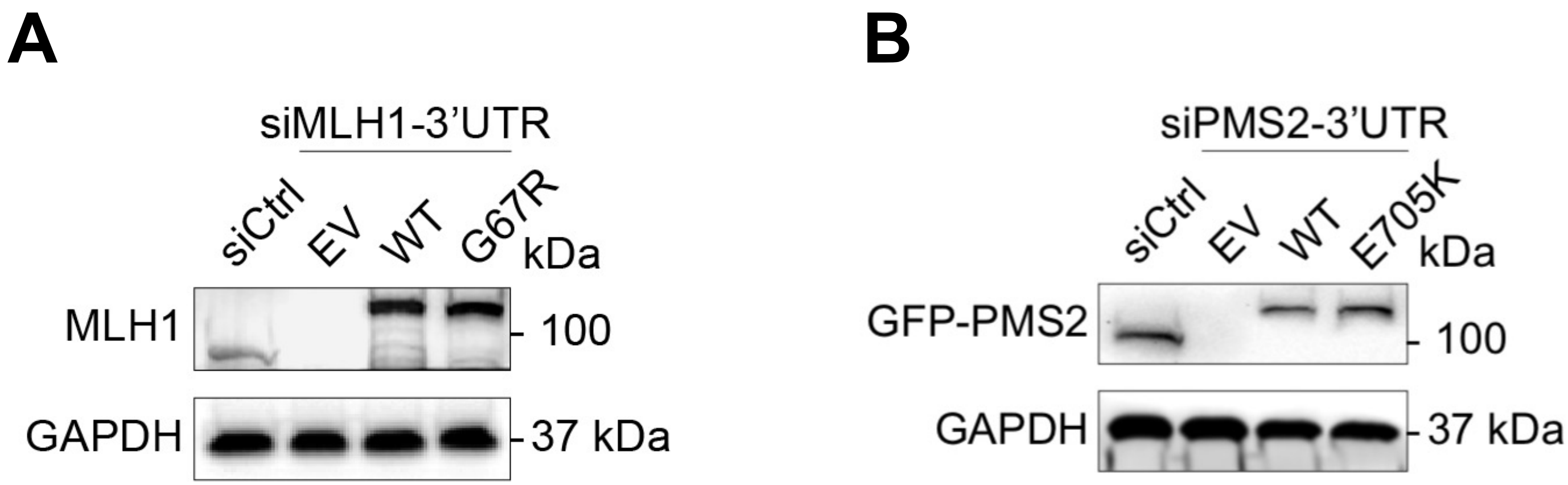

Rebuttal Fig. 2

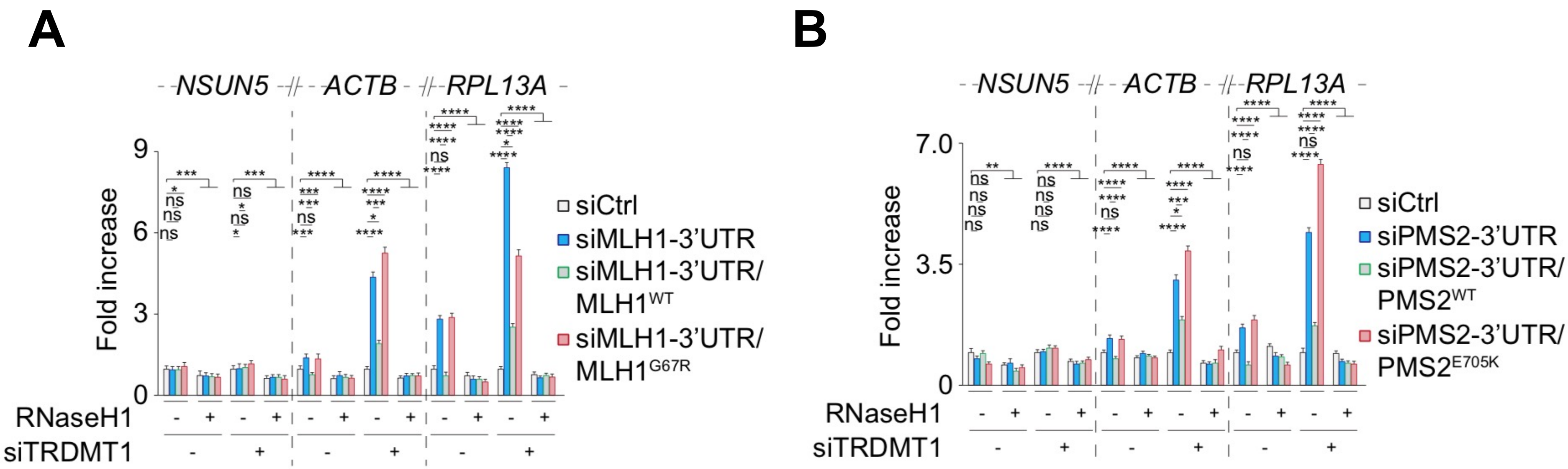

Rebuttal Fig. 3

A

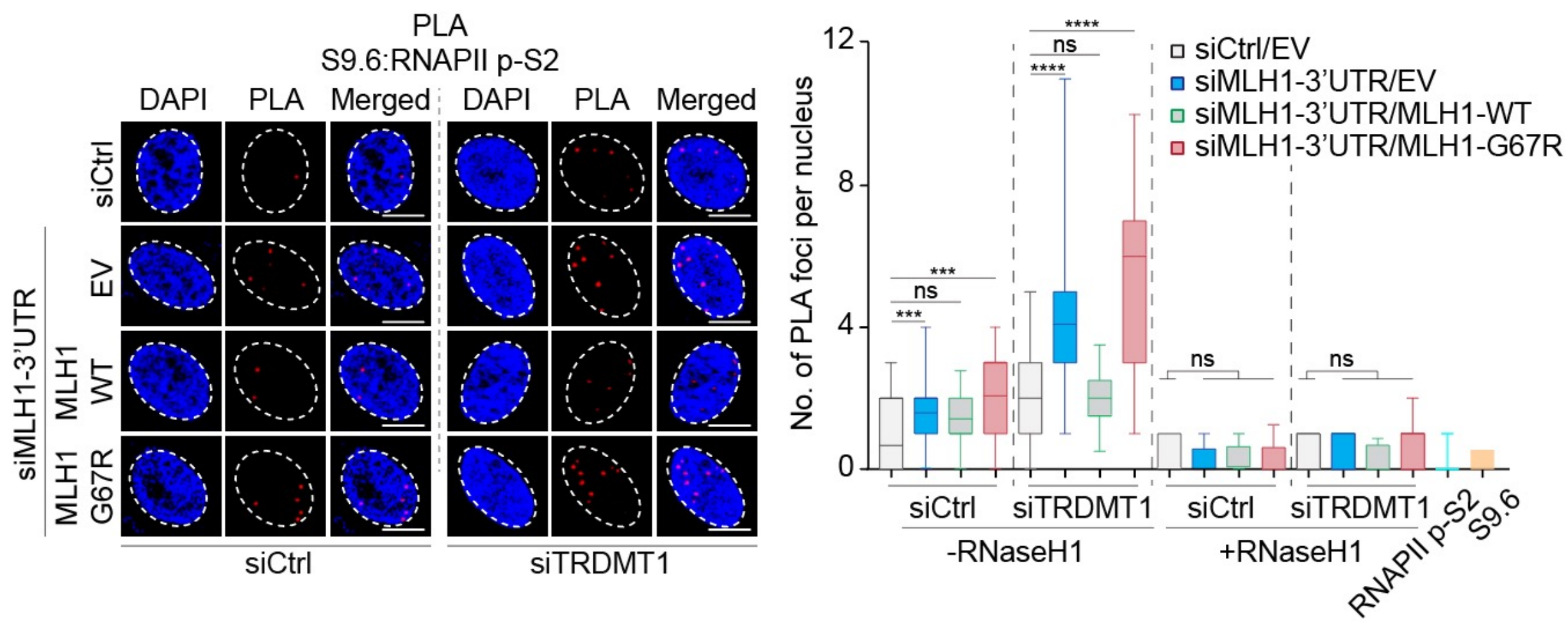

B

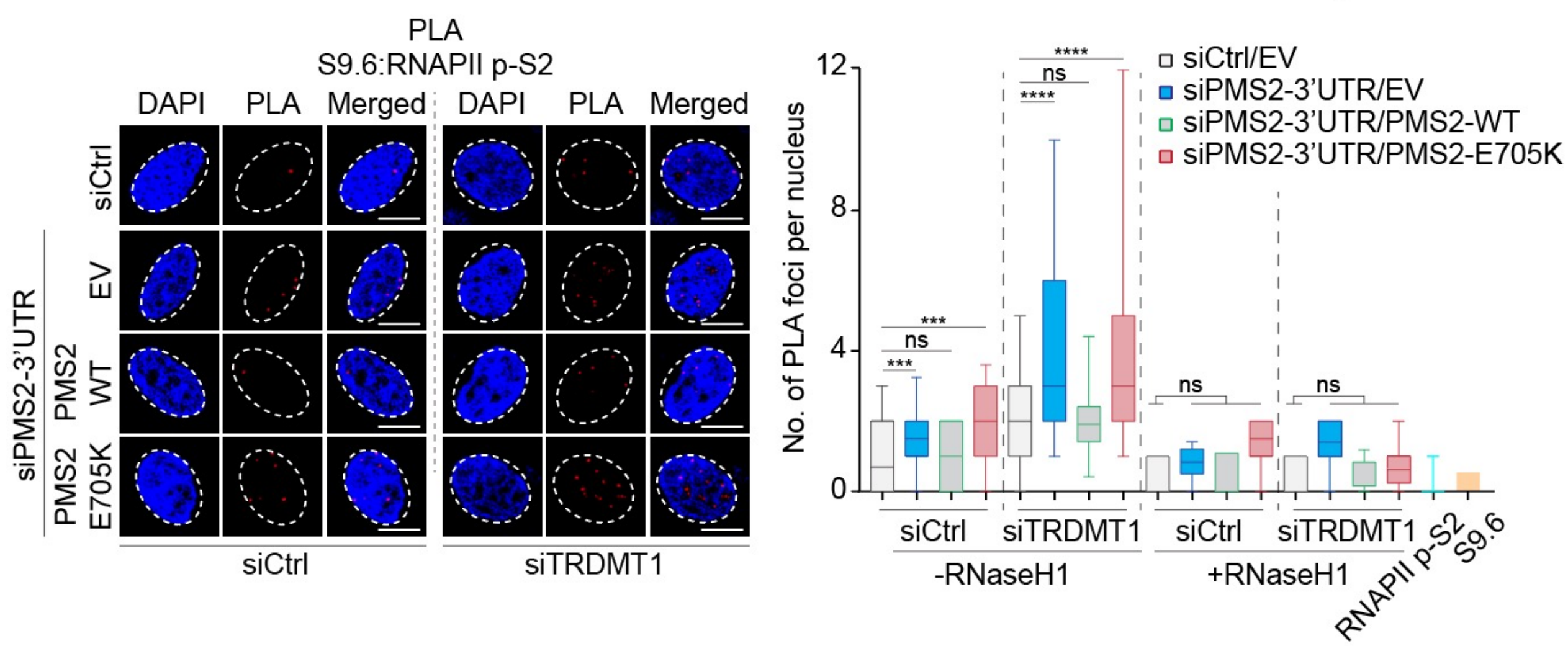

Rebuttal Fig. 4

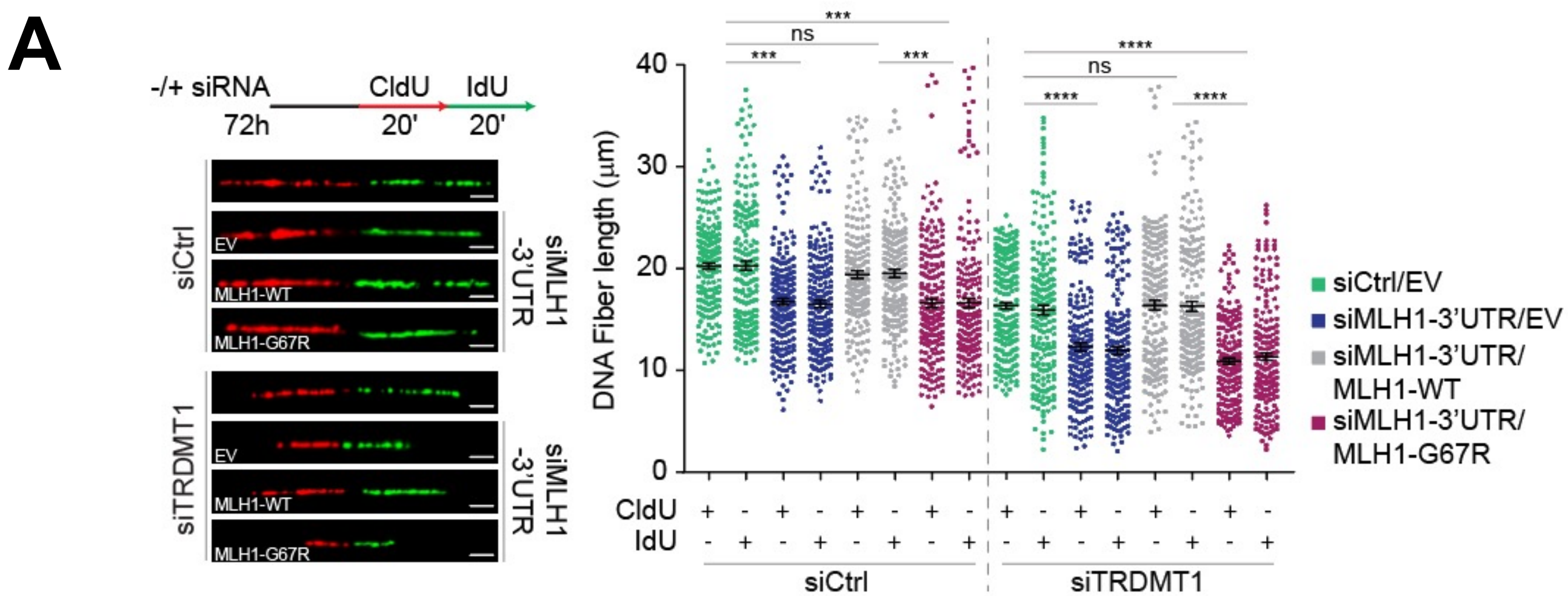

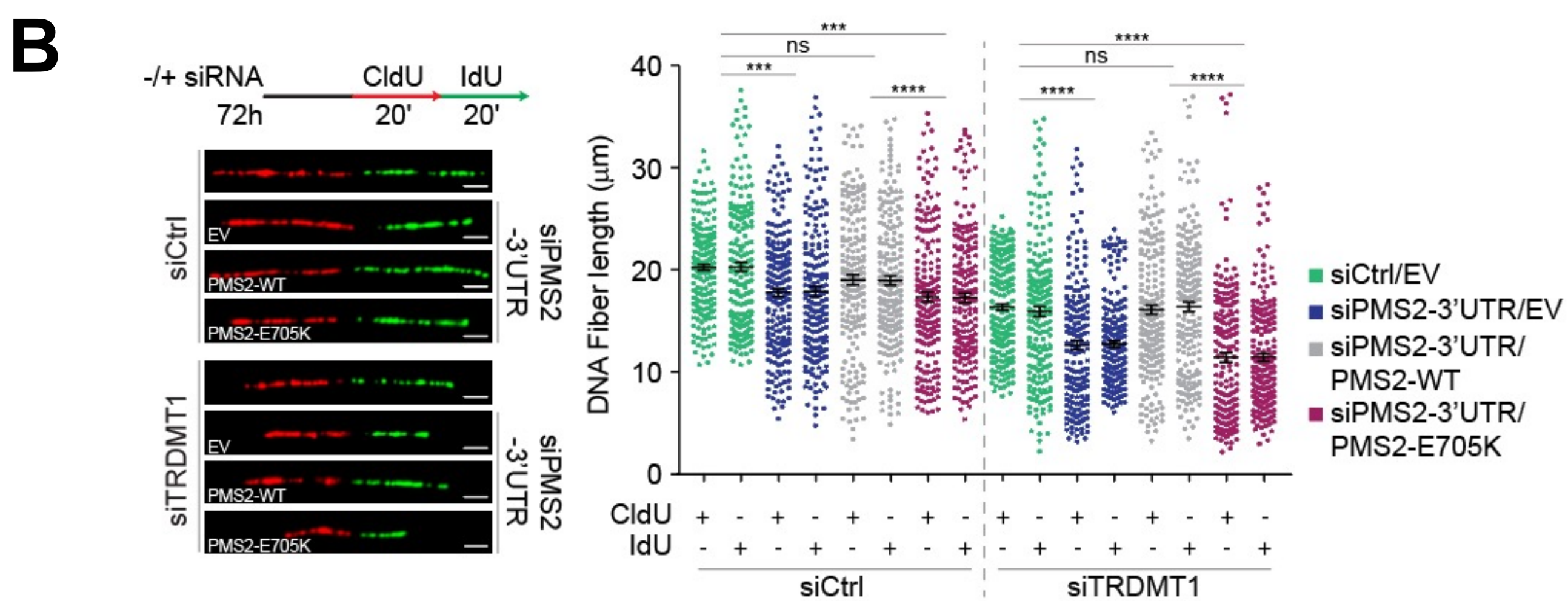

Rebuttal Fig. 5

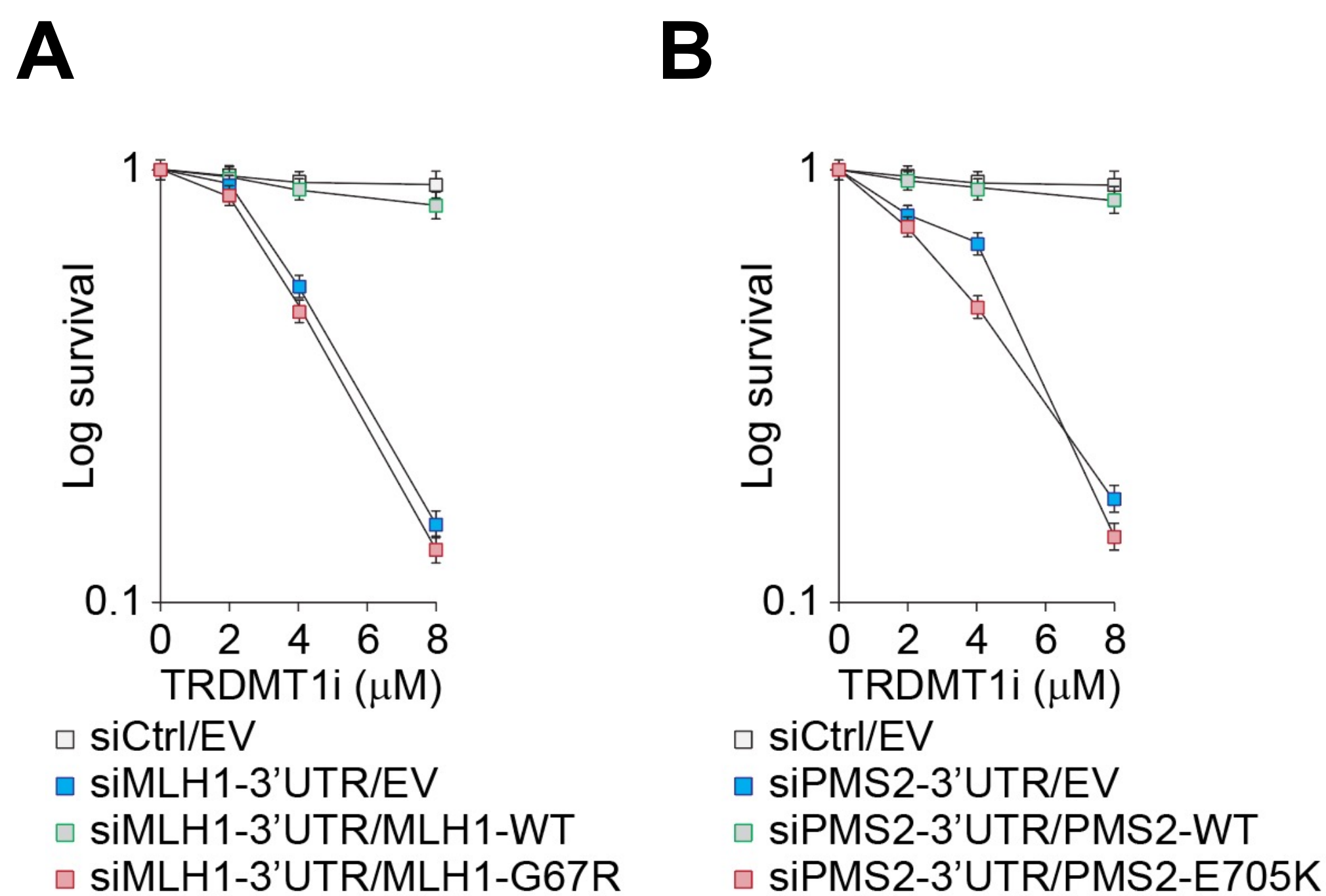

Rebuttal Fig. 6

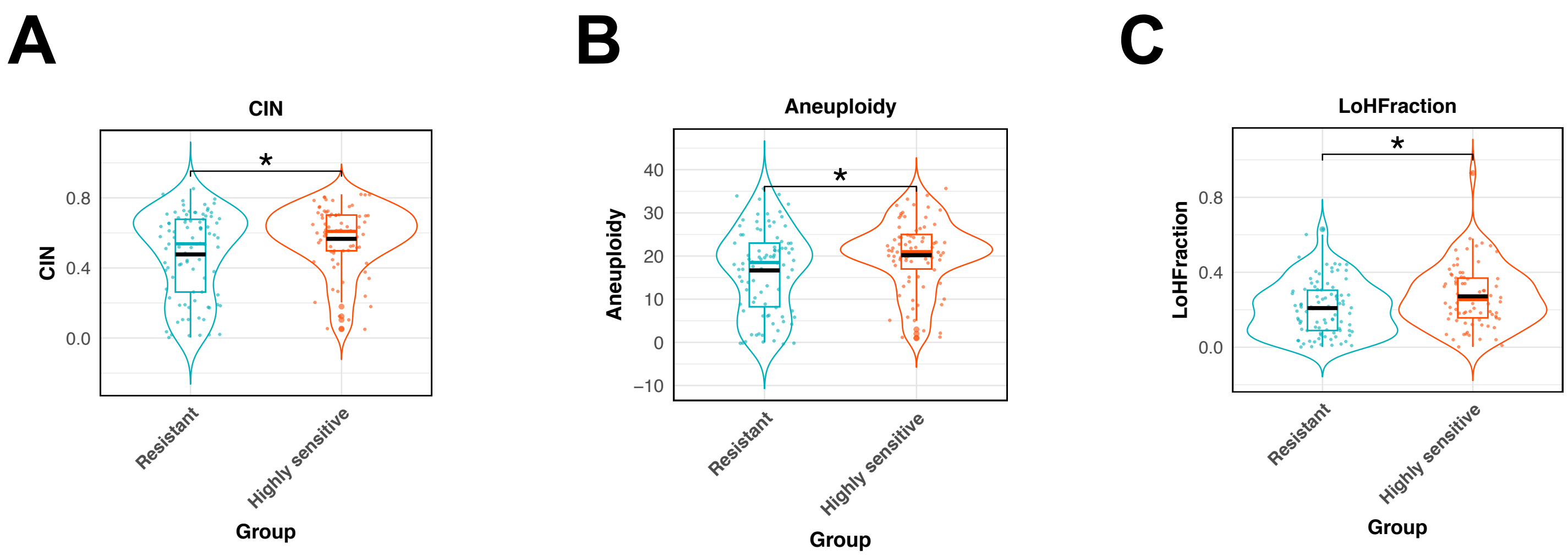

Rebuttal Fig. 7

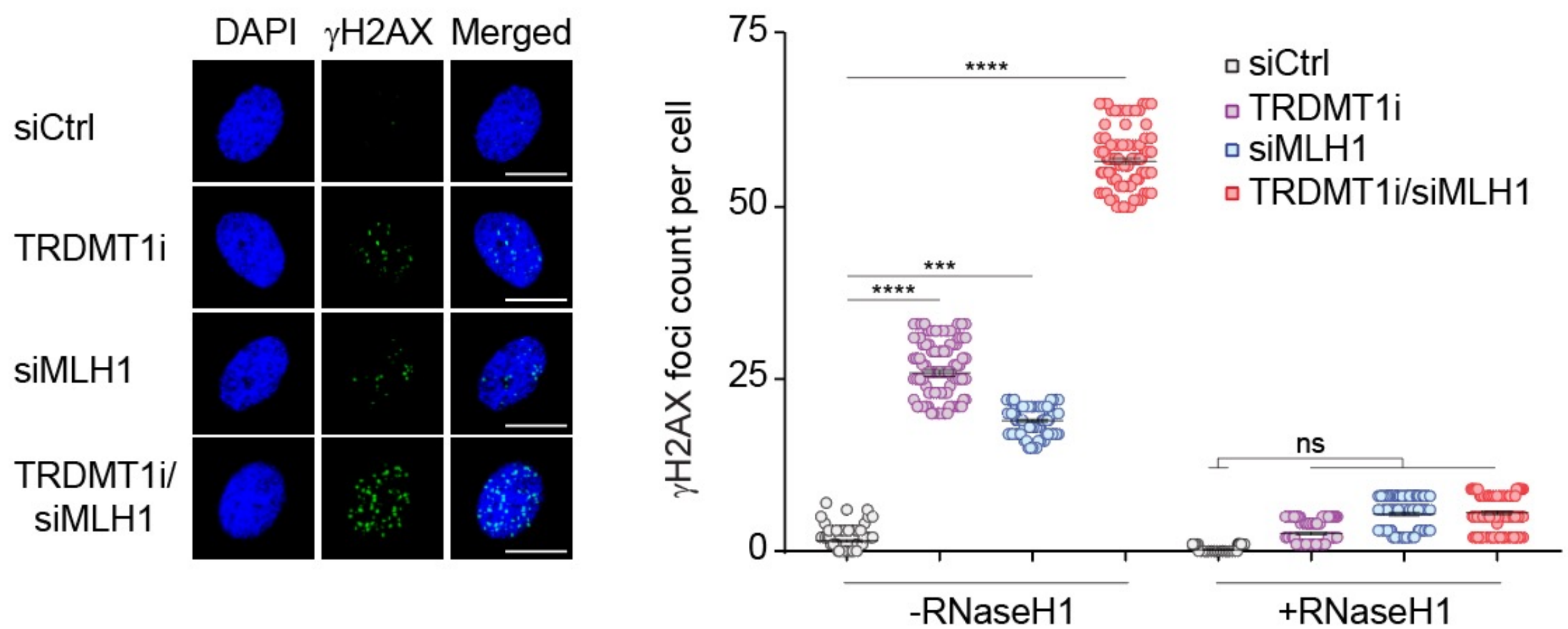

Rebuttal Fig. 8

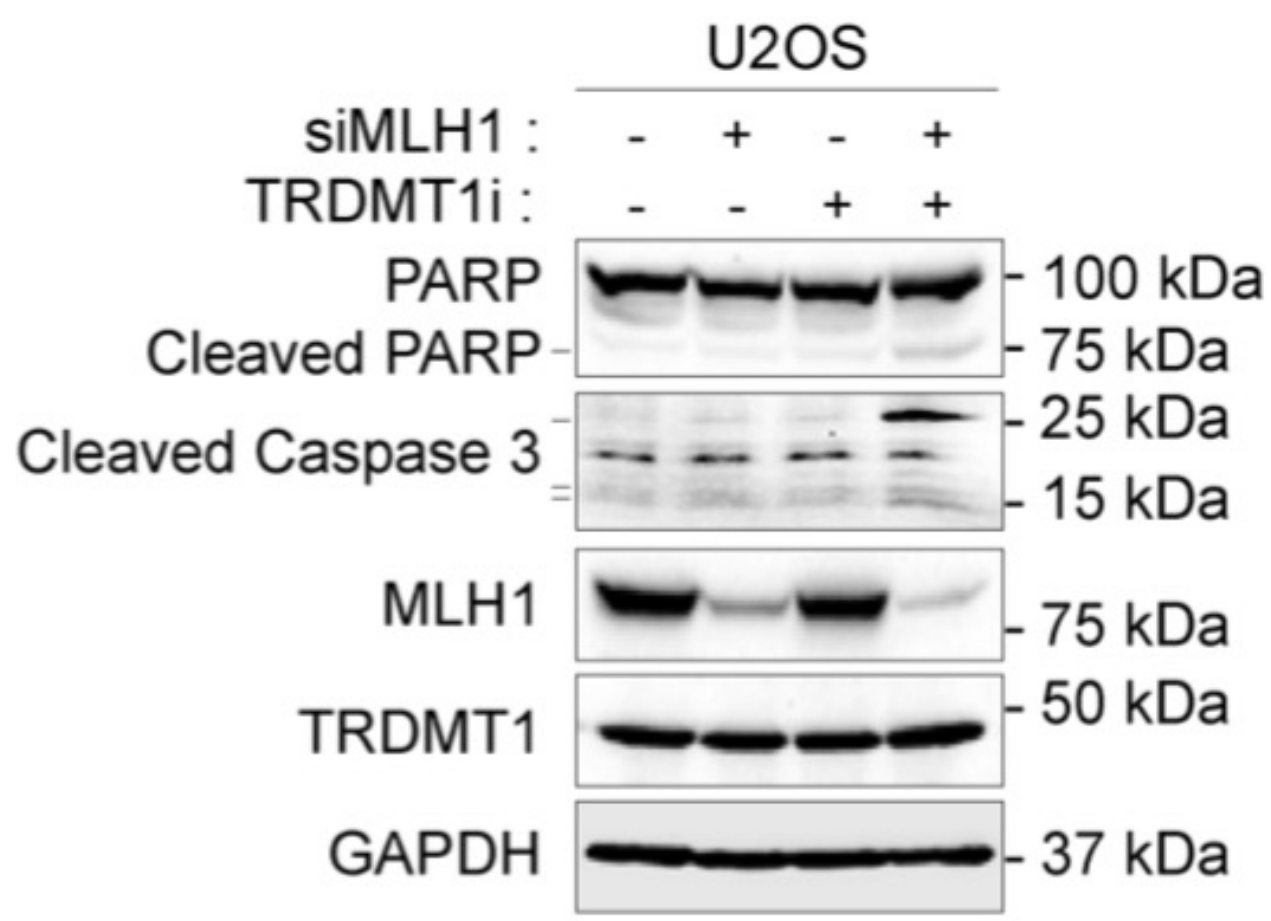

Rebuttal Fig. 9

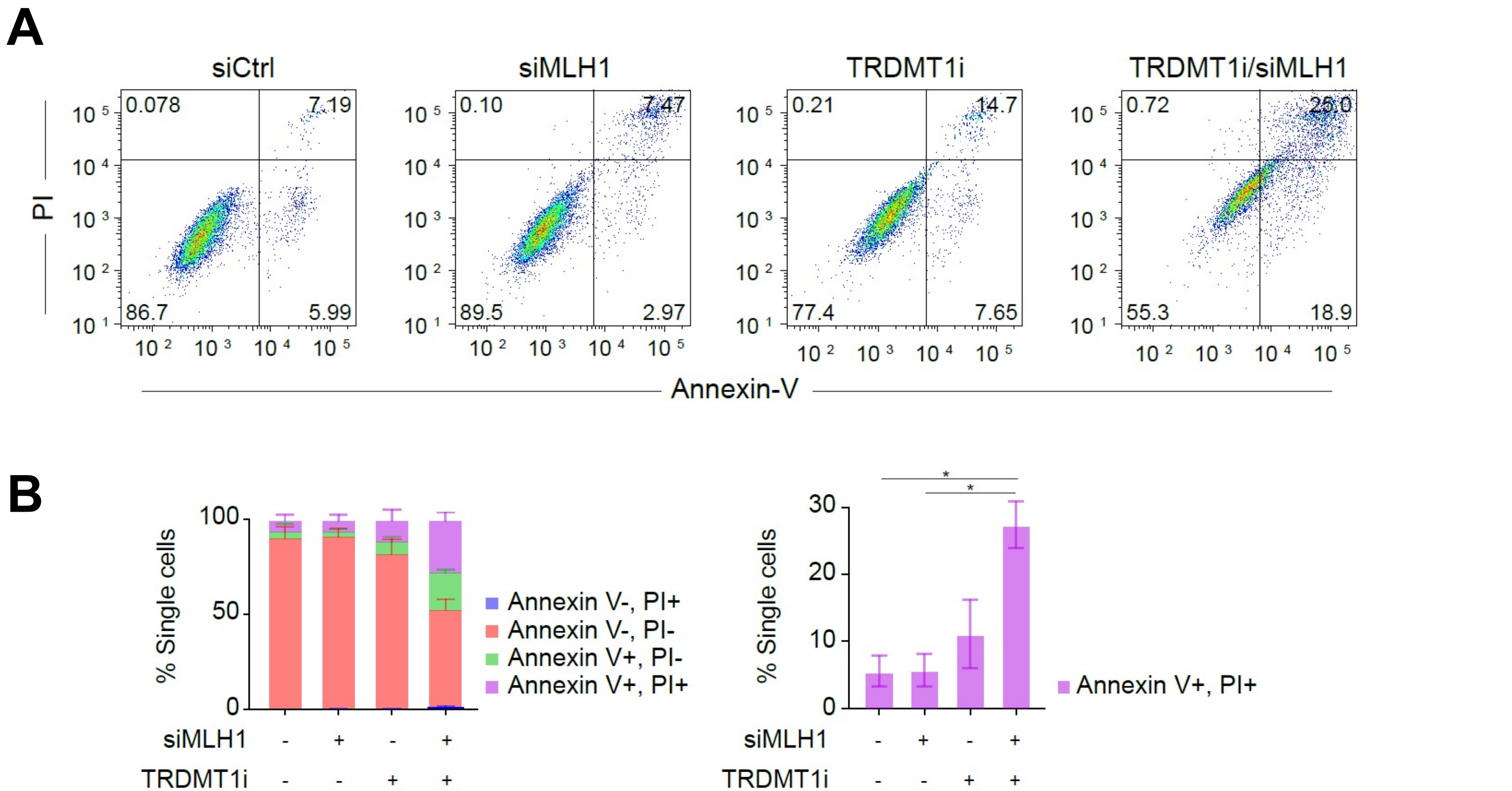

Rebuttal Fig. 10

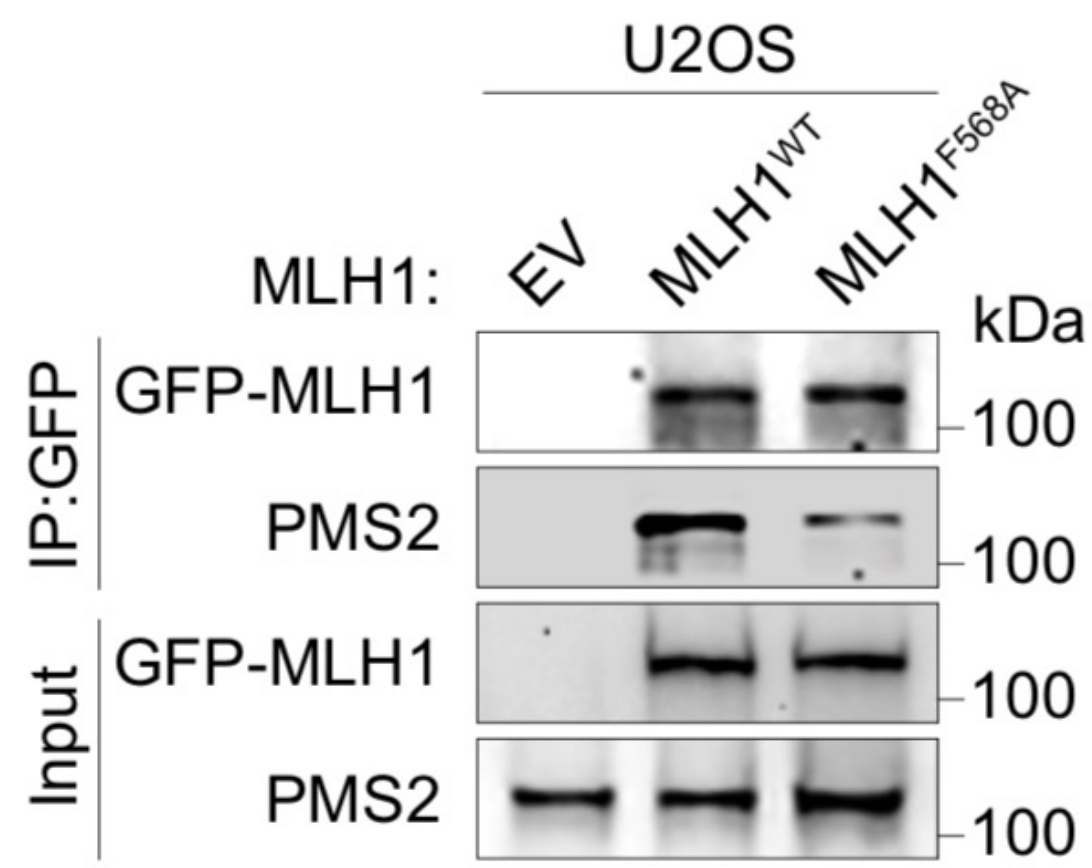

Rebuttal Fig. 11

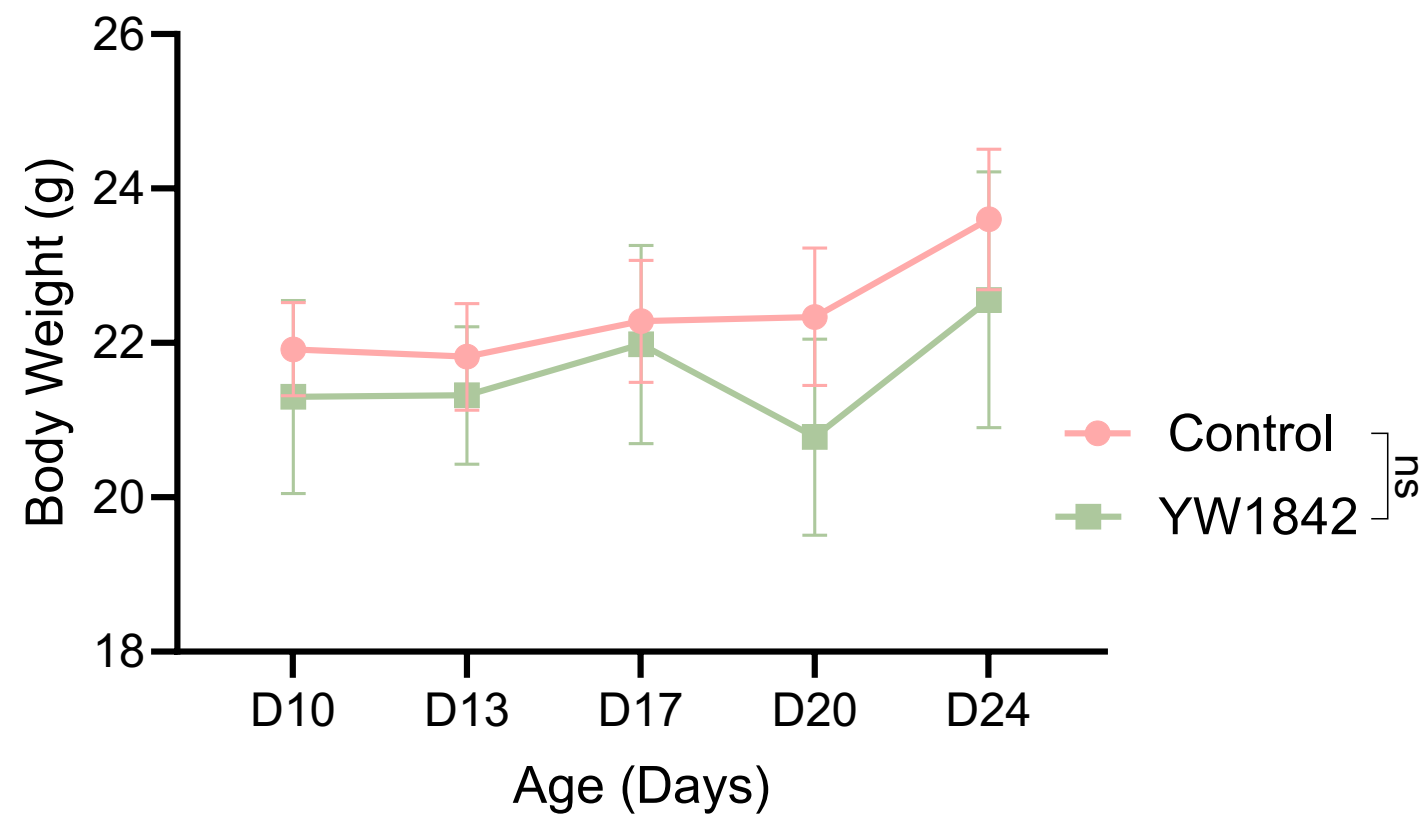

Rebuttal Fig. 12

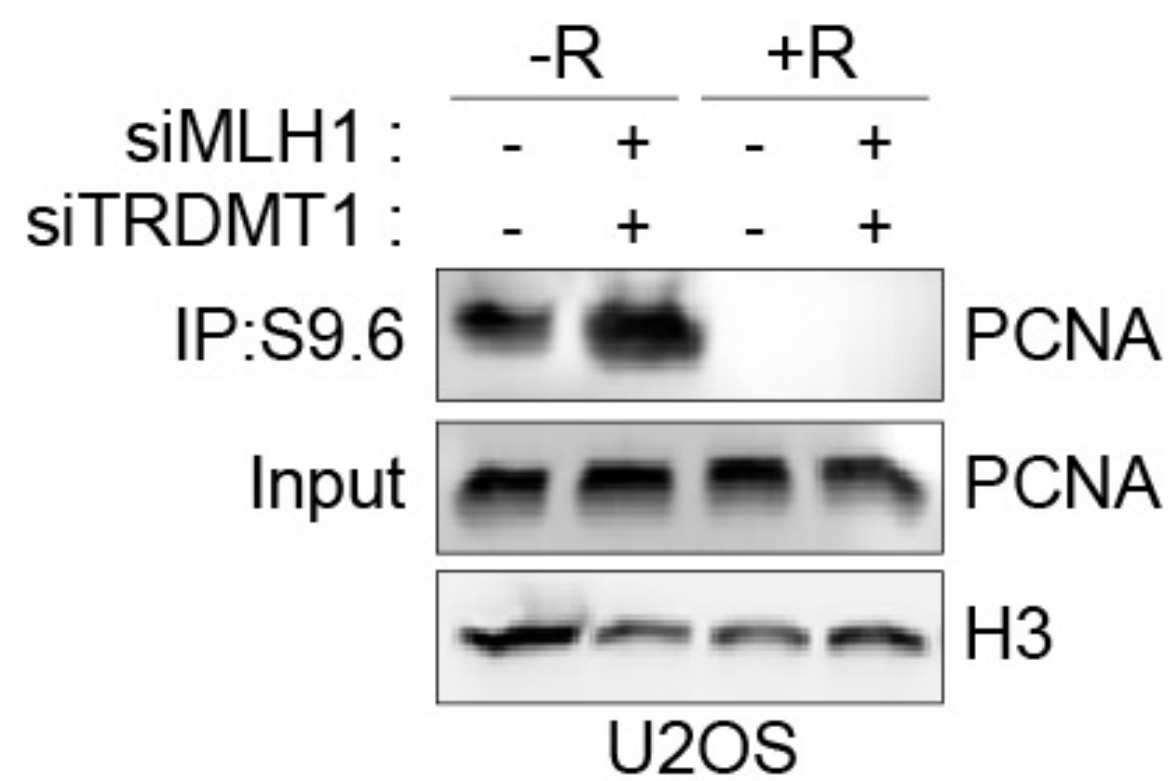

Rebuttal Fig. 13

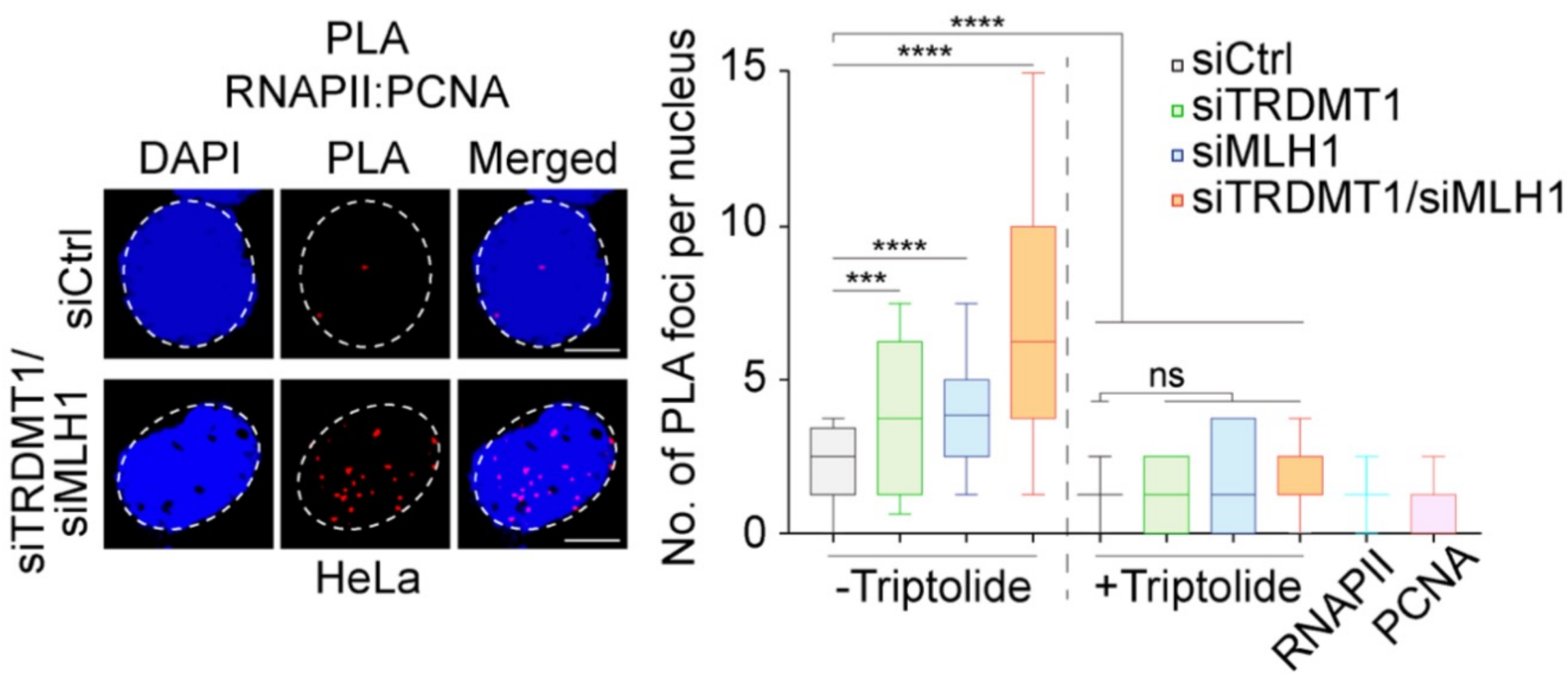

Triptolide; 1uM; 4h

Rebuttal Fig. 14

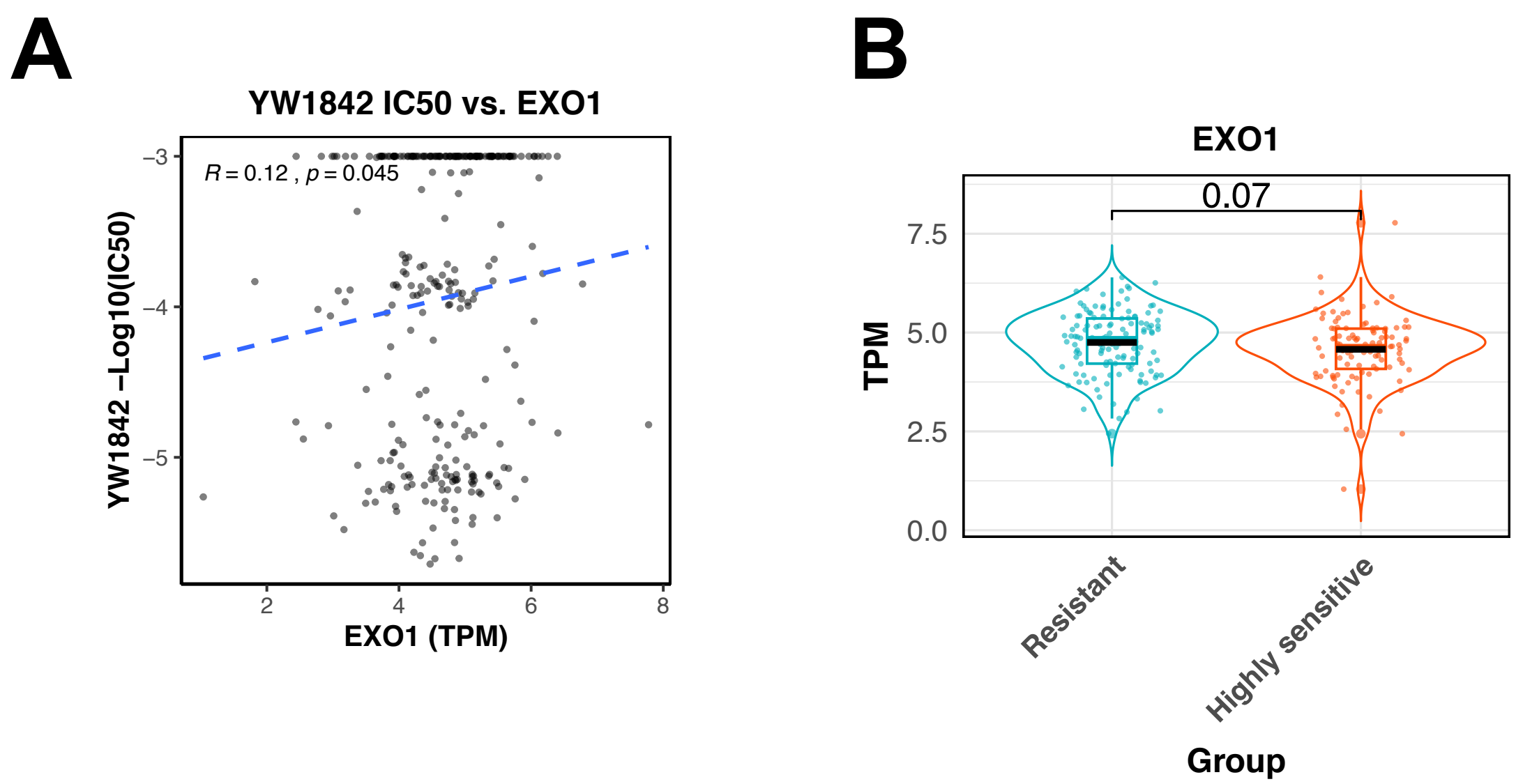

Rebuttal Fig. 15

A

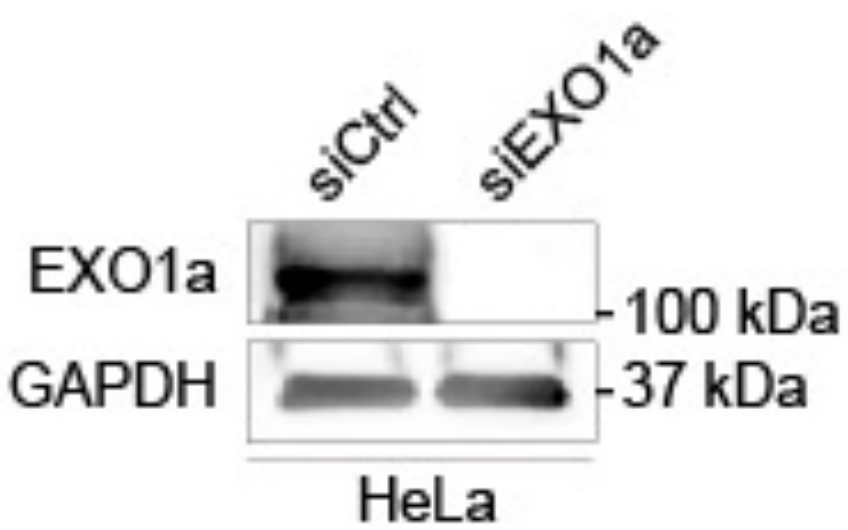

B

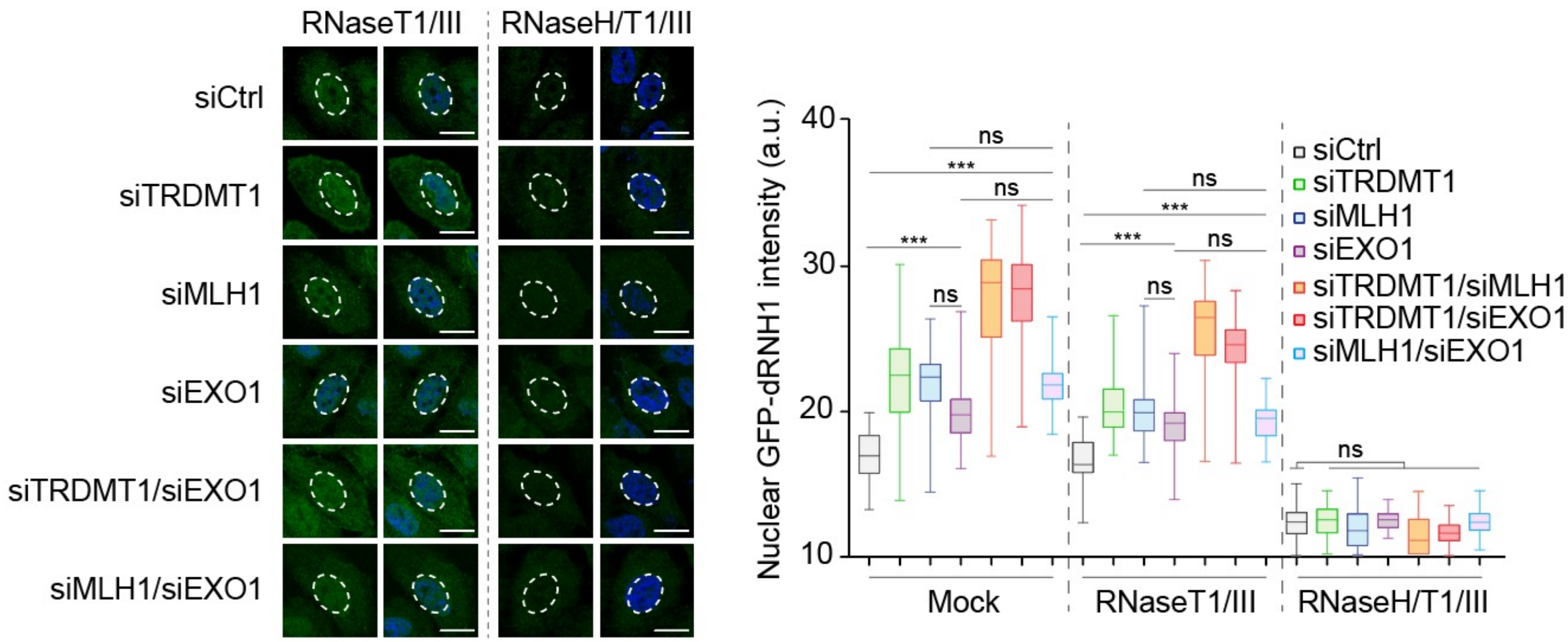

Rebuttal Fig. 16

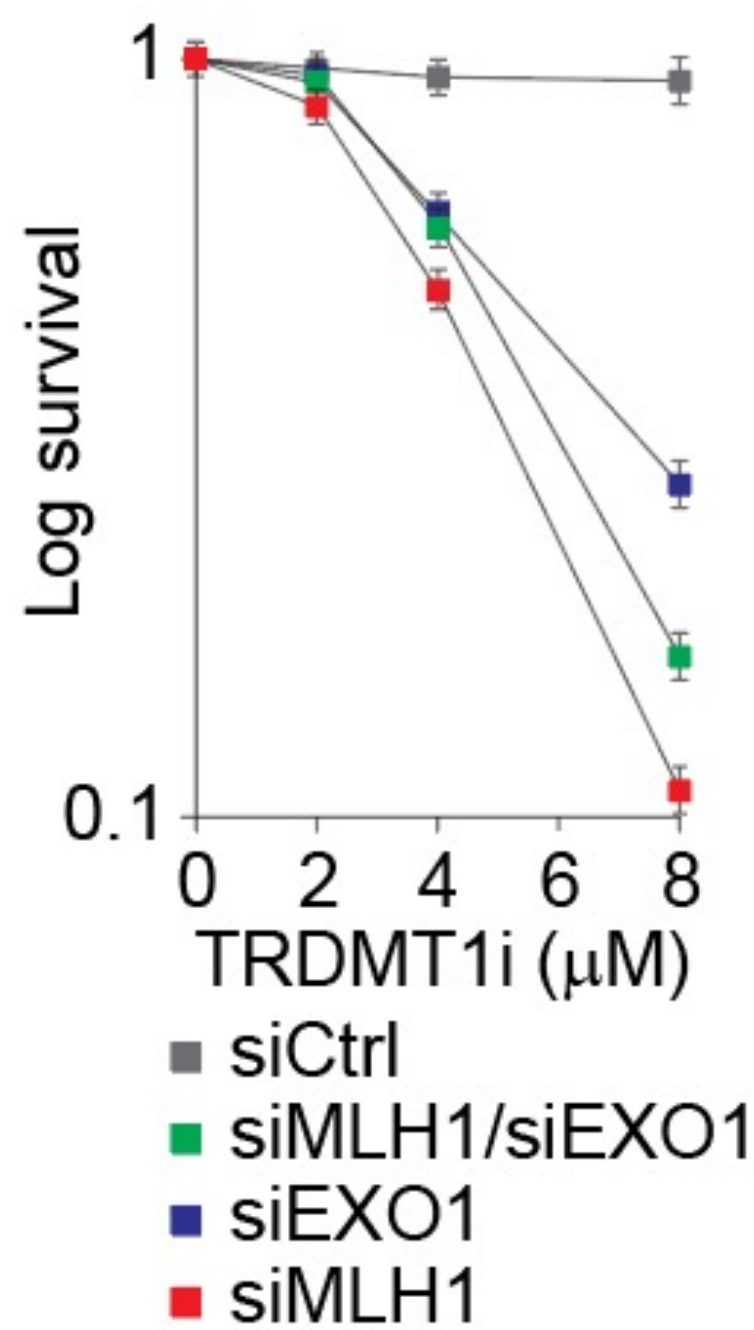

Rebuttal Fig. 17

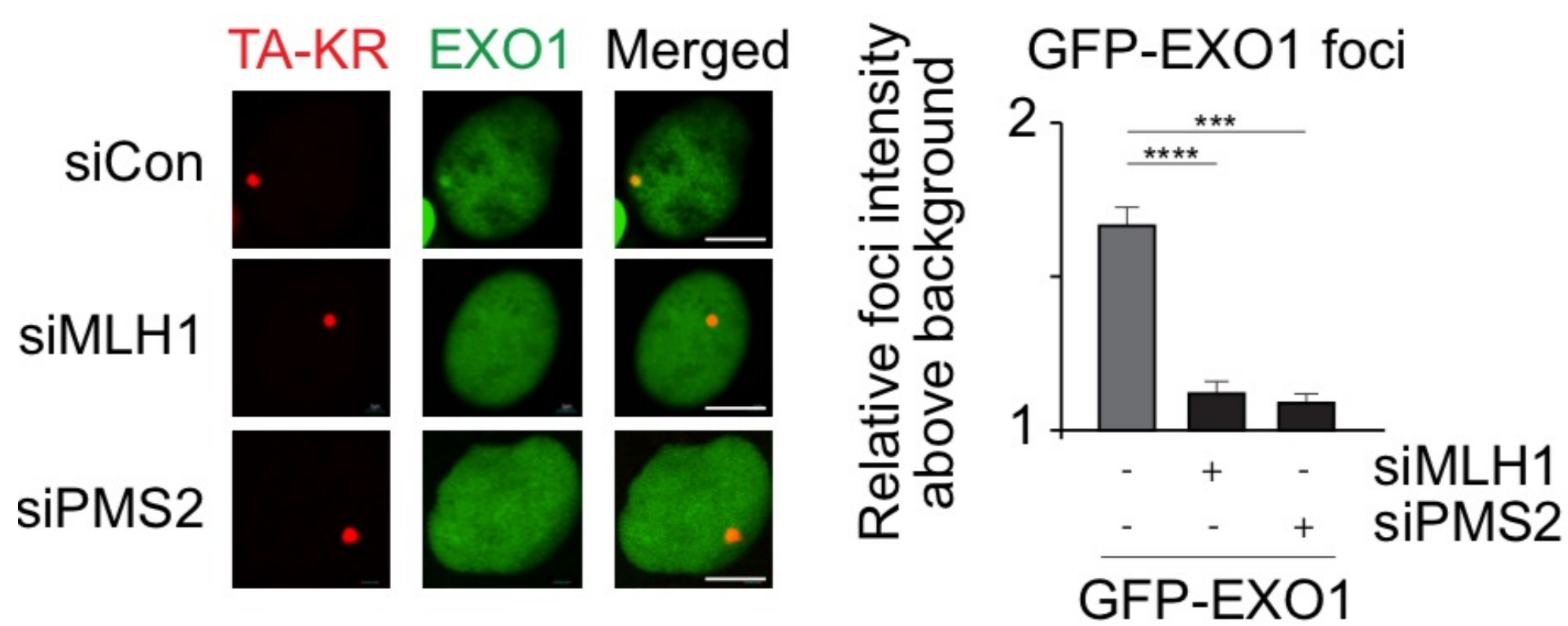

Rebuttal Fig. 18

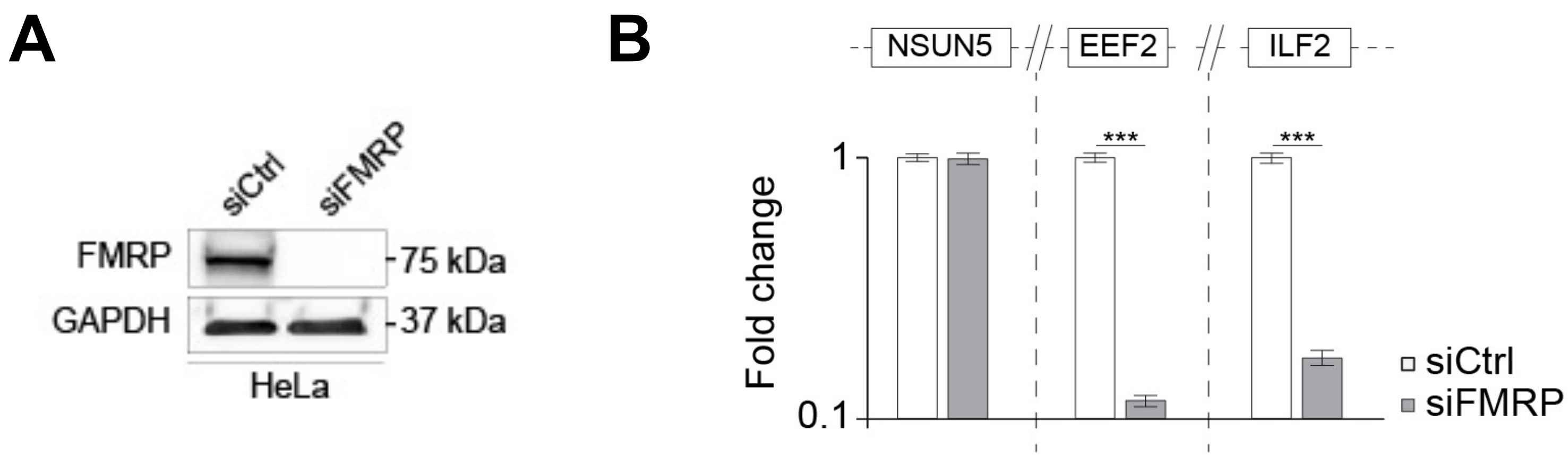

Point-by-point responses to reviewers' comments

Reviewer #1 (Remarks to the Author):

The revised manuscript has been greatly improved, and the authors have addressed most of my concerns. However, there are still some minor points that need to be corrected.
1. Figure Citations: Please verify and correct erroneous figure panel citations (e.g., Figs. S8C-D and S8E-F).
2. Nomenclature: Please correct the remaining "gH2AX" to "γH2AX" in both the manuscript text and the figures.

We thank the reviewer for his/her support to the acceptance of this manuscript. Following the new comments, we have corrected the citations of Figs. S8C-D and S8E-F), and changed all "gH2AX" to "γH2AX".

Reviewer #2 (Remarks to the Author):

The authors provide satisfactory responses to my concerns regarding the conclusions based on PLA by adding S9.6 immunoprecipitation and PCNA accumulation analyses. Moreover, they demonstrate that the RNAPII inhibitor Triptolide can reduce TRC. In addition, they use mutant isoforms of MLH1 and PMS2 in complementation experiments to better demonstrate the mechanism of action of these two factors in R-loop accumulation and harmful TRC, in coordination with the m5C RNA methyltransferase TRDMT1.

I therefore support publication of this manuscript in Nature Communications.

We thank the reviewer for his/her support to the acceptance of this manuscript.

Reviewer #3 (Remarks to the Author):

Recommendation: Accept in its current form
I have carefully reviewed the revised manuscript and the authors' point-by-point responses to the reviewers' comments. The authors have substantially strengthened the manuscript through the addition of several new experiments and expanded mechanistic analyses.

In particular, the authors have addressed the major concern regarding the non-canonical role of MutLα in R-loop suppression by demonstrating that both the ATPase activity of MLH1 and the endonuclease activity of PMS2 are required for R-loop removal, suppression of transcription–replication conflicts, maintenance of replication fork progression, and resistance to TRDMT1 inhibition. While direct biochemical reconstitution of MutLα-mediated R-loop processing remains an important future direction, the newly presented genetic and functional evidence significantly strengthens the proposed model.

The authors have also provided important new data implicating EXO1 as a downstream effector of MutLα in R-loop resolution. The epistasis analyses, TRDMT1 inhibitor sensitivity studies, and recruitment experiments collectively support a functional MutLα–EXO1 pathway that suppresses R-loop-associated genomic instability. These findings considerably enhance the mechanistic depth and conceptual impact of the study.

The authors have also adequately addressed the minor concerns regarding nomenclature, figure presentation, data interpretation, and methodological details.

Overall, the revised manuscript presents a compelling and mechanistically well-supported study identifying a therapeutically actionable synthetic lethal interaction between TRDMT1 and MutLα deficiency. The work advances our understanding of how RNA modification pathways cooperate with DNA repair factors to suppress transcription–replication conflicts and maintain genome stability. The findings have clear translational relevance for the treatment of MLH1/PMS2-deficient cancers.

We thank the reviewer for his/her support to the acceptance of this manuscript.
