# Supplementary material for: Suppression of transcription-replication conflicts by sequence-coordinated actions of TRDMT1 and MutLα
Source: Nat Commun. 2026 Aug 10;17:9566. doi: 10.1038/s41467-026-76137-8 (PMC13550614; doi:10.1038/s41467-026-76137-8)

**Related to Fig 5**

Fig 5D

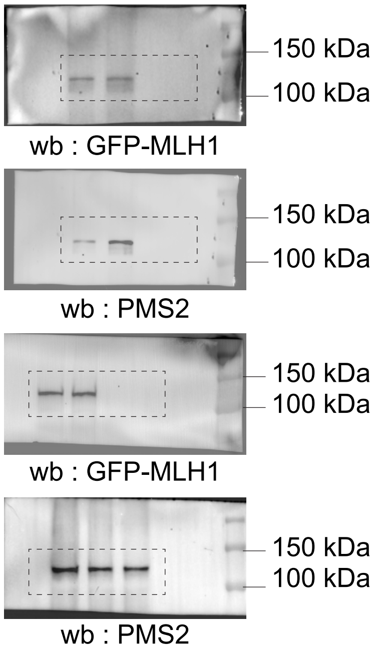

**Related to Fig S2**

Fig S2B

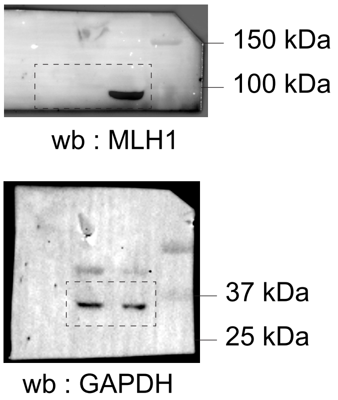


Fig S2E

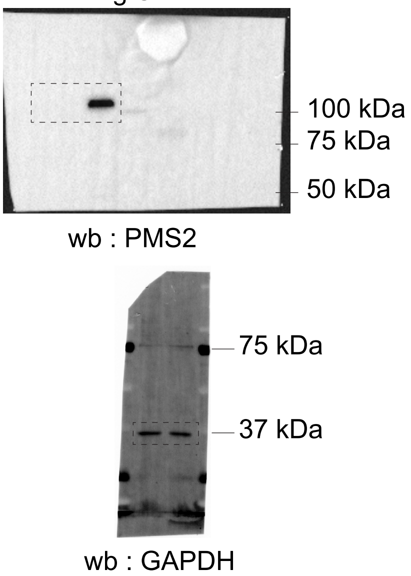


Fig S2H

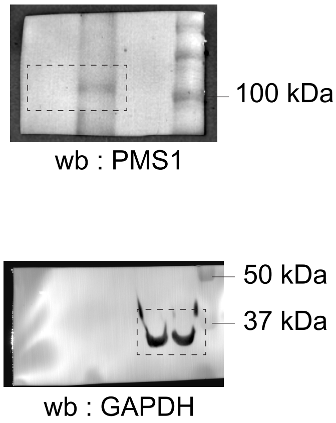


Fig S2K

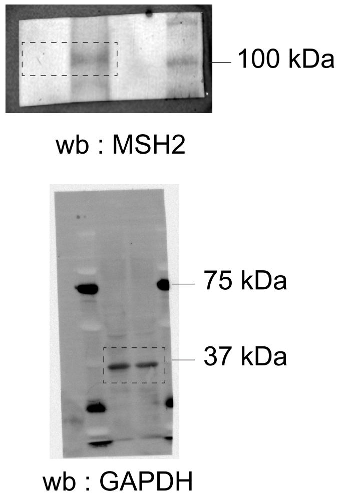


Fig S2C

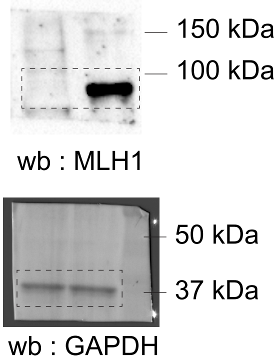


Fig S2F

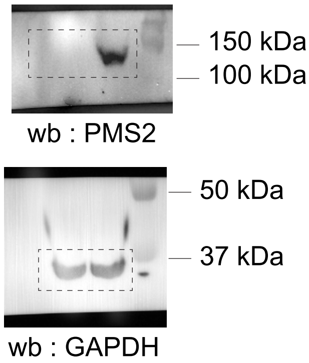


Fig S2I

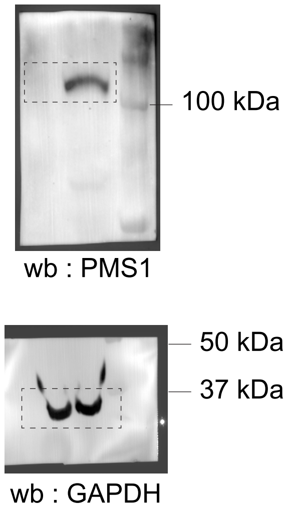


Fig S2L

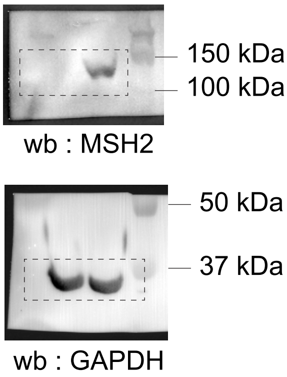

**Related to Fig S3**

Fig S3B

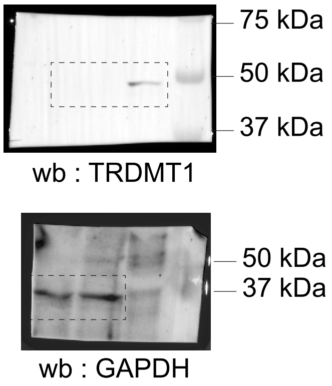


Fig S3A

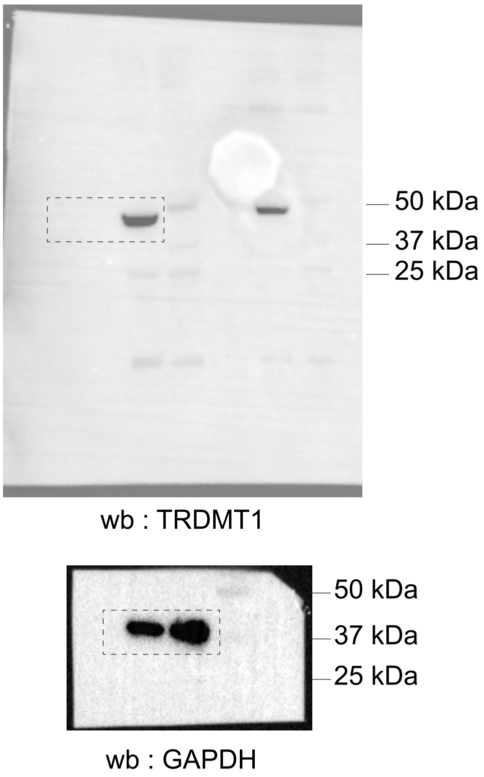


Fig S3D

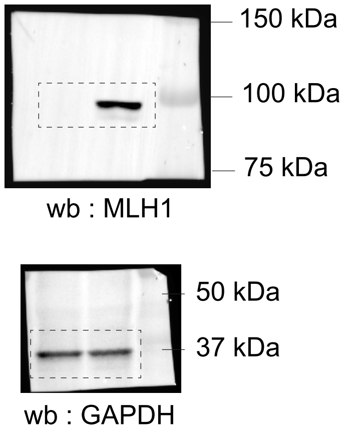


Fig S3C

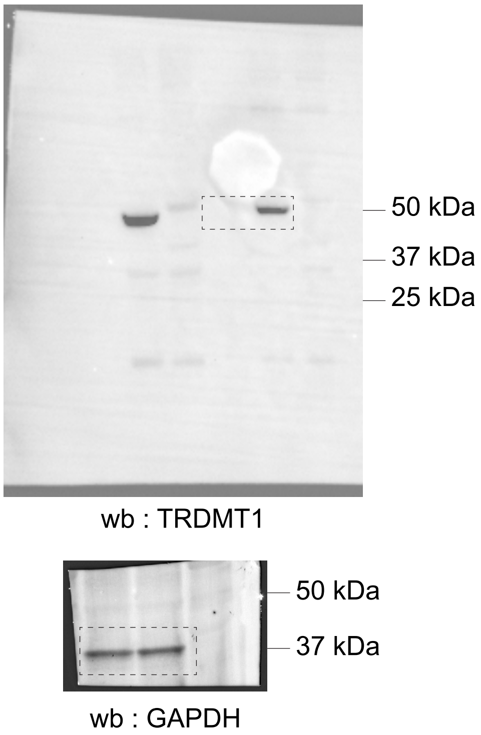


Fig S3H

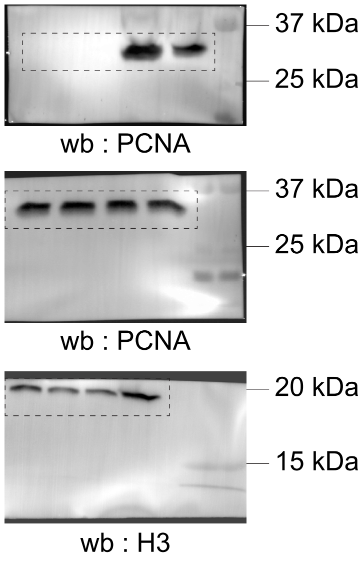

Fig S4B

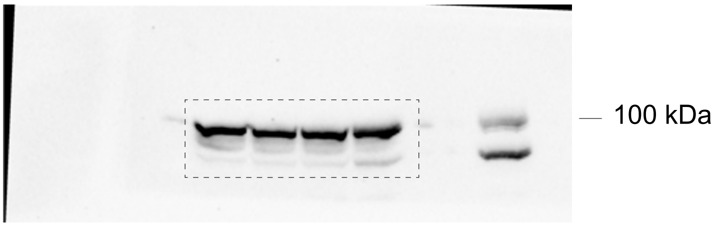


wb : PARP1

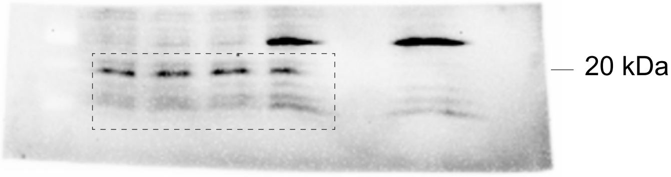


wb : Cleaved Caspase 3

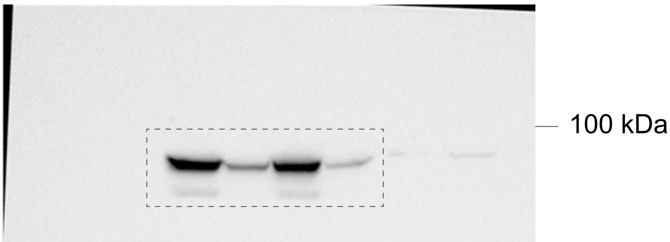


wb : MLH1

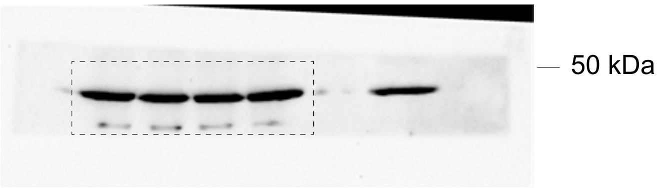


wb : TRDMT1

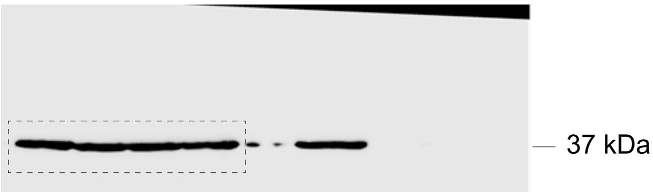


wb : GAPDH

# Related to Fig S6

Fig S6A

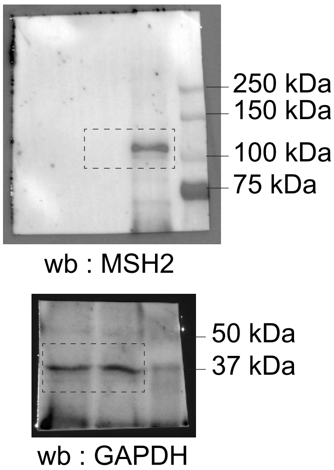


Fig S6B

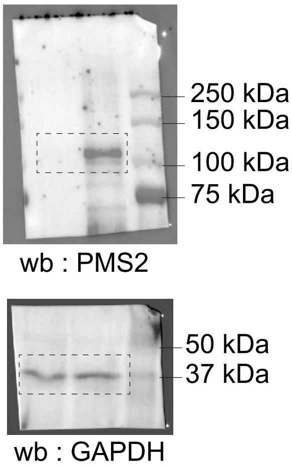


Fig S6C

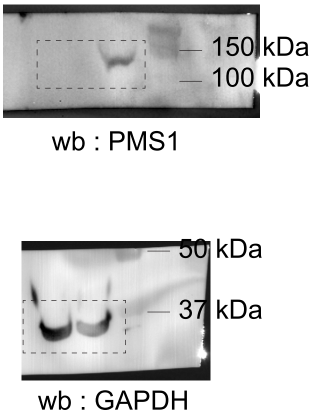

## Related to Fig S7

Fig S7D

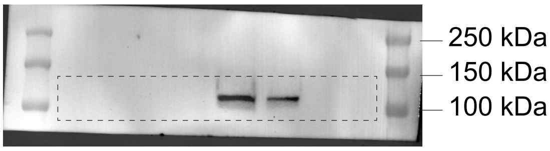


wb : GFP-MLH1

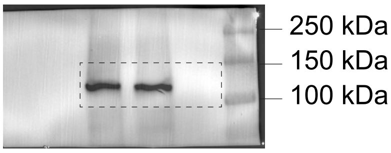


wb : GFP-MLH1

Fig S7E

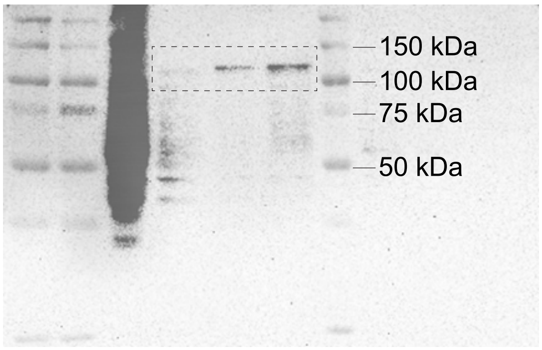


wb : GFP-MLH1

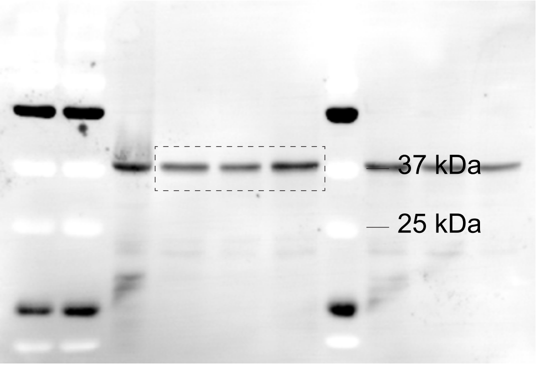


wb : GAPDH

Fig S7H

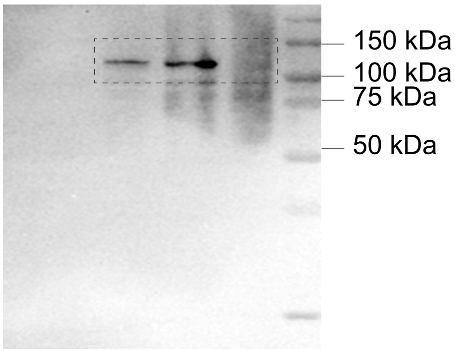


wb : GFP-MLH1

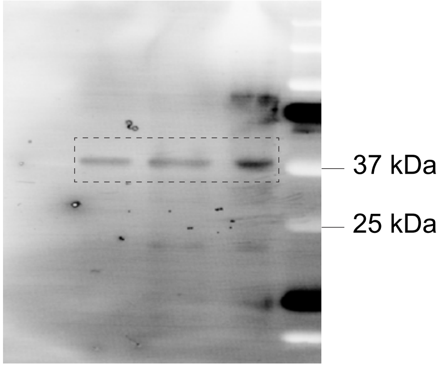


wb : GAPDH

# Related to Fig S8

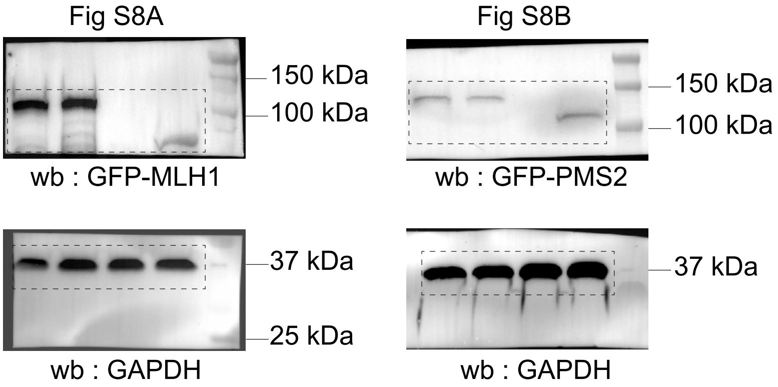

Fig S9C

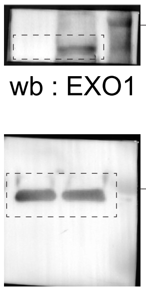

Supplement: Supplementary file 4 [file 41467_2026_76137_MOESM4_ESM.zip › Uncropped and unprocessed blots.pdf]
